# Advances in Artificial Intelligence for Wrist Joint Injury Diagnosis

**DOI:** 10.7150/ijms.134301

**Published:** 2026-07-01

**Authors:** Hong Li, Xilin Liu

**Affiliations:** 1 Department of Nursing, China-Japan Union Hospital of Jilin University, Changchun, 130033, China.; 2 Department of Hand and Foot surgery, China-Japan Union Hospital of Jilin University, Changchun, 130033, China.

**Keywords:** artificial intelligence, wrist joint injuries, diagnosis, ligament damage, convolutional neural networks

## Abstract

This study provides a comprehensive analysis of the application of artificial intelligence (AI) in diagnosing acute traumatic wrist joint injuries (WJIs), including fractures and ligament damage. AI has demonstrated significant potential in identifying fractures and ligament damage. The study highlights the use of various AI technologies and algorithms, including Convolutional Neural Networks (CNNs), Gradient Class Activation Mapping (Grad-CAM), deep learning models, object detection models, automated assessment algorithms, traditional machine learning techniques, data augmentation and preprocessing, Natural Language Processing (NLP), and integration with other imaging modalities. Compared with traditional diagnostic methods, AI offers substantial benefits, such as efficient processing of large datasets, minimizing diagnostic errors and missed cases, aiding in interpreting complex fracture patterns, optimizing workflow, enhancing diagnostic efficiency, and providing comprehensive diagnoses through multimodal integration. AI has significantly improved the precision and efficiency of fracture detection, reduced unnecessary imaging procedures, expedited the diagnostic and reporting process, optimized resource allocation, and improved patient outcomes. However, clinical application of AI faces challenges, including ethical considerations, regulatory hurdles, data privacy and security issues, algorithm transparency and interpretability problems, and unclear liability definitions. The future of AI in diagnosing WJIs is promising, with potential advancements in fracture detection, treatment planning, and rehabilitation strategies. However, challenges such as model validation and training of healthcare professionals must be addressed to fully integrate AI into orthopedic practice and advance the management of WJIs.

## 1. Introduction

Artificial intelligence (AI) is transforming the diagnosis of wrist joint injuries (WJIs), improving diagnostic accuracy and operational efficiency for detecting fractures and ligament injuries. Recent studies have systematically evaluated AI-based models' applicability and diagnostic performance in identifying wrist fractures, representing some of the most prevalent injuries encountered in clinical practice 1-3. AI is increasingly applied in medical imaging, with growing evidence supporting its use in fracture detection. Three clinically prevalent fracture types, including distal radius, ulnar styloid, and scaphoid fractures, are common in emergency settings and are often missed on initial radiographs. AI has shown potential to improve diagnostic accuracy and reduce missed injuries. This review provides a comprehensive analysis of AI technologies for WJIs, synthesizes available evidence on diagnostic performance, and discusses clinical benefits, challenges, and future directions.

Advancements in AI highlight its potential to play a crucial role in the early diagnosis and management of ligament injuries, thereby reducing the risk of progression to degenerative conditions 4. Moreover, the application of AI in ultrasound imaging has been investigated as a novel approach for detecting wrist fractures. Another study evaluated the feasibility of combining three-dimensional ultrasound (3DUS) with AI for automated fracture identification. The findings demonstrated that AI-based analysis performed on par with human interpretation, achieving a sensitivity of 1.0 in fracture identification. This remarkable sensitivity suggests that AI-based ultrasound could provide a non-invasive and radiation-free alternative option to conventional radiography, particularly benefiting pediatric patients by minimizing unnecessary exposure to ionizing radiation 5,6. Collectively, AI offers remarkable potential to transform the diagnosis and management of wrist injuries by improving the detection of fractures and ligament injuries. This technology not only supports clinical decision-making but also reduces the workload on healthcare providers and enhances patient outcomes. As research in this field advances, the use of AI in routine orthopedic practice may become more promising, providing a valuable tool for enhancing diagnostic accuracy and efficiency.

## 2. Artificial intelligence technologies and algorithms in wrist injury diagnosis

The application of AI and algorithm-driven approaches in the medical field has significantly advanced diagnostic capabilities across various conditions (Table 1). In musculoskeletal injuries, particularly wrist injuries, AI has remarkable potential to enhance diagnostic precision and clinical outcomes. Studies have found AI integration in various imaging modalities and diagnostic strategies to improve the identification and assessment of wrist injuries. In a study by Ozkaya *et al*. 7 the diagnostic efficacy of AI, specifically CNNs, was assessed for identifying scaphoid fractures in anteroposterior wrist radiographs. The study's results demonstrated the potential of AI in accurately identifying fractures, highlighting the application of AI in improving diagnostic capabilities for wrist injuries. Furthermore, Zhang *et al*. 5 focused on the diagnostic accuracy of 3DUS and AI in detecting pediatric wrist injuries. The study found that AI interpretation was similar to human interpretation, with a sensitivity of 1.0 in detecting fractures. This suggests that AI can play a crucial role in promoting the accuracy and reliability of diagnosing wrist injuries in pediatric patients. Similarly, Hendrix *et al*. conducted a study to compare the diagnostic performance of an AI algorithm with that of experienced musculoskeletal radiologists in identifying scaphoid fractures using conventional multi-view radiographs of the hand and wrist 8. His research aimed to assess whether AI could support radiologists in making more accurate diagnoses. Their findings highlighted AI's potential to perform at a level comparable to musculoskeletal radiologists in detecting scaphoid fractures, emphasizing its value as a supportive tool for healthcare professionals in diagnosing wrist injuries.

**Figure 1:**
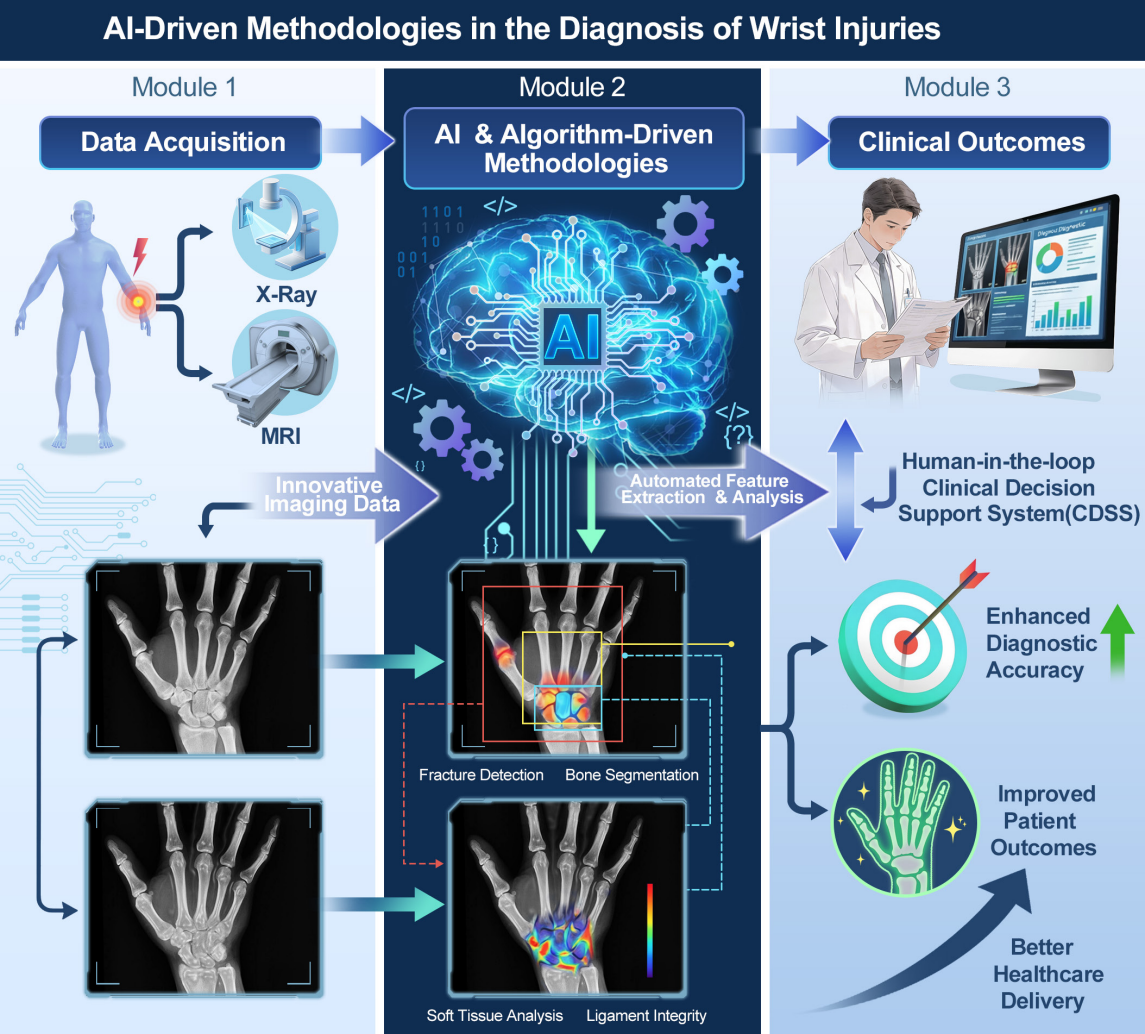
AI Technologies in Wrist Injury Diagnosis. The process begins with data acquisition from X-ray and MRI, followed by AI-based feature extraction and analysis, ultimately leading to improved diagnostic accuracy, patient outcomes, and healthcare delivery.

A comprehensive review of the literature underscores the increasing prominence of artificial intelligence and algorithm-driven methodologies in the diagnosis of wrist injuries. Collectively, these studies highlight the potential of AI to enhance diagnostic accuracy, improve outcomes, and aid healthcare professionals in effectively identifying and managing wrist injuries through innovative imaging and diagnostic approaches (Fig. 1).

CNNs are among the most extensively used AI algorithms for image classification, including detecting wrist fractures. These networks are highly effective in extracting features from images, enabling the recognition of intricate patterns indicative of fractures. Studies have shown that CNNs can attain high levels of sensitivity and specificity in identifying wrist injuries, often outperforming conventional diagnostic techniques 3,25.

CNNs have emerged as a widely used approach for image classification across various domains, including medical imaging. Within medical image analysis, CNN architectures are extensively employed for several tasks, pattern recognition and disease diagnosis 26. Particularly, CNNs have been used to identify and classify fractures in medical images, including X-rays of extremity bones 27. Recent studies have shown that AI-driven algorithms, particularly CNNs, can effectively detect and classify fractures of the wrist and long bones in X-ray imaging with high accuracy 25. The use of deep learning, including CNNs, in medical image analysis has enabled the development of diagnostic models for various medical conditions, including fractures 27. Deep learning models, once trained, can be used for tasks such as image classification, making them valuable tools for healthcare professionals 28.

The advancement of deep learning (DL) methodologies, particularly CNNs, has significantly contributed to the development of sophisticated diagnostic models for various medical conditions, including fractures. Once trained, these models exhibit a high degree of accuracy in image classification, rendering them indispensable tools for healthcare professionals 28. Furthermore, advancements in AI, particularly the optimization of CNNs using bio-inspired algorithms, have led to significant improvements in medical imaging applications such as breast cancer detection in infrared scans 29. In orthopedics, machine learning is increasingly utilized for diagnosing fractures and predicting their outcomes, providing valuable decision-making support for clinicians 30. A promising development is the integration of multimodal AI, which combines imaging data with clinical information to improve diagnostic accuracy 31. Further, deep learning-based object detection algorithms have enabled automated recognition of various medical conditions, including fractures, by leveraging neural network architectures 32. The role of AI in medical image analysis, mainly through CNNs, is crucial in improving diagnostic efficiency and accuracy, especially for wrist fracture detection. These advancements provide potent tools for medical professionals, facilitating more reliable disease detection and classification 33. As research progresses, the continued refinement of deep learning models is expected to enhance diagnostic capabilities further, thereby improving patient outcomes in orthopedics and other medical fields.

### 2.1. Deep learning models

Deep learning architectures, particularly those integrating CNNs with long short-term memory (LSTM) networks, have been developed to improve fracture detection accuracy. For instance, a fused model combining a dilated CNN and LSTM was proposed for wrist X-ray analysis, leading to enhanced diagnostic performance 34,35. This approach enables both deep feature extraction and temporal data analysis, which is crucial for understanding wrist injury dynamics. Deep learning methods have been widely explored across various medical imaging domains, including fracture detection. For example, a diagnostic support system for distal humerus fractures was developed using deep learning and X-ray images, further illustrating its value in musculoskeletal diagnostics 36. Moreover, CNN-based object detection was applied to security cameras and smart devices, emphasizing the role of pre-trained models in improving accuracy 37. Similarly, a residual-based multi-stage deep learning framework was introduced for Alzheimer's disease detection, reinforcing the effectiveness of deep neural networks in medical diagnostics 38. The reviewed literature demonstrates the increasing adoption of deep learning across multiple fields, particularly in medical imaging, security, and agriculture. These studies collectively highlight its potential to enhance diagnostic accuracy, predictive analytics, and object detection.

### 2.2. Gradient class activation mapping (Grad-CAM)

The Grad-CAM technique, a widely used Explainable Artificial Intelligence (XAI) method, visualizes image regions that significantly influence an AI model's classification process. By providing insights into the specific features that contribute to wrist fracture detection, Grad-CAM facilitates a deeper understanding of the model's decision-making 39,40. This level of transparency is essential for clinical applications, as it enhances confidence in AI-assisted diagnostic systems and supports their integration into medical practice. The use of AI algorithms for wrist fracture detection has been a topic of interest in recent research. Till *et al*. 40 focused on the development and optimization of AI algorithms specifically for wrist fracture detection in children. The application of heat maps, particularly Grad-CAM, has proven to be an effective explainable AI technique for detecting wrist fractures in X-ray images. Studies utilizing emergency department datasets have demonstrated their effectiveness in highlighting crucial image features relevant to fracture identification 41. This method allows for the visualization of the most relevant features for fracture detection in X-ray images. For instance, research on AI-based analysis of distal radius fractures has emphasized the significance of detecting key fracture-related characteristics to ensure accurate classification 42, with Grad-CAM emerging as a key tool for identifying critical image regions. In another study, deep learning models successfully learned to detect wrist fractures by generating activation maps that localized fracture-specific features. In another study, deep learning models were successfully trained to detect wrist fractures by generating activation maps that localized fracture-specific features 43. Furthermore, transfer learning has been explored to enhance fracture detection, where pre-trained features from other tasks were adapted for wrist and humerus classification 44. Integrating transfer learning with Grad-CAM provides valuable insights into the most relevant features across different bone structures. Overall, methods like CAM and Grad-CAM enhance the interpretability of deep learning models by visualizing significant activation regions linked to fracture detection 45. Advancing these AI-driven algorithms could lead to more accurate and efficient diagnostic tools for wrist fractures.

### 2.3. Object detection models

The application of advanced object detection frameworks, such as You Only Look Once (YOLO), to wrist radiograph analysis has shown potential for significant improvements in fracture detection. These models trained using large annotated image datasets, can accurately and quickly locate fractures, potentially reducing the time radiologists spend on image review. The increasing adoption of object detection frameworks in medical imaging has led to significant advancements in fracture detection, with YOLO emerging as a prominent tool for analyzing radiographs. As a CNN-based detection system, YOLO has been particularly effective in identifying and localizing fractures, especially in pediatric wrist radiographs 33. The development of dedicated datasets for fracture classification, localization, and detection further supports its growing role in this domain 46. Recent studies have demonstrated the capabilities of YOLOv10 in automating pediatric wrist fracture detection, reflecting an ongoing shift toward automation in radiological analysis 47. Furthermore, YOLO's utility extends beyond fracture detection to object localization and orientation estimation in medical imaging. Using oriented bounding boxes (OBBs) within the YOLO framework has facilitated improved localization and orientation assessment, highlighting its adaptability to complex medical imaging challenges 48. The application of YOLOv3 in medical imaging has been explored extensively, with studies demonstrating its efficacy in detecting rib fractures on chest radiographs 49. The integration of YOLO-based object detection with EfficientNet's classification network has also been utilized to automatically classify distal radius fractures, reinforcing the role of deep learning in radiographic fracture detection 50. Moreover, cross-center validation studies have assessed YOLO-based deep learning algorithms for musculoskeletal radiography, providing further evidence of their diagnostic utility 51. These findings suggest that object detection frameworks such as YOLO provide a robust approach for fracture detection and localization in radiographic imaging. The efficiency and accuracy of YOLO in identifying fractures across various radiograph types enhance its utility in automating and optimizing diagnostic workflows 52. Furthermore, object detection methodologies have demonstrated superior performance to traditional segmentation-based fracture detection techniques, highlighting YOLO's role in advancing medical imaging technologies 53. With the continuous expansion of YOLO's applications in medical imaging, its impact on automated fracture detection and localization is expected to grow further 52.

### 2.4. Automated assessment algorithms

Several studies have developed automated algorithms to assess the integrity of wrist ligaments, particularly the scapholunate ligament. These algorithms use deep learning methods to analyze radiographic images and correlate findings with clinical outcomes, aiding early decision-making in patient management 4. Evaluating the integrity of wrist ligaments, particularly the scapholunate ligament (SL), is vital for diagnosing injuries and preventing long-term complications such as degenerative arthritis. Researchers have increasingly explored deep learning-based automation for ligament assessment, Keller *et al*. 4 designed an AI-driven algorithm to measure SL distance on radiographs, showing a significant correlation with arthroscopic results. Assessing wrist ligament integrity may be improved through automated methods. Radke *et al*. showed the potential of this approach by using CNNs to post-process real-time MRI images for quantifying wrist movement 54. The development of reliable automated assessments is increasingly recognized as a valuable tool for diagnosing scapholunate ligament injuries 4, addressing a key need in current clinical practice. Deep learning algorithms offer a promising approach to evaluating wrist ligament integrity, especially the scapholunate ligament, potentially improving clinical decisions for wrist injury management.

### 2.5. Machine learning techniques

In addition to deep learning, traditional machine learning techniques, such as support vector machines (SVMs) and random forests, have been used for fracture detection. Their effectiveness is enhanced when paired with preprocessing methods that improve image quality and facilitate feature extraction 25,34. Integrating machine learning algorithms into medical research has significantly advanced diagnostic accuracy and predictive modeling. In medical applications, including the detection of wrist fractures, machine learning algorithms such as SVM and Random Forests have considerable potential to improve diagnostic accuracy and decision-making processes 55,56. The role of SVM in neurology was further exemplified by Xu *et al*. 57 who used wrist signal data to recognize tonic-clonic seizures, indicating its utility in seizure monitoring and prediction. Meanwhile, Mohanty *et al*. recommended SVM for detecting fractures from X-ray images, reinforcing its potential in radiological diagnostics 56. These findings demonstrate the need to effectively utilize advanced technologies to improve patient care and healthcare results.

### 2.6. Data augmentation and preprocessing

Enhancing the performance of AI models often necessitates using data augmentation techniques. These methods generate additional training samples by modifying existing images through rotation, scaling, and flipping transformations. This process not only mitigates class imbalances but also contributes to the development of more generalized and resilient models capable of performing well across diverse datasets 34,35.

Data augmentation plays a significant role in improving AI model accuracy, particularly in wrist fracture detection. Ahmed and Manaf optimized the YOLOv8 algorithm for pediatric wrist trauma X-ray analysis 47. Another study by Zhang *et al*. 58 applied data augmentation to increase dataset variability, thereby enhancing the performance of transfer-learning models in detecting wrist fractures. Beyond orthopedic applications, the benefits of augmentation extend to dentistry, where it has improved deep-learning model accuracy. Addressing class imbalance through techniques such as data augmentation significantly improves AI model performance across various medical imaging applications. In the context of wrist fracture detection, data augmentation has been shown to enhance diagnostic accuracy by increasing dataset diversity and minimizing overfitting. These improvements contribute to AI-assisted diagnostic tools' overall reliability and clinical applicability.

### 2.7. Natural language processing (NLP)

NLP in radiology has advanced diagnostic capabilities, particularly in fracture detection. Jungmann *et al*. used NLP to evaluate the temporal patterns of fracture diagnoses, particularly during the COVID-19 pandemic, providing insights into shifts in clinical presentation and healthcare utilization 59. Chekmeyan *et al*. 60 introduced Cross-Check QA, a quality assurance framework designed to identify inconsistencies between AI-driven and radiologist interpretations of high-acuity CT scans, underscoring the role of AI in enhancing diagnostic reliability. Zech *et al*. 61 focused on the use of artificial intelligence to identify fractures in pediatric and young adult upper extremity radiographs. The study emphasized the challenges in detecting pediatric fractures due to the unique response of the pediatric skeleton to injuries. This suggests the need for specialized approaches when applying AI in fracture diagnosis for different age groups. The NLP has been explored for its role in analyzing radiology reports to aid fracture detection. One study highlighted its potential in extracting critical diagnostic information from these reports 62. NLP applications extend beyond textual radiology reports, as machine learning-based approaches have been utilized to analyze Dutch radiology records of extremity injuries and chest radiographs, enabling the identification of fractures and pneumothorax 63. This demonstrates NLP's adaptability in processing diverse medical datasets for diagnostic use. A review analyzed its role in orthopedic traumatology, particularly in fracture detection 64 This review emphasizes the critical role of NLP in advancing the precision of orthopedic fracture diagnosis. The integration of NLP methodologies into fracture diagnostic workflows offers a promising avenue for improving the accuracy and efficiency of these processes. By utilizing NLP algorithms to analyze radiology reports and other medical data, healthcare professionals can refine their ability to identify and classify fractures accurately. The studies indicated that NLP contributes substantially to improving fracture diagnosis by augmenting traditional diagnostic methods and AI-based systems.

### 2.8. Integration with other imaging modalities

AI algorithms are increasingly integrating with other imaging techniques, such as computed tomography (CT) and magnetic resonance imaging (MRI), to provide a more comprehensive assessment of wrist injuries. This multimodal approach can enhance diagnostic accuracy and facilitate better treatment planning 25. AI in diagnosing wrist injuries uses various sophisticated algorithms and methodologies, particularly deep learning models such as CNNs, object detection frameworks, and automated assessment algorithms. These advanced tools increase diagnostic precision and streamline radiological evaluations, improving patient outcomes.

AI algorithms integrated with imaging techniques such as CT and MRI have shown potential in enhancing the comprehensive assessment of medical conditions. In orthopedics, AI has been used for fracture diagnosis in X-rays, improving accuracy and efficiency. It has also been combined with CT and MRI to provide detailed evaluations of wrist injuries 25. The increasing use of medical imaging techniques, mainly CT and MRI, has resulted in a rise in incidental findings, underscoring the need for advanced technologies like AI to enhance interpretation and diagnosis 65. AI can integrate data from multiple imaging modalities, including echocardiography, CT, MRI, and PET, enabling a comprehensive evaluation of conditions such as cardiovascular diseases 66. Despite the advantages of traditional imaging methods, they have some limitations. For instance, MRI segmentation techniques for shoulder injuries may not fully capture the extent of tissue damage, limiting their diagnostic utility. AI-based analytical models can address these challenges by offering enhanced image interpretation, facilitating more precise injury characterization, and improving clinical outcomes 67. The combination of different imaging methods, such as fMRI alongside EEG and MEG, provides a more detailed and holistic perspective on brain function, emphasizing the strengths of multimodal imaging 68. In summary, incorporating AI-driven algorithms into imaging techniques like CT and MRI can significantly improve diagnostic precision and efficiency, facilitating more accurate evaluations of various medical conditions, including wrist injuries.

### 2.9. Quantitative synthesis of AI diagnostic performance for wrist fractures

Several recent meta-analyses have provided pooled estimates of AI diagnostic performance for wrist fracture detection (Table 2). A systematic review and meta-analysis by Suen *et al*. included 20 studies and reported that the pooled sensitivity and specificity for AI in detecting distal radius fractures were 0.92 (95% CI: 0.88-0.95) and 0.89 (95% CI: 0.84-0.92), respectively; for human readers, the corresponding values were 0.95 (95% CI: 0.91-0.97) and 0.94 (95% CI: 0.91-0.96) 69. For scaphoid fractures, the pooled sensitivity and specificity for AI were 0.85 (95% CI: 0.73-0.92) and 0.83 (95% CI: 0.76-0.89), whereas human readers exhibited 0.71 (95% CI: 0.66-0.76) and 0.93 (95% CI: 0.90-0.95), respectively. These findings indicate that AI performance is comparable to human readers for distal radius fractures, while for scaphoid fractures, AI demonstrates higher sensitivity but lower specificity than human experts.

A complementary meta-analysis by Hansen *et al*., which focused specifically on DL algorithms using CNNs, included six studies comprising 33,026 radiographs and found a summary sensitivity of 92% (95% CI: 80-97%) and a summary specificity of 93% (95% CI: 76-98%) for CNN-based models compared with a reference standard of healthcare expert consensus 70.

For hand and wrist fractures more broadly, a meta-analysis by Wong *et al*. of 36 studies reported that AI models achieved an area under the curve (AUC) of 0.946 (95% CI not reported), a positive likelihood ratio (LR+) of 7.69 (95% CI: 6.40-9.19), and a negative likelihood ratio (LR-) of 0.11 (95% CI: 0.08-0.15) 71.

A separate meta-analysis of 26 studies assessing AI assistance for clinicians found that AI support significantly improved pooled sensitivity from 77% (95% CI: 72-81) to 87% (95% CI: 83-90) and pooled specificity from 88% (95% CI: 85-90) to 92% (95% CI: 89-94), with the corresponding AUC increasing from 0.90 (95% CI: 0.87-0.92) to 0.95 (95% CI: 0.93-0.97) 72.

These pooled estimates confirm that AI, particularly CNN-based deep learning models, achieves high diagnostic accuracy for wrist fracture detection, with performance comparable to or exceeding that of human readers for certain fracture types, especially when used as an assistive tool.

### 2.10. Comparative analysis of AI architectures for wrist fracture detection

A critical comparative analysis of different AI architectures provides clinically actionable insights. The most widely applied architectures for wrist fracture detection fall into two broad categories: (1) CNNs optimized for image classification, and (2) object detection models that provide simultaneous fracture localization and classification.

CNN-based classification models treat fracture detection as a binary or multi-class classification problem, outputting a probability score without inherent spatial localization 3,70. Their strengths include architectural maturity, availability of pre-trained models for transfer learning, and excellent performance on well aligned radiographs 73. These models treat fracture detection as a binary or multi-class classification problem, outputting image-level probability scores without inherent spatial localization. While techniques such as Grad-CAM can provide approximate attention maps, these are less precise than bounding boxes for identifying multiple concurrent fractures.

Object detection models (single-stage architectures such as YOLO, and two-stage architectures such as Faster R-CNN) output bounding boxes around suspected fracture sites 10,74. This localization capability reduces cognitive load, enables counting of multiple fractures, and supports automated measurement. However, they require larger annotated datasets and are computationally more intensive.

Quantitative comparisons on the GRAZPEDWRI-DX pediatric wrist dataset provide guidance for architecture selection. Ahmed *et al*. 75 evaluated YOLOv5, YOLOv6, YOLOv7, and YOLOv8 against Faster R-CNN, finding that YOLO models consistently outperformed Faster R-CNN. Specifically, YOLOv8m achieved a fracture detection sensitivity of 0.92 and a mean average precision (mAP) of 0.95 at IoU 0.5 for the fracture class, while YOLOv8x recorded the highest overall mAP of 0.77 across all abnormality classes 75. These data confirm that single-stage object detectors offer superior performance for wrist fracture detection compared to two-stage architectures.

## 3. Effectiveness of AI in diagnosing wrist injuries: Advantages over traditional methods

**Figure 2:**
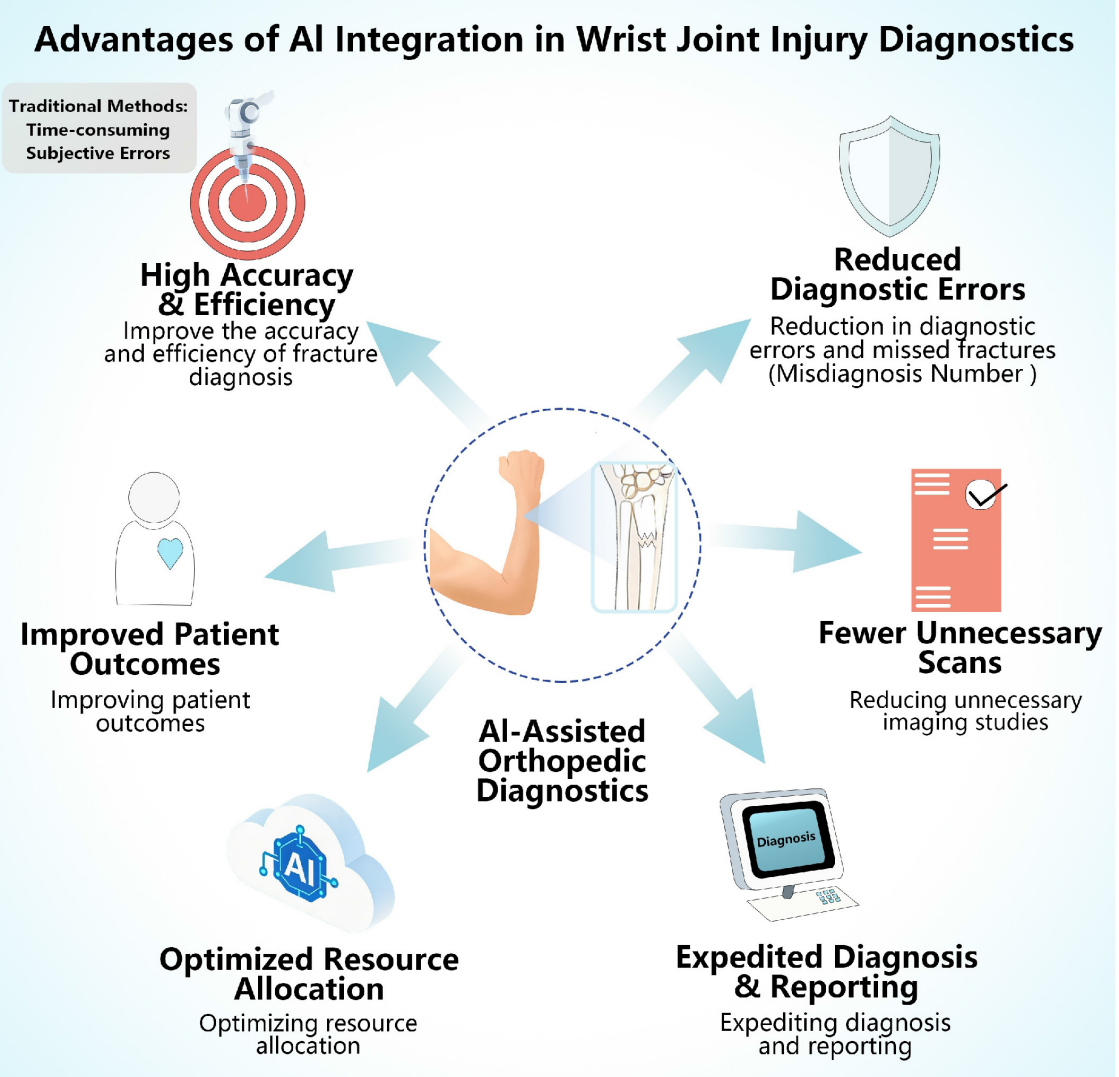
Advantages of AI in Wrist Injury Diagnosis. AI enhances diagnostic accuracy and efficiency, reduces errors and missed fractures, limits unnecessary imaging, accelerates diagnosis and reporting, optimizes resource use, and ultimately improves patient outcomes.

The application of AI in diagnosing wrist joint injuries has shown significant effectiveness, particularly in detecting common fractures such as those of the distal radius, ulnar styloid process, and scaphoid. A study demonstrated that an AI model trained on 4,432 medical images achieved high sensitivity and specificity in detecting fractures. The model yielded an AUC value of 0.903 for distal radius fractures, 0.925 for ulnar styloid fractures, and 0.808 for scaphoid fractures. When novice radiologists used the AI model as an assistive tool, their diagnostic accuracy for scaphoid fractures, frequently missed in clinical practice, improved significantly. This highlights the potential of AI to augment human performance in complex diagnostic settings 3. One of the primary advantages of AI over traditional diagnostic methods is its ability to process large datasets quickly and consistently. The conventional approach to fracture diagnosis relies on radiologists, whose evaluations are inherently subjective and may be affected by fatigue, variability in interpretation, and cognitive biases. Recent advances in AI, especially deep learning methods such as CNNs, offer a promising alternative by recognizing patterns and features in medical images that might not be immediately obvious to the human eye. This capability not only reduces the likelihood of missed diagnoses but also improves the overall accuracy of fracture detection 6,25. The application of AI in radiology enhances the interpretation of complex fracture patterns and subtle abnormalities that even expert radiologists may overlook. Scaphoid fractures, although uncommon, require rapid identification because of their potential for serious complications. Their detection on radiographs can be challenging, but AI's contribution to increasing the detection rate of such fractures is crucial. Failure to diagnose scaphoid fractures can result in severe outcomes, including non-union and avascular necrosis 3,25. AI enhances diagnostic accuracy and optimizes operational workflows in radiology departments. By automating the preliminary evaluation of radiographs, AI reduces the burden on radiologists, enabling them to focus on more complex clinical cases and decreasing the time required for image interpretation. This improvement is particularly advantageous in busy clinical environments, where the ability to provide timely diagnoses is vital for effective patient management 25,76. Integrating AI with other imaging modalities, such as CT and MRI, can provide a more comprehensive diagnostic approach. This multimodal approach enhances the precision of wrist injury classification and diagnosis, thereby contributing to improved patient outcomes 25. Despite the advantages offered by AI in clinical practice, there are significant challenges to its implementation. These include ethical considerations, regulatory issues, and the need for high-quality annotated datasets for training AI models. Overcoming these obstacles is essential for AI's safe and effective use in medical diagnostics 25,76. AI has demonstrated significant effectiveness in diagnosing wrist joint injuries, presenting clear advantages over traditional diagnostic methods (Fig. 2). These benefits encompass improved diagnostic accuracy, consistency, and operational efficiency. As AI progresses, its integration into clinical settings is poised to raise the standard of care in orthopedic diagnostics.

### 3.1. AI's Impact on the precision and effectiveness of diagnosing wrist injuries

Incorporating AI into the diagnosis of fractures has been demonstrated to significantly improve the accuracy and efficiency of diagnosing wrist injuries. AI holds the potential to improve diagnostic precision, optimize workflow, and enhance the reliability, convenience, and effectiveness of diagnostic procedures 77. Advanced AI technology enables healthcare practitioners to achieve greater diagnostic accuracy and speed when identifying wrist injuries 25. AI can also assist in minimizing missed or delayed diagnoses of hand and peripheral nerve injuries, which are often difficult to evaluate 78. Commercially available AI tools have been demonstrated to enhance the diagnostic process, significantly boosting the performance of radiologists when used alongside traditional diagnostic techniques 79. Studies have also explored the role of DL in medical image analysis, showing that these methods improve diagnostic accuracy and operational efficiency 28. The integration of AI into healthcare has the potential to support healthcare professionals and patients across the care continuum, from diagnosis to treatment 80. Although AI shows promise in diagnostic applications, there are still barriers to its integration into clinical treatment processes 81. Incorporating AI into fracture diagnosis could significantly transform the process of diagnosing wrist fractures, increasing the precision and efficiency of detection and treatment.

### 3.2. Reduction in diagnostic errors and missed fractures

The integration of AI into fracture diagnostics has been extensively evaluated, with studies highlighting its role in reducing missed fractures and diagnostic errors. AI-assisted algorithms have demonstrated superior accuracy in detecting fractures on orthopedic X-rays 25. Automated object detection algorithms have been proposed to enhance diagnostic reliability, particularly in high-pressure trauma settings where fractures may be overlooked 82. AI-based systems for hip fracture diagnosis have shown rapid and precise identification capabilities, emphasizing their value in clinical settings 83. Furthermore, AI models have significantly improved wrist trauma diagnostics, reducing reliance on additional diagnostic procedures 36. AI-based models have also been shown to detect commonly missed fractures, such as distal radius fractures, one of the most frequent fracture types 84. Similarly, across various anatomical regions, AI-supported fracture detection has demonstrated high diagnostic accuracy, particularly when combined with traditional methods 85. Overall, the incorporation of AI into fracture diagnosis can substantially decrease diagnostic errors and overlooked fractures, thereby enhancing patient outcomes and optimizing the diagnostic workflow. AI-driven systems improve diagnostic accuracy and efficiency, facilitating timely and precise treatment strategies and contributing to superior patient care.

### 3.3. Reducing unnecessary imaging: AI's contribution to diagnostics

Integrating AI into medical diagnostics has demonstrated substantial potential in enhancing accuracy and efficiency. Within orthopedic practice, AI has been particularly effective in fracture detection, contributing to a reduction in unnecessary imaging studies 25. The application of AI models for detecting fractures in radiographic images facilitates early diagnosis, thereby reducing redundant clinical evaluations 86. This technological advancement streamlines diagnostic workflows and improves patient outcomes. A systematic review and meta-analysis by Wong *et al*. 71 evaluated AI's performance in diagnosing hand and wrist fractures and dislocations. The study demonstrated AI's potential to enhance diagnostic accuracy and reduce unnecessary imaging, reinforcing its value in clinical practice. The integration of AI into clinical practice has been a topic of interest in recent literature 77. Advancements in deep learning for fracture detection, with a focus on scaphoid fractures, have led to improved diagnostic accuracy, potentially decreasing the incidence of unnecessary immobilization 87. Integrating AI in fracture diagnosis enhances diagnostic speed and efficiency, improving patient outcomes. However, AI applications in this field remain constrained by certain limitations that warrant further exploration. For instance, some systematic reviews have excluded studies involving CT imaging, underscoring the necessity for more comprehensive research into AI-based fracture assessment 85. To minimize risks associated with pediatric upper extremity imaging and to facilitate the effective integration of AI into global healthcare systems, further research is essential 88,89. In summary, AI has the potential to significantly enhance fracture diagnosis by reducing redundant imaging, increasing diagnostic accuracy, and improving patient care. Future studies should address technological limitations, optimize AI algorithms for clinical application, and evaluate long-term impacts on orthopedic decision-making.

### 3.4. Expediting diagnosis and reporting

The application of AI in wrist fracture diagnosis represents a promising advancement in medical imaging, facilitating faster and more accurate diagnostic processes. While comprehensive frameworks for human-AI interaction have been established in general radiology, distinguishing integration models such as AI as triage tool and AI as secondary or safety-net reader-direct validation of these specific workflow models in the context of wrist fracture detection remains limited 90. Most wrist fracture AI studies to date have evaluated AI as a concurrent decision support tool or as a second reader in retrospective simulation setting, rather than prospective implementation of triage or safety-net workflows. Future research should specifically assess these distinct integration strategies for wrist imaging to determine which model optimizes diagnostic accuracy, workflow efficiency, and cost-effectiveness in real-world clinical practice.

In the concurrent decision support model evaluated by Lee *et al*., AI assistance was provided to novice radiologists during image interpretation through heatmap visualizations and probability scores. This approach significantly improved diagnostic performance for scaphoid fractures, which are frequently overlooked in standard clinical assessments: the AUC for scaphoid fracture detection increased from 0.75 to 0.85 for one novice radiologist and from 0.71 to 0.80 for another. However, the study did not evaluate AI as an independent triage tool or as a sequential second reader; rather, AI served as a real-time assistive device during the interpretation process. The authors noted that interpretation time increased slightly with AI assistance, highlighting the importance of optimizing workflow integration 3.

Furthermore, the benefits of AI assistance extend beyond diagnostic accuracy improvements to include reductions in unnecessary diagnostic procedures and optimization of clinical decision-making. In a prospective study of emergency department clinical decision-making, Keller *et al*. 36 evaluated physicians using an AI tool for distal radius fracture detection. The AI-supported group was able to make a diagnosis without additional support in 75% of cases, compared to only 52% in the control group without AI assistance. The AI-supported group also required significantly fewer additional CT scans, thereby reducing unnecessary radiation exposure and potential healthcare costs. Notably, physicians using the AI tool reported significantly lower subjective stress levels, suggesting that AI assistance may alleviate cognitive burden in time-sensitive emergency settings. These findings indicate that reliable AI fracture detection models can not only enhance diagnostic confidence but also streamline clinical workflows by reducing reliance on confirmatory imaging and specialist consultations.

From a broader perspective, a systematic review by Kwee *et al*. 91 evaluating the commercially available AI tool BoneView across multiple anatomical regions found that AI assistance significantly improved sensitivity for fracture detection in the majority of readers, although specificity results were mixed. Importantly, reading speed was either unchanged or significantly improved in three out of four studies. This apparent improvement in reading speed contrasts with the slight increase in interpretation time reported by Lee *et al*. 3 for novice radiologists using concurrent AI assistance. This discrepancy may reflect differences in AI interface design, reader experience levels, the specific workflow integration model employed, or the complexity of the fracture types being evaluated. The systematic review also noted substantial heterogeneity across studies and highlighted methodological quality concerns, emphasizing the need for rigorous prospective validation before widespread clinical implementation.

In the specific context of pediatric wrist trauma, Ju *et al*. 14 demonstrated that a YOLOv8-based fracture detection model augmented with contrast and brightness adjustment achieved state-of-the-art mean average precision, outperforming YOLOv7-based models. The authors further developed a user-friendly graphical interface application designed to assist pediatric surgeons in regions with limited access to radiologists, illustrating how AI can democratize fracture diagnostic capabilities across diverse healthcare settings.

Collectively, these studies demonstrate promising tangible benefits of AI in wrist fracture diagnosis, including improved diagnostic accuracy for subtle fractures 3, reduced unnecessary CT scans and physician stress 36, and maintained or improved reading speed in most clinical contexts 91. However, the optimal integration model for routine clinical practice-whether concurrent decision support, second reader, or triage tool-likely remains context-dependent and requires further prospective, multi-institutional validation to establish evidence-based guidelines for AI deployment. Improving AI-driven X-ray interpretation requires larger, higher-quality training datasets and incorporating advanced machine learning approaches, including deep reinforcement learning 25. In a systematic review and meta-analysis conducted, the accuracy of AI models in identifying hand and wrist fractures and dislocations was determined, highlighting the potential of AI in fracture diagnosis 71. A deep learning algorithm was developed and trained for detecting fractures in wrist X-rays, demonstrating high accuracy and sensitivity, as reported in a study on using artificial intelligence for fracture diagnosis in orthopedic X-rays 25. Similarly, integrating AI models into clinical workflows has significantly improved diagnostic processes, as mentioned in *Artificial Intelligence-Based Applications for Bone Fracture Detection* 83. Integrating AI into clinical practice offers significant advantages, including the ability to analyze large datasets efficiently and accurately. The research emphasizes AI's potential to accelerate wrist fracture diagnosis and reporting processes. The incorporation of AI into disease diagnosis and classification, highlighted by 92, can address various challenges and facilitate the smooth integration of AI into medical practice. In wrist fracture diagnosis, AI shows promise in accelerating diagnosis and enhancing the accuracy of reports. The application of cutting-edge machine learning models and the integration of AI systems into clinical workflows could significantly improve the practice of orthopedic X-ray analysis.

### 3.5. Workflow integration and cost-effectiveness

The application of AI in fracture diagnosis has demonstrated promising potential for optimizing resource allocation within healthcare settings 25. Recent economic evaluations provide concrete evidence for the cost-effectiveness of AI-assisted fracture detection. A comprehensive budget impact analysis published in Value in Health evaluated the adoption of a commercially available AI fracture detection application across NHS emergency departments in England 93. Over a one-year horizon, the analysis estimated that among 658,564 radiographs performed for suspected wrist, ankle, or hip fractures, AI integration would reduce the number of patients returning to the ED with a missed fracture by approximately 21,674 cases and reduce unnecessary referrals to fracture clinics by approximately 20,916 cases. The cost of current practice was estimated at £66,646,542, while the AI-integrated scenario reduced costs to £63,012,150, generating a net return on investment of £3,634,392 for the NHS.

Beyond economic considerations, successful integration of AI into clinical workflows requires a structured implementation framework. Shah *et al*. 94 proposed a four-phase framework for responsible AI deployment in radiology: (1) validation-retrospective or offline testing on institutional data to assess local performance; (2) deployment-progressing from limited trial to full deployment with emphasis on workflow integration and stakeholder feedback; (3) value assessment-longitudinal evaluation of financial and non-financial returns on investment; and (4) post-deployment surveillance-monitoring for data drift and maintaining AI safety. Similarly, Trivedi *et al*. 95 described a rubric-based approach for AI model evaluation and deployment at a large academic center, emphasizing holistic assessment beyond performance metrics.

A review on human-AI interaction in radiology outlines several workflow integration models, including AI as a triage tool (pre-screening normal cases), AI as a second reader (safety net for missed findings), and AI as a concurrent support tool (real-time decision aid) 90. The choice of integration model depends on clinical context, AI performance characteristics, and local workflow requirements. Separately, the TRIAGE framework provides a clinically grounded evaluation structure for diagnostic AI testing aligned with real clinical use cases, including screening, triage, second reading, and confirmatory testing 96. In addition to direct cost savings, AI integration offers non-monetary benefits including the potential to reduce physician burnout through workload redistribution, improve patient satisfaction through faster diagnostic turnaround, and enhance training opportunities for residents who can use AI-generated annotations as educational tools. By prioritizing the optimization of workflows and resource distribution, healthcare providers can elevate care quality while achieving measurable cost reductions. By streamlining workflows and improving cost efficiency, AI algorithms can significantly increase operational effectiveness 97. This optimization supports better patient outcomes and personalized treatment planning 98. Moreover, the integration of Internet of Things (IoT) applications, including wearable devices and medical technologies, strengthens the role of technology in patient care 99. The real-time monitoring and data collection enabled by these tools are essential for improving resource management and healthcare delivery. As healthcare systems evolve, the need for efficient resource management grows in importance 88. By utilizing AI and IoT to predict patient flow and streamline inventory management, healthcare systems can ensure better resource allocation and improved patient care 100. By prioritizing the optimization of workflows and resource distribution, healthcare providers can elevate care quality and help shape the future of healthcare.

### 3.6. Improving patient outcomes

Incorporating AI into wrist fracture diagnosis has demonstrated the potential to improve patient outcomes. AI algorithms contribute to more precise treatment decisions, which enhance patient classification and overall results 25. Medical imaging, such as X-rays, is integral to diagnosing and managing fractures. Incorporating AI into this imaging process could significantly elevate patient outcomes in orthopedic care 25. One strategy to enhance AI interpretation of X-ray images is to expand both the size and quality of the datasets used for training while also adopting more advanced machine learning methods, including deep reinforcement learning 25. AI integration in medical imaging can streamline clinical workflows, reduce diagnostic errors, and raise the standard of medical care, leading to better patient outcomes 101. Studies have shown that incorporating AI into clinical practice can enhance medical care and improve health outcomes for patients with conditions such as burns, wounds, and diabetic retinopathy 77,98. By incorporating AI along with pharmacogenomic data, healthcare providers can achieve better clinical results and provide more effective patient care 92. The overall integration of AI into wrist fracture diagnosis holds the potential to revolutionize clinical care, ensuring accurate diagnoses, timely treatment decisions, and improved patient outcomes and quality of care 102.

## 4. Challenges

The incorporation of AI into the process of diagnosing fractures through orthopedic X-rays introduces significant ethical and legal challenges that require thorough consideration 25. A critical concern raised in various studies is the potential influence of AI on the emotional experiences of patients 25. In the healthcare setting, the application of AI raises four primary ethical issues, including the necessity for informed decision-making, that must be resolved to achieve its full potential 103. The implementation of large language models in healthcare raises significant privacy and security risks, which must be managed thoughtfully to prevent harm to patients and avoid legal implications 104. The emergence of mental health chatbots has brought critical ethical challenges, as these tools incorporate varying levels of artificial intelligence 105. The broader integration of AI into healthcare raises fundamental ethical questions, particularly regarding fairness and transparency 106. The potential biases inherent in AI models require careful evaluation, emphasizing the importance of addressing ethical concerns and biases in AI and machine learning systems 107. As AI continues to reshape healthcare, the accompanying ethical, legal, and human resource challenges are becoming more prominent 108. The increase in research on AI ethics reflects a growing awareness of the ethical issues surrounding AI adoption in healthcare 109. Consequently, the application of AI in healthcare, including in fracture diagnosis, must be approached with a detailed assessment of both ethical and legal implications to guarantee responsible and effective use.

The integration of artificial intelligence into clinical practice raises concerns about transparency, accountability, and potential bias within algorithms 77. Within the field of radiology, a critical ethical issue is whether AI can achieve diagnostic accuracy equivalent to that of human professionals in identifying fractures 110. A systematic review of AI applications in orthopedic conditions highlights the necessity for transparent AI algorithms to promote ethical standards in healthcare 111. Moreover, various initiatives led by government bodies are evaluating how federal agencies can effectively implement AI technologies 112. AI and machine learning research in healthcare further emphasizes the importance of ensuring transparency, replicability, and ethical considerations in the development and adoption of these algorithms 113. A primary ethical concern in AI adoption is the need for transparent and interpretable algorithms, particularly in applications such as wrist fracture diagnosis 25.

Integrating AI into medical imaging, with a particular focus on fracture diagnosis, has led to significant ethical challenges, primarily surrounding patient privacy and data protection. The literature emphasizes the transparency and explainability of AI algorithms as critical factors for the successful adoption of AI in medical settings 25. However, patient data privacy and security concerns are significant limiting factors in applying AI technology within healthcare systems 92. As the demand for medical imaging grows, radiologists face increased pressure, positioning AI as a valuable tool for assisting in diagnosis 110. However, protecting patient privacy and concerns over liability associated with AI usage remain critical challenges in the field 108. AI has demonstrated promising potential in improving diagnostic accuracy in orthopedic disease diagnosis, particularly in studies involving patients with wrist fractures 111. However, ensuring patient privacy and securing data remain essential, necessitating robust ethical guidelines and legal frameworks to govern AI usage 77. Concerns regarding data security and privacy in healthcare settings have been highlighted in the literature, emphasizing the need for stringent safeguards to protect sensitive medical data 88,99. Addressing these ethical concerns as AI advances will be crucial for the responsible integration of AI technologies in patient care.

Beyond the general ethical and regulatory challenges discussed above, several barriers specifically impede the clinical deployment of AI for wrist fracture detection. Integration with existing PACS and RIS systems remains technically challenging, as many AI solutions require custom middleware or cloud-based processing that may not comply with institutional data governance policies 90. Workflow disruption during the transition period can temporarily reduce radiologist efficiency, necessitating careful change management and parallel testing phases 94. Algorithm performance drift over time—due to changes in imaging equipment, patient demographics, or referral patterns—requires ongoing post-deployment surveillance and model retraining protocols 94,96. Liability and accountability questions remain unresolved: under current medical malpractice frameworks, there is no transfer of liability to AI systems, and the allocation of legal responsibility for errors arising from joint human-AI decisions remains unclear 90. Automation bias, the tendency to over-rely on AI recommendations, and algorithmic aversion, excessive distrust of AI outputs, represent cognitive risks that require explicit mitigation strategies, especially for trainees. Addressing these implementation-specific barriers will be essential for successful and sustainable AI adoption in orthopedic radiology.

AI fracture detection software is regulated as Software as a Medical Device (SaMD). In the U.S., the FDA has authorized 950 AI/ML-enabled medical devices as of June 2024, with 97% cleared via the 510(k) pathway 114. The FDA has issued two complementary guidances for AI-enabled medical devices in 2025. A draft guidance on Artificial Intelligence-Enabled Device Software Functions: Lifecycle Management and Marketing Submission Recommendations emphasizes a total product lifecycle approach, covering design, development, post-market monitoring, transparency, and bias mitigation throughout the device's lifecycle. A final guidance on Marketing Submission Recommendations for a Predetermined Change Control Plan for Artificial Intelligence-Enabled Device Software Functions establishes Predetermined Change Control Plans, which allow manufacturers to pre-specify algorithm updates—including modifications to improve sensitivity or specificity—that can be implemented without additional marketing submissions once authorized by the FDA as part of an initial 510(k), De Novo, or PMA application. These frameworks support iterative improvement of AI fracture detection software while maintaining regulatory oversight (Supplemental file 2). In the EU, AI-enabled medical devices must comply with both the Medical Device Regulation (MDR 2017/745) and the AI Act (2024/1689), creating overlapping requirements for conformity assessment, risk classification, clinical evaluation, and post-market surveillance. The AI Act envisages that there shall be no duplications, and notified bodies competent to assess medical devices under the MDR may also be designated under the AI Act to enable joint CE marking conformity assessment. However, the article notes that such combined conformity assessment may not always be straightforward, as there is no unified European regulatory institute to harmonize implementation across member states 115. In the UK, NICE's early value assessment conditionally recommends four AI fracture detection technologies—BoneView, RBfracture, Rayvolve, and TechCare Alert—for use in NHS urgent care settings during a 2-year evidence generation period, subject to ongoing data collection and regulatory approval including NHS England's Digital Technology Assessment Criteria (DTAC) (Supplemental file 1). Manufacturers should align their development and quality management processes with internationally recognized standards. ISO 13485:2016 establishes requirements for medical device quality management systems and is recognized by the FDA, MHRA, and IMDRF. ISO 14971:2019 provides the framework for risk management throughout the device lifecycle, which IMDRF's software risk characterization guidance explicitly references. Additionally, IEC 62304 defines the software lifecycle processes required for medical device software development and maintenance, and is currently being revised to incorporate AI-specific requirements for AI-enabled medical devices (https://pureclinical.eu/news/mhra-imdrfs-latest-guidance-on-ai-and-medical-device-software;https://www.itu.int/en/ITU-T/studygroups/2025-2028/21/Pages/rm/dhealth.aspx). Understanding these pathways facilitates smoother market access and responsible clinical adoption.

While the diagnostic performance metrics reported in Section 3 demonstrate the potential of AI for wrist fracture detection, the generalizability of these findings across diverse populations and clinical settings remains a critical concern. A systematic review by Oliveira e Carmo *et al*. examined 36 studies developing CNNs for fracture detection and found that only four reported any form of external validation 116. This scarcity of external validation greatly limits the potential for transferring these models from their development institutions to other hospitals with comparable diagnostic performance 116.

The impact of cross-population domain shift-distributional differences arising from demographic variability, variations in imaging protocols, scanner hardware, and differences in disease prevalence, can degrade model performance by 10-25% when models are tested on unseen populations 117. For wrist fracture detection specifically, Raisuddin *et al*. reported that a deep learning model achieving near-perfect performance (AUROC 0.99) on a general population test set dropped substantially to an AUROC of 0.84 on a challenging test set comprising cases requiring CT confirmation, highlighting the vulnerability of AI models to shifts in case mix and disease severity 10.

Most AI fracture detection studies have been developed using datasets from single centers or geographically limited populations, raising concerns about their applicability to broader patient groups. A cross-population validation study by Ruitenbeek *et al*. evaluated an AI tool trained on over 1.5 million radiographs from Indian hospitals on a Dutch cohort of 14,311 radiographs, finding that classification performance remained robust, but fracture localization accuracy varied substantially across anatomical regions, from 90% for clavicle fractures to only 7% for rib fractures 118. This demonstrates that while classification performance may generalize across populations, localization accuracy-critical for surgical planning-remains highly context-dependent and requires careful validation in target clinical settings.

Wrist fracture detection in older adults poses particular challenges, as age-related bone density loss, osteopenia, and osteoporosis alter radiographic bone texture, potentially affecting AI model performance. Although analogous systematic studies for wrist fracture AI are lacking, the principle that AI models can underperform in demographic subgroups underrepresented in training data is well established in radiology 110. Given that fracture patterns and radiographic characteristics differ substantially between children and adults, wrist fracture AI models trained predominantly on adult or mixed-age populations may not generalize reliably to pediatric or geriatric populations without dedicated validation. For example, both Zech *et al*. and Ju *et al*. developed pediatric specific models trained exclusively on pediatric datasets, highlighting the importance of population matched training data 14,23.

To address these generalizability concerns, several strategies are recommended. Prospective external validation on geographically and demographically diverse, consecutively enrolled patient cohorts is essential before clinical deployment. Subgroup performance analyses by age, sex, and ethnicity should be routinely reported to identify potential biases. The use of reporting guidelines such as CONSORT-AI, SPIRIT-AI, and TRIPOD-ML can improve methodological rigor and facilitate critical appraisal of model performance 116. Ongoing real-world surveillance, as recommended by Shah *et al*., should monitor for performance drift due to changes in patient demographics, imaging equipment, or referral patterns 94. Trivedi *et al*. similarly advocate for continuous post deployment monitoring to ensure AI models remain safe and effective in real-world clinical settings 95. While AI shows substantial promise for wrist fracture detection, current evidence is limited by a lack of rigorous external validation and insufficient evaluation across diverse populations. Researchers and clinicians should interpret reported performance metrics with caution, prioritize external validation on local, representative data before adoption, and actively work to ensure that training datasets reflect the demographic diversity of target patient populations.

## 5. Future prospects of AI in wrist injury diagnosis and treatment

The application of AI in the field of wrist injuries offers substantial potential for enhancing diagnostic accuracy, treatment planning, and patient outcomes. Various studies have emphasized the role of AI technologies across various aspects of wrist injury care, with a particular focus on fracture detection and rehabilitation. A significant advancement in this field is the development of AI algorithms for detecting wrist fractures in radiographs. Research suggests that AI models surpass non-specialized radiologists in identifying wrist fractures, with an observed sensitivity of 83%, compared to 76% for initial radiology reports made by non-specialized radiologists. When AI is used in conjunction with radiologist interpretation, sensitivity increases to 88%, although specificity slightly decreases to 92% 119. This indicates that AI integration into clinical practice could enable more accurate and timely diagnoses, potentially reducing missed fractures, especially in complex cases such as scaphoid fractures, which are challenging to detect 3,119. Research has explored the feasibility of using advanced imaging modalities, such as 3DUS coupled with AI, for evaluating pediatric wrist fractures. A study involving children with wrist injuries demonstrated that AI-based interpretation of 3DUS scans achieved a sensitivity of 100%, suggesting its effectiveness in ruling out fractures before traditional radiographic imaging 5. This approach could significantly decrease unnecessary radiation exposure in pediatric populations, highlighting AI's potential to enhance patient safety while preserving diagnostic precision. In addition to fracture detection, AI is being leveraged to create predictive models for scaphoid fractures based on clinical factors such as pain location, age, and injury mechanism. Machine learning models trained on these parameters can assist clinicians in determining when advanced imaging is needed, thereby reducing unnecessary imaging procedures while ensuring appropriate care for high-risk patients 120. The application of AI in rehabilitation medicine represents a promising field of development. Machine learning and AI tools are being used to develop individualized treatment protocols for patients with motor impairments, including those recovering from wrist injuries. By analyzing data collected from wearable sensors and robotic assistive devices, AI can guide adaptive neurorehabilitation approaches customized to each patient's needs 121. This can potentially improve rehabilitation outcomes by optimizing recovery processes and enhancing patient satisfaction. Furthermore, developing XAI methods, such as Grad-CAM, enhances the interpretability of AI models in clinical settings. These methods allow clinicians to visually interpret the areas of an image that the AI model prioritizes when making diagnostic assessments, thereby strengthening the reliability of AI-driven clinical decisions 40. As AI technologies progress, their transparency and reliability will be critical to their integration into clinical practice. However, challenges persist in the broad adoption of AI for managing wrist injuries. Robust validation of AI models across diverse clinical settings is necessary to ensure both their efficacy and safety. Moreover, healthcare professionals need proper training in interpreting AI-generated insights and seamlessly integrating them into routine clinical practice 121,122.

## 6. Conclusion

In conclusion, the potential of artificial intelligence in managing wrist injuries is vast, with significant advantages in areas such as fracture detection, treatment planning, and rehabilitation. Ongoing advancements and research are expected to produce more refined AI tools to improve patient care and treatment outcomes significantly. As these technologies evolve, they could become a foundational component of orthopedic practice, reshaping the management of wrist injuries.

## Supplementary Material

Supplementary file 1.

Supplementary file 2.

## Figures and Tables

**Table 1:** The application of artificial intelligence technology in wrist joint injuries

Year	Artificial Intelligence Techniques	Application Scenarios	Achievements/Contributions	Refs.
2024	DL, CNN	Detection of wrist fractures from plain wrist radiographs, comparing DL algorithms to healthcare experts' consensus	Conducted a systematic review and meta-analysis of DL algorithms for detecting wrist fractures. Found that CNNs achieved a summary sensitivity of 92% (95% CI: 80%-97%) and a summary specificity of 93% (95% CI: 76%-98%) compared to healthcare experts. Highlighted the need for robust reference standards, external dataset validation, and investigation of diagnostic performance of healthcare experts aided by CNNs.	9
2021	DL, CNN, Grad-CAM	Detection of wrist fractures (distal radius fractures) from X-ray images, comparing DL models to human experts on general and challenging cases.	Developed an open-source DL pipeline called DeepWrist for wrist fracture detection. Evaluated the pipeline on two test sets: a general population test set and a challenging test set requiring CT confirmation. While DeepWrist achieved near-perfect performance on the general test set (AUROC of 0.99), its performance dropped significantly on the challenging test set (AUROC of 0.84). Highlighted the limitations of current DL models in handling difficult cases and emphasized the need for robustness and safety assessments before clinical deployment. Investigated the use of Deep Ensembles to detect out-of-distribution cases but found limited success in distinguishing between general and challenging cases.	10
2021	DCNN	Automated Detection and Classification of Distal Radius Fractures.	Evaluated the performance of a DCNN for detecting and classifying distal radius fractures, metal, and cast on radiographs using labels from radiology reports. The models achieved high AUC values for detecting metal (0.99) and cast (1.00). Fracture detection performance approximated that of radiology residents (AUC 0.98). Demonstrated a positive correlation between training set size and model performance in most categories. Highlighted the potential of using low-effort data labeling from radiology reports for training AI models.	11
2019	CNN	Automated Detection and Localization of Fractures in Wrist Radiographs. Emergency Department Radiographs, Assisting Emergency Medicine Clinicians.	Demonstrated the feasibility of using an object detection CNN to detect and localize fractures on wrist radiographs with high sensitivity and specificity. Showed per-fracture sensitivity of 91.2% (frontal view) and 96.3% (lateral view). Per-image sensitivity of 95.7% (frontal view) and 96.7% (lateral view), with AUC values of 0.918 and 0.933, respectively. Per-study sensitivity of 98.1% and AUC of 0.895. Lower sensitivity for minimally displaced fractures compared to displaced fractures. Provided location information of fractures, enhancing trust and verifiability for clinicians.	12
2021	CNN	Automated Diagnosis of Distal Radius Fractures. Use of Biplane Plain X-rays	Developed an AI system capable of diagnosing distal radius fractures with high accuracy using relatively small datasets. Achieved diagnostic accuracy of 98.0% ± 1.6% for distal radius fractures and 91.1% ± 2.5% for ulnar styloid fractures. Obtained AUC values of 0.991 for distal radius fractures and 0.956 for ulnar styloid fractures. Demonstrated the effectiveness of using a pre-trained VGG16 model and image augmentation to enhance diagnostic performance with limited data. Proposed a two-stage diagnostic approach using both anteroposterior and lateral X-ray images to improve accuracy.	1
2018	DCNN	Automated Detection and Localization of Fractures in Radiographs. Emergency Department Radiographs. Assisting Emergency Medicine Clinicians	Developed a deep learning model to detect and localize fractures in radiographs with high accuracy. Trained the model using annotations from 18 senior subspecialized orthopedic surgeons on 135,409 radiographs. Significant improvements in diagnostic accuracy when clinicians used the model's assistance: Sensitivity increased from 80.8% to 91.5%. Specificity increased from 87.5% to 93.9%. Relative reduction in misinterpretation rate by 47.0%. The model's performance is comparable to that of senior orthopedic surgeons.	13
2023	DL, YOLOv8 algorithm	Fracture detection in pediatric wrist trauma X-ray images. Development of a GUI application ("Fracture Detection Using YOLOv8 App") to assist pediatric surgeons in analyzing X-ray images.	Achieved state-of-the-art mean average precision of 0.638 on the GRAZPEDWRI-DX dataset, outperforming YOLOv7 and other models. Improved detection accuracy for bone anomalies through data augmentation techniques. Developed a user-friendly application to assist pediatric surgeons in interpreting X-ray images, reducing the probability of misclassification. Provided a practical solution for hospitals with limited access to radiologists, especially in underdeveloped regions.	14
2020	DL, CNN	Detection and localization of distal radius fractures in wrist radiographs.	Achieved high performance comparable to radiologists using a relatively small dataset (AUC up to 0.96 on internal test set, sensitivity up to 0.90, specificity up to 1.0). Demonstrated the ability to localize fractures with over 90% alignment with radiologists' annotations Highlighted the potential of using deep learning systems as a computer-aided diagnostic tool in clinical workflows.	15
2021	CNN, Grad-CAM	Detection of wrist fractures in the emergency room using plain radiographs. Assisting emergency physicians in diagnosing fractures quickly and accurately.	Demonstrated that DenseNet-161 and ResNet-152 models achieved high performance in detecting wrist fractures with AUROCs of 0.962 and 0.947, respectively. Sensitivity, specificity, positive predictive value, negative predictive value, and accuracy for both models. The importance of using Grad-CAM to visualize and interpret fracture detection regions. Developed a model representative of real-world emergency room settings, addressing limitations of previous heterogeneous datasets. Provided insights into the potential use of AI models in clinical practice, especially in scenarios where radiologists are unavailable	16
2022	CNN, TL	Detection of hand fractures from plain radiographs in emergency departments. Assisting physicians in interpreting hand radiographs, especially in small hospitals or during night shifts/weekends	Developed a Computer-Aided Diagnosis method using deep learning to assist in the diagnosis of hand fractures. The effectiveness of transfer learning and data augmentation in improving model performance with limited data. Provided a potential solution for emergency departments where radiologists or orthopedic specialists may not be readily available.	17
2022	CNN, TL	DRF from wrist radiographs. Assisting hand orthopedic surgeons in interpreting radiographs. Improving diagnostic accuracy in emergency or outpatient settings	Achieved high diagnostic accuracy (99.3%) for DRF detection using combined AP and lateral views. Matched or exceeded the diagnostic performance of three experienced hand orthopedic surgeons. Demonstrated the effectiveness of using multiple views in improving diagnostic capability. Highlighted the potential of CNNs in fracture detection with limited training data through fine-tuning and data augmentation. Provided a practical solution for improving fracture detection in clinical settings, especially for non-specialists	18
2024	GAN	Reducing cast shadow artifacts in wrist radiographs. Improving diagnostic confidence and efficiency for radiologists. Potential for reducing patient radiation doses and improving clinical workflows.	Extended CycleGAN model with a perceptual loss function and self-attention layer to improve cast shadow suppression. The enhanced CycleGAN model produced high-quality cast-suppressed images comparable to original images. Radiologists could not distinguish between AI-enhanced and unenhanced images, indicating high realism of generated images. Improved diagnostic confidence in radiologists, leading to more decisive reports in some cases. Validated the model's clinical potential by comparing AI-enhanced image reports with follow-up imaging results.	19
2022	DL, CNN	Fracture detection in wrist X-ray images. Supporting physicians in diagnosing fractures, particularly in emergency services. Potential for real-time applications on portable devices.	Developed a unique ensemble model that achieved the highest average precision (AP50) of 0.8639 for fracture detection in wrist X-ray images. Identified and applied the most effective data augmentation methods for improving detection performance. First use of LRP metrics for evaluating fracture detection in biomedical images. Improved detection results by approximately 10% compared to individual models using ensemble techniques.	20
2017	CNN, TL	Automated fracture detection on plain radiographs. Improving radiology workflow efficiency and reducing diagnostic delays	Demonstrated the feasibility of using transfer learning from pre-trained CNNs for fracture detection on plain radiographs. Achieved an AUC score of 0.954, with sensitivity of 0.9 and specificity of 0.88. Provided proof of concept for using transfer learning in medical imaging with a moderate sample size. Contributed to the growing body of literature on AI applications in radiology, particularly in the context of fracture detection	21
2022	DL, CNN, GAN	Fracture Detection in Hand X-rays. Assisting Radiologists and Surgeons in Diagnosis. Improving Detection Accuracy and Workflow Efficiency.	Development of a Custom Dataset. 4346 hand X-rays (anteroposterior, lateral, and oblique views) with a significant number of hairline fractures. An attention mechanism-based GAN for automatic windowing enhancement, achieving 93% SSIM with manual windowing. A novel detection network with a feature extraction module (ResNeXt-TA) and a detection module (Soft-NMS) for improved accuracy. AP improvement of up to 7% compared to mainstream frameworks. Automated preprocessing and detection reduce the need for manual parameter tuning and increase clinical applicability.	22
2023	DL, CNN	Detecting scaphoid fractures in conventional multi-view hand and wrist radiographs. Assisting musculoskeletal radiologists in reducing misdiagnosis rates and improving diagnostic efficiency.	Diagnostic Performance: The AI algorithm achieved an AUC of 0.88, comparable to five experienced musculoskeletal radiologists (average AUC of 0.87). Diagnostic Efficiency: AI assistance significantly reduced radiologists' reading time (average reduction of 49.4%) and improved specificity and PPV for some radiologists. Value of Multi-views: Demonstrated that oblique and ulnar-deviated views enhance diagnostic performance compared to single PA views in AI detection. Clinical Potential: Preliminary evidence suggests that AI assistance can improve diagnostic efficiency but has limited impact on diagnostic accuracy.	8
2023	DL, CNN	Medical Imaging: Detecting pediatric wrist fractures, including subtle buckle fractures of the distal radius. Enhancing the diagnostic accuracy of trainee radiologists by providing AI-generated bounding boxes for suspected fractures.	Model Performance: Achieved an AUC of 0.92, accuracy of 88%, sensitivity of 88%, and specificity of 89% in identifying fractures. Identified 90% of buckle fractures with a sensitivity of 88% and specificity of 89%. Improved Resident Accuracy: Access to AI predictions increased resident accuracy from 80% to 93% for detecting any fracture (P < 0.001) and from 69% to 92% for detecting buckle fractures (P < 0.001). Human-AI Collaboration: Residents outperformed AI in cases of disagreement after accessing AI predictions (73% vs. 27%, P = 0.002).	23
2022	DL, TL	Fracture detection in wrist X-ray images to support physicians in diagnosing fractures.	Developed 20 different fracture detection procedures using various deep learning models. Achieved the highest average precision of 0.8639 with the WFD-C ensemble model. Used LRP as an evaluation metric for the first time in biomedical fracture detection. Demonstrated the potential for real-time fracture detection in clinical settings.	20
2022	DL, CNN, TL	Fracture Detection and Segmentation in Wrist X-rays. Automated Identification of Fractures in Resource-Limited Settings	Development of a deep neural network for detecting, localizing, and segmenting wrist fractures in X-ray images. Utilization of transfer learning with a surface crack dataset to improve model convergence. Modification of the backbone network by replacing the max-pool layer with Adaptive Concat Pool, Adaptive Avg Pool, and Adaptive Max Pool layers. Implementation of a two-phase training approach: inductive transfer learning (surface crack dataset) and transductive transfer learning (wrist fracture dataset). Achievement of an average precision of 92.278% for fracture detection and 79.003% (AP75), and 77.445% for fracture segmentation (AP50) and 52.156% (AP75).	24

**Table 2:** Diagnostic performance of AI models for wrist fracture detection

Fracture type / Reader	Pooled sensitivity (95% CI)	Pooled specificity (95% CI)	AUC / LR	Refs.
Distal radius (AI)	92% (88-95)	89% (84-92)	—	69
Distal radius (human)	95% (91-97)	94% (91-96)	—	69
Scaphoid (AI)	85% (73-92)	83% (76-89)	—	69
Scaphoid (human)	71% (66-76)	93% (90-95)	—	69
CNNs (overall wrist)	92% (80-97)	93% (76-98)	—	9
Hand/wrist (overall)	—	—	AUC 0.946; LR+ 7.69 (6.40-9.19)	71

## Data Availability

The datasets generated and analysed during this study are available from the corresponding author on reasonable request.

## Abbreviations

AI: artificial intelligence
AP: average precision
AUC: area under the curve
CNN: convolutional neural networks
CT: computed tomography
DCNN: deep convolutional neural networks
DL: deep learning
DRF: detection of distal radius fractures
GAN: generative adversarial network
Grad-CAM: gradient-weighted class activation mapping
IOT: internet of things
LRP: localization recall precision
LSTM: long short-term memory
Map: mean average precision
MRI: magnetic resonance imaging
MSK: musculoskeletal
NLP: natural language processing
SaMD: software as a medical device
SL: scapholunate ligament
SSIM: structural similarity index
SVMs: support vector machines
TL: transfer learning
WJI: wrist joint injuries
XAI: explainable artificial intelligence
3DUS: three-dimensional ultrasound
