# Supplementary material for: Advances in Artificial Intelligence for Wrist Joint Injury Diagnosis
Source: Int J Med Sci. 2026 Jul 1;23(8):2548–67. doi: 10.7150/ijms.134301 (PMC13411213; doi:10.7150/ijms.134301)
Supplement: Supplementary file 1 [file ijmsv23p2548s1.pdf]

Artificial intelligence (AI) technologies to help detect fractures on X-rays in urgent care: early value assessment

Health technology evaluation
Published: 14 January 2025

www.nice.org.uk/guidance/hte20

© NICE 2025. All rights reserved. Subject to Notice of rights (https://www.nice.org.uk/terms-and-conditions#notice-of-rights).

Your responsibility

This guidance represents the view of NICE, arrived at after careful consideration of the evidence available. When exercising their judgement, healthcare professionals are expected to take this guidance fully into account, and specifically any special arrangements relating to the introduction of new interventional procedures. The guidance does not override the individual responsibility of healthcare professionals to make decisions appropriate to the circumstances of the individual patient, in consultation with the patient and/or guardian or carer.

All problems (adverse events) related to a medicine or medical device used for treatment or in a procedure should be reported to the Medicines and Healthcare products Regulatory Agency using the Yellow Card Scheme.

Commissioners and/or providers have a responsibility to implement the guidance, in their local context, in light of their duties to have due regard to the need to eliminate unlawful discrimination, advance equality of opportunity, and foster good relations. Nothing in this guidance should be interpreted in a way that would be inconsistent with compliance with those duties. Providers should ensure that governance structures are in place to review, authorise and monitor the introduction of new devices and procedures.

Commissioners and providers have a responsibility to promote an environmentally sustainable health and care system and should assess and reduce the environmental impact of implementing NICE recommendations wherever possible.

© NICE 2025. All rights reserved. Subject to Notice of rights (https://www.nice.org.uk/terms-and-conditions#notice-of-rights).

Contents

1 Recommendations 4

Can be used while more evidence is generated 4

Can only be used in research 5

What evidence generation and research is needed 5

2 The technologies 8

Clinical need and practice 8

The interventions 9

The comparator 10

3 Committee discussion 11

Patient and carer considerations 11

Clinical effectiveness 12

System impact 14

Cost effectiveness 15

Research considerations 19

Equality considerations 20

Evidence gap review 21

4 Committee members and NICE project team 24

Committee members 24

NICE project team 25

© NICE 2025. All rights reserved. Subject to Notice of rights (https://www.nice.org.uk/terms-and-conditions#notice-of-rights).

1 Recommendations

Can be used while more evidence is generated

1.1 Four artificial intelligence (AI) technologies can be used in the NHS during the evidence generation period as options to help healthcare professionals detect fractures on X-rays in urgent care. The technologies are:

- for people of any age:
  - Rayvolve
  - TechCare Alert
- for people 2 years and over:
  - BoneView
  - RBfracture.

These technologies can only be used:

- if the evidence outlined in the evidence generation plan is being generated
- once they have appropriate regulatory approval including NHS England's Digital Technology Assessment Criteria (DTAC) approval.

1.2 The companies must confirm that agreements are in place to generate the evidence (as outlined in NICE's evidence generation plan). They should contact NICE annually to confirm that evidence is being generated and analysed as planned. NICE may withdraw the guidance if these conditions are not met.

1.3 At the end of the evidence generation period (2 years), the companies should submit the evidence to NICE in a form that can be used for decision making. NICE will review the evidence and assess if the technologies can be routinely adopted in the NHS.

© NICE 2025. All rights reserved. Subject to Notice of rights (https://www.nice.org.uk/terms-and-conditions#notice-of-rights).

Can only be used in research

1.4 More research is needed on qMSK to help healthcare professionals detect fractures on X-rays of adults in urgent care before it can be used in the NHS.

1.5 Access to qMSK should be through company, research or non-core NHS funding, and clinical or financial risks should be appropriately managed.

What evidence generation and research is needed

1.6 Evidence generation and more research is needed on:

- the diagnostic accuracy of fracture detection in urgent care by healthcare professionals with and without the help of AI technologies
- costs and clinical outcomes associated with different fracture types and missed fractures
- fracture clinic referral rates with and without the help of AI technologies
- any clinically significant changes in treatment decisions for fractures detected with and without the help of AI technologies
- AI software failure rates and reasons for failure
- detection of or failure to detect clinically significant non-fracture-related conditions by healthcare professionals with and without the help of AI technologies
- the diagnostic accuracy of AI technologies to help healthcare professionals detect fractures in different populations
- implementation costs of AI technologies in different urgent care centres.

The evidence generation plan gives further information on the prioritised evidence gaps and outcomes, ongoing studies and potential real-world data sources. It includes how the evidence gaps could be resolved through real-world evidence studies.

© NICE 2025. All rights reserved. Subject to Notice of rights (https://www.nice.org.uk/terms-and-conditions#notice-of-rights).

Potential benefits of use in the NHS with evidence generation

- **Clinical benefit:** Clinical evidence suggests that the AI technologies may improve fracture detection on X-rays in urgent care without increasing the risk of incorrect diagnoses. This could help reduce the number of fractures that are missed in urgent care, which would reduce the risk of further injury or harm to people during the time between the initial interpretation and treatment decision in urgent care and the definitive radiology report.
- **System benefit:** AI technologies may help reduce variation in standard care by providing a consistent baseline for X-ray interpretation unaffected by differences in staff experience or resources between centres. AI technologies would also be unaffected by factors such as staff fatigue, distractions, or working outside normal hours. In centres or at times where definitive radiology reports are available before people are discharged (hot reporting), the benefits of AI assistance may be lower.
- **Resources:** Reducing the number of fractures that are missed at initial interpretation would also reduce the number of people that reattend urgent care after discharge or are recalled to hospital after radiology review. Early results from the exploratory economic modelling show that the AI technologies could be cost effective.
- **Equality:** AI technologies have the potential to reduce geographical inequalities in X-ray interpretation and fracture detection, because they may improve fracture detection in smaller centres with fewer and less-experienced staff. It could also reduce inequalities in provision of service, because there may be improvement in service outside normal hours.

Managing the risk of use in the NHS with evidence generation

- **Clinical risk:** Using AI technologies to help detect fractures on X-rays in urgent care is considered to have a low clinical risk. This is because they are used in addition to standard care in which healthcare professionals make treatment decisions. Additionally, AI technologies do not replace the definitive radiology review. The available evidence suggests that the AI technologies may improve the accuracy of fracture detection.

© NICE 2025. All rights reserved. Subject to Notice of rights (https://www.nice.org.uk/terms-and-conditions#notice-of-rights).

- **Implementation guidance:** Clear local protocols will need to be in place when using AI technologies. This is to ensure that healthcare professionals are confident about what action to take when there is disagreement between the healthcare professional and AI technology.
- **Costs:** There is uncertainty around the cost of some software and the true cost of implementation and ongoing post-market surveillance. Costs were estimated at £1 per scan in the exploratory economic modelling. Centres implementing AI to help fracture detection should ensure the cost per scan is similar to the estimated cost. This guidance will be reviewed after the evidence generation period and the recommendations may change. Centres should take this into account when negotiating contract durations and licence costs.
- **Impact on workforce:** If using AI technologies to help fracture detection becomes more widespread and part of the standard diagnostic pathway, there is a risk of over-reliance on the technologies. This could potentially lead to deskilling of the healthcare professionals who interpret the X-rays. It may also reduce the level of scrutiny for non-fracture-related conditions. This risk could be mitigated if healthcare professionals interpret X-rays before viewing the AI results.
- **Resources:** There is a low risk that using AI technologies to help detect fractures on X-rays may increase fracture clinic referrals and requests for further imaging such as CT or MRI. This should be monitored during evidence generation to inform local fracture detection protocols.
- **Limitations of AI for subgroups:** The AI technologies may not be suitable for use in certain groups, for example, children and young people or people with conditions that affect bone health. Centres should ensure that the AI technologies are used within their indications and any limitations are acknowledged and clearly explained to patients.
- **Equality:** There is a risk that the AI technologies may have reduced diagnostic accuracy in different populations, such as people from ethnic minority backgrounds or people with low socioeconomic status. Healthcare professionals should take this into account when interpreting X-rays of people in these groups.

© NICE 2025. All rights reserved. Subject to Notice of rights (https://www.nice.org.uk/terms-and-conditions#notice-of-rights).

2 The technologies

Clinical need and practice

2.1 Fracture assessment and diagnosis in urgent care typically involves triage in which a nurse, advanced clinical practitioner or doctor will do an initial assessment before requesting imaging. X-rays taken by a diagnostic radiographer are usually the first-line imaging approach for non-complex fractures. X-rays are initially interpreted by a healthcare professional in the urgent care centre, who may be assisted by a preliminary clinical evaluation from the diagnostic radiographer. Multiple surgical and non-surgical treatment options are available depending on the type of fracture.

2.2 NICE's guideline on non-complex fractures recommends that a radiologist, radiographer or other trained reporter should review X-rays and provide a definitive report before the injured person is discharged (hot reporting). Clinical experts explained that in practice this is not always possible and reporting delays can occur ranging from days to weeks.

2.3 Missed fractures are reported to be the most common diagnostic error in the emergency department. Hussain et al. (2019) found that 44% of diagnostic errors in fractures resulted from inappropriate response to imaging. Missed or delayed diagnosis of fractures on radiographs is reported to occur in around 3% to 10% of cases (Kuo et al. 2022).

2.4 Artificial intelligence (AI) technologies that can help healthcare professionals detect fractures on X-ray images could improve the accuracy of fracture diagnoses in urgent care. This could help reduce the:

- number of fractures that are missed before a radiologist or reporting radiographer reviews the X-rays
- number of people being recalled to hospital after radiology review
- risk of further injury or harm to people during the time between the initial interpretation and treatment decision in urgent care and the definitive

© NICE 2025. All rights reserved. Subject to Notice of rights (https://www.nice.org.uk/terms-and-conditions#notice-of-rights).

radiology report

- number of unnecessary referrals to fracture clinics.

  AI technologies may also improve consistency of X-ray interpretation when the ability of healthcare professionals to interpret X-rays is reduced, for example, when they are tired, distracted or working outside normal hours.

The interventions

2.5 The technologies included in this early value assessment are standalone software that use AI-derived algorithms to analyse X-ray images to detect fractures. They are intended to be used as decision aids for healthcare professionals interpreting the X-ray image. Some companies provide the software directly, whereas others provide it through multivendor platforms. The technologies use X-ray radiographs in digital imaging and communications in medicine (DICOM) format, which are stored on the hospital's picture archiving and communications system (PACS). Images are then interpreted using proprietary AI-derived algorithms. The technologies included in this assessment are shown in table 1.

Table 1 Interventions

AI technology (manufacturer)	CE marking	Regions covered	Population	Other pathologies detected
BoneView (Gleamer)	Class IIa	Appendicular skeleton, ribs and thoracic-lumbar spine	2 years and over	Dislocation, effusion, bone lesion
qMSK (Qure.ai)	Class IIb	Appendicular skeleton and ribs	Adults	–
Rayvolve (AZmed)	Class IIa	Appendicular skeleton and ribs	No age limit	Dislocation, joint effusion, and chest pathologies (pneumothorax, cardiomegaly, pleural effusion, pulmonary oedema, consolidation, nodule)

© NICE 2025. All rights reserved. Subject to Notice of rights (https://www.nice.org.uk/terms-and-conditions#notice-of-rights).

AI technology (manufacturer)	CE marking	Regions covered	Population	Other pathologies detected
RBfracture (Radiobotics)	Class IIa	Appendicular skeleton and ribs	2 years and over	Effusion of the knee and elbow, lipohaemarthrosis of the knee
TechCare Alert (Milvue)	Class IIa	Appendicular skeleton and ribs	No age limit	Dislocation, elbow joint effusion, pleural effusion, pulmonary opacity, pulmonary nodule, pneumothorax

Abbreviations: AI, artificial intelligence; CE, European Conformity.

The comparator

2.6 The comparator is standard care for fracture assessment in which the urgent care healthcare professional interprets the X-ray radiograph without AI assistance.

2.7 The reference standard is based on the consultant radiologist or reporting radiographer interpretation and report.

© NICE 2025. All rights reserved. Subject to Notice of rights (https://www.nice.org.uk/terms-and-conditions#notice-of-rights).

3 Committee discussion

The diagnostics advisory committee considered evidence on BoneView, qMSK, Rayvolve, RBfracture and TechCare Alert from several sources, including an external assessment report and an overview of that report. Full details are in the project documents for this guidance.

Patient and carer considerations

3.1 People may be anxious about the certainty of their diagnosis and the risk of being discharged with a missed fracture with or without the use of artificial intelligence (AI). In addition to the pain and potential clinical complications associated with a missed fracture, there are also practical stresses such as taking time off work or taking children out of school to reattend urgent care. Patient experts explained that if AI technologies could help improve diagnostic accuracy and reduce the risk of a misdiagnosis, then this would be a welcome benefit for patients.

3.2 Human interaction with a healthcare professional is an important factor for people to feel informed and reassured about their diagnosis. Patient experts explained that people may have different attitudes towards AI technologies and some people may distrust their use because they could be perceived as replacing human involvement. Clinical experts stated that, in practice, AI technologies would be used as a decision aid to assist healthcare professional fracture detection in urgent care (see section 2.5). They highlighted that the ionising radiation (medical exposure) regulations (IR[ME]R) state that clinical evaluation of X-rays requires a trained person. Therefore, AI technologies for fracture detection on X-rays cannot be used without human interpretation and so the level of human interaction would not change. The committee noted that people having X-rays for suspected fractures should be informed that AI software is being used. The role of healthcare professionals and AI software in interpreting the X-rays should also be explained. Patient and clinical experts also highlighted the importance of educating patients and healthcare professionals to understand the benefits and limitations of the software. The importance of shared decision making after AI-assisted diagnosis was also highlighted.

© NICE 2025. All rights reserved. Subject to Notice of rights (https://www.nice.org.uk/terms-and-conditions#notice-of-rights).

Clinical effectiveness

Evidence base

3.3 There were 16 studies that met the inclusion criteria for the clinical-effectiveness review. Most studies evaluated BoneView (8 studies) and RBfracture (5 studies). There was 1 study each on Rayvolve and TechCare Alert, and 1 study covering BoneView, Rayvolve and TechCare Alert together. No studies were identified for qMSK that compared interpretation of X-rays by healthcare professionals with or without use of the technology.

Diagnostic accuracy

3.4 Diagnostic accuracy studies typically found improved sensitivity of fracture detection, without reduced specificity, by healthcare professionals assisted by AI software compared with unassisted interpretation. For example, one of the key studies for BoneView (Duron et al. 2021), which reported estimates for emergency physicians interpreting mixed fracture types, indicated that sensitivity increased from 61% (unassisted) to 74% (assisted). Similar increases in sensitivity were seen for the other software when used by emergency care staff for mixed fractures. Bachmann et al. (2024) reported an increase in sensitivity from 74% unassisted to 83% when assisted by RBfracture, and Fu et al. (2024) reported an increase from 79% to 94% for Rayvolve. The Suite 2020 study reported a much smaller increase in sensitivity (92% to 95%) when using TechCare Alert, but the readers in this study were radiologists rather than emergency physicians. No key studies reported a decrease in specificity when using AI to assist fracture detection. The committee concluded that the available evidence suggested that AI technologies have the potential to improve the diagnostic accuracy of fracture detection by healthcare professionals. The committee noted that there was some uncertainty and wide variance in the estimates of sensitivity and specificity of the AI technologies in the studies because of variation in study designs.

3.5 Clinical experts explained that the diagnostic accuracy of unassisted fracture detection reported in the studies was lower than would be expected in clinical practice (see section 2.3). The committee noted that this could overestimate the

© NICE 2025. All rights reserved. Subject to Notice of rights (https://www.nice.org.uk/terms-and-conditions#notice-of-rights).

diagnostic accuracy of the AI technologies and therefore their clinical effectiveness. A clinical expert said that in most reader studies there is usually some heterogeneity in the diagnostic accuracy of unassisted healthcare professional review. So, it is unclear what should be considered a normal baseline estimate of unassisted diagnostic accuracy. The committee concluded that further evidence on the diagnostic accuracy of AI-assisted and unassisted fracture detection should be collected as part of a real-world evidence generation plan.

3.6 The committee concluded that the included studies were not entirely applicable to using AI technologies to help healthcare professionals detect fractures on X-rays in a UK urgent care setting. Most were retrospective, case-control studies. Clinical experts explained that retrospective studies may not represent the diagnostic accuracy of healthcare professional review in clinical practice. This is because in the studies the readers typically interpret X-rays in isolation rather than alongside the patient or patient history and case notes, as they would in clinical practice. The committee noted that none of the studies were done in a UK urgent care setting. The healthcare workers who interpreted the X-rays in the studies differed from those who would typically interpret X-rays in UK urgent care settings. For example, the studies examined the accuracy of radiologists with or without AI assistance, rather than emergency department healthcare professionals. So, it is uncertain how the technologies would perform in this setting. A clinical expert also highlighted that some of the studies included the AI software result as part of the reference standard.

Children and young people

3.7 The committee considered the limited evidence base for children and young people. It concluded that, similar to adults, AI technologies have the potential to improve the diagnostic accuracy of healthcare professionals' fracture detection in this subgroup. Only 2 of the key studies identified by the external assessment group (EAG) reported diagnostic accuracy data for children and young people. These studies indicated the potential of AI technologies to improve sensitivity without reducing specificity compared with unassisted fracture diagnosis. A study by Nguyen et al. (2022) evaluated BoneView and showed an increase in sensitivity from 73.2% (unassisted) to 82.7% (assisted) in a mixed reader group

© NICE 2025. All rights reserved. Subject to Notice of rights (https://www.nice.org.uk/terms-and-conditions#notice-of-rights).

(including radiologists) interpreting mixed fracture types. Bachmann et al. (2024) evaluated RBfracture and showed that sensitivity increased from 78% to 89%, also in a mixed reader group interpreting mixed fracture types. A clinical expert highlighted that in 1 study the unassisted diagnostic accuracy for children was higher than that reported for adults. They said that this was unusual and suggested that it may indicate a bias in the selection of cases or in the staff involved in interpreting the X-rays, leading to uncertainty in the results. A clinical expert explained that there are important differences in X-ray interpretation and fracture detection between children and young people and adults. There is wide variance in how children's bones can look on X-ray images and this can complicate fracture detection. They also highlighted that there is limited evidence in children younger than 2 years or when there is a suspicion that the injuries are a result of abuse. The clinical experts noted that cases of suspected physical abuse would be referred for further review by radiology and that the use of AI would not affect the escalation pathway.

System impact

3.8 The committee concluded that although more system-level impact data was needed, the risk of AI technologies negatively affecting the healthcare system is low. This is because the evidence suggests it is unlikely that AI use would lead to an increase in the rate of false referrals (see section 3.4). The only evidence on system-level impact was on X-ray reading times with and without AI assistance, which was available for 3 of the technologies (BoneView, Rayvolve and RBfracture). The committee noted that using the AI technologies resulted in reductions and increases of only a few seconds compared with unassisted readers. The clinical experts explained that reading-time estimates from the studies may have limited relevance to clinical practice. This is because in the studies, healthcare professionals interpreting the X-rays may only be looking at the X-ray in isolation (see section 3.6). In clinical practice they would take time to consider the patient history and may do a more detailed review of the suspected fracture site. The committee decided that other system-level effects, such as fracture clinic referral rates with and without AI assistance, would have more impact, and data on this could be collected as part of the evidence generation plan.

© NICE 2025. All rights reserved. Subject to Notice of rights (https://www.nice.org.uk/terms-and-conditions#notice-of-rights).

Cost effectiveness

Model structure

3.9 The EAG constructed an exploratory economic model to explore the potential cost effectiveness of AI-assisted fracture detection compared with unassisted diagnosis in an urgent care setting. The model consisted of 3 separate sub-models for the fracture sites that were considered to gain the greatest potential benefit from AI-assisted diagnosis, because the costs and clinical outcomes of these fractures differed substantially. These fracture sites were wrist and hand, ankle and foot, and hip. Each model comprised a decision tree incorporating the prevalence, sensitivity and specificity and cost per diagnosis for AI-assisted and unassisted fracture detection.

3.10 The committee concluded that the EAG's exploratory economic model structure and assumptions likely underestimated the impact of false-negative diagnoses. It noted that people with a false-negative diagnosis were assumed to reattend urgent care 2 to 4 weeks after their initial presentation, with no further disutilities assumed to occur in that time. The clinical experts explained that this was an oversimplification and did not reflect clinical practice. This is because a delay in treatment could result in changes to the injury, which may change further management. For example, a 2-week delay to treating a wrist fracture may result in callus formation which would then require a different kind of surgery, or a missed ankle fracture may require surgery in addition to a brace or cast. There is also a risk of further injury if people are discharged with an undiagnosed fracture, and they may re-present in other settings such as a GP surgery or physiotherapy. The committee noted that costs associated with further management because of delayed treatment were not captured in the economic model beyond the cost of an additional A&E appointment. These assumptions would therefore underestimate the benefit of improving fracture detection using AI technologies in the model results.

3.11 The committee concluded that the model overestimated the impact of false-positive diagnoses of hip fracture. The model assumed that false-positive diagnoses of hip fracture would result in unnecessary surgery. The clinical experts said that this was highly unlikely because further imaging such as CT or MRI would usually be requested if there was any uncertainty in the diagnosis. So,

© NICE 2025. All rights reserved. Subject to Notice of rights (https://www.nice.org.uk/terms-and-conditions#notice-of-rights).

the costs for this group are likely overestimated in the model.

Costs and clinical outcomes of fractures

3.12 The committee noted that because all the evidence used in the model was from retrospective studies, there was no data on the costs and clinical outcomes associated with misdiagnosed fractures. The EAG explained that because of this lack of evidence, the model assumed the only consequence of a missed fracture was pain. The committee concluded that the costs and clinical outcomes associated with missed fractures were uncertain but likely underestimated (see section 3.10). Further data on the costs and outcomes associated with fractures in urgent care could be collected as part of the evidence generation plan.

Diagnostic accuracy inputs

3.13 The baseline sensitivity and specificity estimates were taken either from Bousson et al. (2023) for BoneView, Rayvolve and TechCare Alert, or from Bachmann et al. (2024) for RBfracture and unassisted readers. The committee concluded that the model inputs for diagnostic accuracy were uncertain because of the study designs (see sections 3.5 and 3.6), which could have a large impact on the potential cost effectiveness of AI-assisted fracture detection. Bousson et al. was a retrospective study that only included radiologist readers and the reference standard included the AI results. The study by Bachmann et al. was also retrospective and used a case-control design. The committee noted that the accuracy of unassisted readers was lower than expected, so the difference in accuracy between AI-assisted and unassisted fracture detection may have been overestimated (see section 3.5). The committee stated that further evidence was needed on the diagnostic accuracy of AI-assisted and unassisted healthcare professional fracture detection in urgent care.

Cost inputs

3.14 The committee concluded that the true cost of implementing and using AI technologies for fracture detection was uncertain and further evidence was

© NICE 2025. All rights reserved. Subject to Notice of rights (https://www.nice.org.uk/terms-and-conditions#notice-of-rights).

needed on the cost of implementation in different urgent care centres. Some companies did not submit costs for the assessment, so the EAG used a notional cost of £1 per scan in the base case. A clinical expert stated that the economic model did not include set-up costs relating to NHS IT time and fees from the picture archiving and communications system (PACS) providers to ensure the new technology works correctly. These costs were variable depending on the centre but experts estimated they could be between £1,200 and £120,000. A clinical expert also explained that there are also ongoing cost and resource requirements associated with post-market surveillance. While this should be supported by companies, it still relies on NHS staff to collect this data. A clinical expert explained that from 2025 there will be additional financial support from the NHS, which may help relieve some of the cost impact of implementing AI technologies for fracture detection.

Plausibility of cost effectiveness

3.15 The committee decided it is plausible that the AI technologies could be cost effective if implemented in the NHS. This is because the available evidence suggests that they have the potential to improve sensitivity without reducing specificity compared with unassisted fracture diagnosis. In the base case, the committee noted that, overall, BoneView, RBfracture and TechCare Alert were associated with a positive incremental net health benefit compared with unassisted diagnosis at a threshold of £20,000 per quality-adjusted life year (QALY) gained. But in most cases, the 95% confidence intervals crossed zero, both for all separate fracture types and when considered together.

3.16 In the EAG's base case, Rayvolve had a negative incremental net health benefit. The committee noted that this was likely because it was modelled as having a lower specificity (67% to 75%) than unassisted fracture detection (87%), resulting in an increase in false-positive results and their associated costs. The diagnostic accuracy estimates used in the base case for Rayvolve were from the study by Bousson et al. (2023). The company (AZmed) stated that diagnostic accuracy estimates for Rayvolve from this study were unreliable because it used an outdated version of the algorithm. The committee considered the diagnostic accuracy estimates from the other key study that used Rayvolve (Fu et al. 2024). It noted that they showed improved sensitivity and little change in specificity with

© NICE 2025. All rights reserved. Subject to Notice of rights (https://www.nice.org.uk/terms-and-conditions#notice-of-rights).

the AI compared with unassisted readers. The committee concluded that because of the uncertainty in the diagnostic accuracy estimates, it was reasonable to assume that Rayvolve also had the potential to be cost effective (see section 3.13).

3.17 The committee recalled the uncertainty around the diagnostic accuracy estimates (see section 3.4). It noted that if the data was significantly overestimating the performance of the technologies, they would be less likely to be cost effective. In the scenario analyses, only the scenarios that changed the diagnostic accuracy significantly affected the model results.

3.18 The committee noted that for all fracture sites there was a minimal difference in QALYs between AI-assisted and unassisted diagnosis. The committee said that this is likely because the model underestimates the utility impact of a missed fracture (see section 3.10) and so may also underestimate the cost effectiveness of the AI technologies.

3.19 The committee also recalled the uncertainty in the costs because some companies did not provide a cost per scan, and the variability in estimates of set-up and implementation costs. However, in scenario analyses, the model results were not sensitive to small increases or decreases (less than £3) in the cost per scan. The EAG did a further scenario analysis that included additional installation and set-up costs. This applied a notional one-off set-up cost of £50,000 and assumed a 5-year lifespan of the software. The committee noted that the model results (see section 3.14) were not significantly affected by the £50,000 additional set-up cost over either a 5-year or 1-year period.

Risks

3.20 The committee concluded that although there were risks associated with the implementation of the AI technologies, they were relatively low or could be mitigated during the evidence generation period.

3.21 The committee decided that the clinical risk of implementing AI technologies to help detect fractures in urgent care is low. This is because they are used in addition to standard care, in which treatment decisions are made by healthcare

© NICE 2025. All rights reserved. Subject to Notice of rights (https://www.nice.org.uk/terms-and-conditions#notice-of-rights).

professionals. Also, the definitive X-ray reports are usually made by a radiologist or reporting radiographer, which AI would not replace. So, there are safety net systems in place to identify any potential fractures that may have been missed by the AI. The clinical experts explained that there would need to be clear local protocols in place when using AI technologies. This is to ensure that healthcare professionals are clear about what action to take when there is disagreement between the healthcare professional and AI.

3.22 The committee decided there was some risk associated with the cost of the AI technologies. This is because 2 companies did not provide pricing information, and there was uncertainty around the true cost of implementation and ongoing post-market surveillance. It noted that small changes to the cost per scan did not have a large effect on model results (see section 3.19). It said that when centres are implementing the technologies during the evidence generation period, they should consider the notional cost per scan used in the exploratory economic modelling.

3.23 Patient and clinical experts highlighted concerns that implementation of AI could lead to healthcare professionals becoming over-reliant on the technologies. They also highlighted that it may reduce the level of scrutiny for non-fracture-related conditions that can be detected on X-ray. The committee noted that this could potentially be mitigated if healthcare professionals interpret X-rays unassisted before viewing the AI results.

3.24 The committee considered the impact of AI on resource use. It noted there is a low risk that it may lead to an increase in fracture clinic referrals and requests for further imaging such as CT or MRI. This is because the evidence suggests it is unlikely that AI use would lead to an increase in the rate of false referrals.

Research considerations

3.25 The committee noted that, because the AI technologies are trained on different data sets and use different algorithms, it is likely that they all perform differently. Because there was very little evidence on how the AI technologies differed in terms of diagnostic accuracy (see section 3.4), it said that comparative, head-to-head studies of the software would be useful to help understand differences in

© NICE 2025. All rights reserved. Subject to Notice of rights (https://www.nice.org.uk/terms-and-conditions#notice-of-rights).

their diagnostic performance.

Equality considerations

3.26 There is limited evidence on using AI technologies to help detect fractures in children and young people, older people and people with conditions that affect bone health. The committee noted that the AI technologies should be used within their indications and clinicians should ensure that a technology is appropriate to use for the specific person they are assessing. Failure to do this could result in false reassurance and increase the risk of a fracture being missed.

3.27 Conditions that can affect bone health include:

- autoimmune and erosive arthropathies
- fibrous dysplasia
- myeloma
- osteoarthritis
- osteonecrosis
- osteoporosis
- osteogenesis imperfecta
- rickets or osteomalacia
- Paget's disease
- cancer with metastatic bone disease.

3.28 The clinical experts stated that the data sets used for training the AI technologies may not be representative of the local patient population. People from low socioeconomic status or minority groups may not be well represented in these sets. So, there is a risk that the diagnostic accuracy of the AI technologies may be reduced for these people. The committee noted that this was a potential limitation of the technologies and healthcare professionals should take this into

© NICE 2025. All rights reserved. Subject to Notice of rights (https://www.nice.org.uk/terms-and-conditions#notice-of-rights).

account when interpreting X-rays of people in these groups.

3.29 A patient expert highlighted the potential for indirect discrimination because of geographical availability and access. They raised concerns about whether the AI technologies would be deployed in smaller minor injuries units in rural areas as well as larger urgent treatment centres and emergency departments in urban areas. But the committee noted that AI software may help reduce variation in standard care by providing a consistent baseline for X-ray interpretation that is not affected by differences in staff experience or resources between centres.

Evidence gap review

3.30 Evidence gaps identified related to the intervention, the main outcomes including costs, and the population. The committee concluded that there was enough evidence on 4 of the AI technologies to demonstrate their potential benefit when used to help healthcare professionals detect fractures on X-rays in urgent care. It also concluded that the clinical risk of implementation is low (see sections 3.20 to 3.24). Important evidence gaps for all the AI technologies are:

- **Interventions**: the available evidence suggested that AI technologies have the potential to improve the diagnostic accuracy of healthcare professionals' fracture detection, but this was uncertain. Also, the accuracy of unassisted fracture detection reported in the studies was lower than would be expected in clinical practice. The committee concluded that further evidence on the diagnostic accuracy of AI-assisted and unassisted healthcare professional fracture detection in urgent care centres is needed. Further evidence is also needed on AI software failure rates and reasons for failure.
- **Outcomes:** there was no evidence on system-level outcomes. The committee noted that the outcome likely to have the largest system-level impact would be fracture clinic referral rates. It highlighted the need for further evidence on fracture clinic referrals with and without AI assistance. To better understand the clinical effectiveness of AI technologies for fracture detection, the clinical experts stated that further evidence was needed on clinically significant changes in treatment decisions for fractures detected using AI software. They also stated that evidence was needed on the detection or failure to

© NICE 2025. All rights reserved. Subject to Notice of rights (https://www.nice.org.uk/terms-and-conditions#notice-of-rights).

detect clinically significant non-fracture-related conditions by AI-assisted and unassisted healthcare professionals.

- **Costs:** because the evidence was from retrospective studies, there was no data on the costs and clinical outcomes associated with different fracture types and missed fractures. The true cost of implementing and using AI technologies for fracture detection is uncertain. These costs are important for understanding the financial investment that is needed and also the feasibility and sustainability of integrating AI technologies into routine healthcare. So, further evidence is needed on the cost of implementation and use of AI technologies in different urgent care centres.
- **Population:** the committee noted that there was limited evidence on using AI technologies to assist with fracture detection in the population subgroups identified in the scope. It highlighted the need for evidence generation on the diagnostic accuracy of AI-assisted healthcare professional fracture detection in different subgroups such as by age, sex, ethnicity, socioeconomic status, and conditions that affect bone health (see section 3.27).

Ongoing studies

3.31 The committee concluded that although there are several ongoing studies that may provide further evidence on the clinical effectiveness of AI technologies in fracture detection, they will not address all the evidence gaps identified (see section 3.30). The committee considered 2 ongoing studies evaluating BoneView. FRACT-AI (Clinicaltrials.gov, NCT06130397) is a retrospective multiple-reader, multiple-case study, due to complete in December 2024. A clinical expert explained that an advantage of FRACT-AI is that it will include a range of readers who are urgent care healthcare workers in a UK setting. Testing an artificial intelligence tool for childhood fracture detection on X-rays (ISRCTN12921105) is a retrospective, multicentre, multi-reader observational cohort study evaluating BoneView in paediatric fractures. Because both studies are retrospective, the committee stated that they will be unable to address evidence gaps relating to the post-diagnosis impact of AI-assisted fracture detection. The committee also noted that there were 5 NHS-based real-world data collection studies using RBfracture. Primary outcome measures that will be reported in these studies

© NICE 2025. All rights reserved. Subject to Notice of rights (https://www.nice.org.uk/terms-and-conditions#notice-of-rights).

include increases in productivity through time saving, rates of missed fractures, numbers of CT scans, inappropriate referrals to fracture clinics, and equivocal findings. These studies are due to complete between late 2024 and late 2025.

© NICE 2025. All rights reserved. Subject to Notice of rights (https://www.nice.org.uk/terms-and-conditions#notice-of-rights).

4 Committee members and NICE project team

Committee members

This topic was considered by the diagnostics advisory committee, which is a standing advisory committee of NICE.

Committee members are asked to declare any interests in the technologies to be evaluated. If it is considered there is a conflict of interest, the member is excluded from participating further in that evaluation.

The minutes of each committee meeting, which include the names of the members who attended and their declarations of interests, are posted on the NICE website.

Additional specialist committee members took part in the discussions for this topic:

Specialist committee members

Amanda Isaac
Consultant musculoskeletal radiologist and honorary associate professor, Guy's and St Thomas' NHS Foundation Trust and King's College London

Azizul Haque
Consultant trauma and orthopaedic surgeon, University Hospitals of Leicester NHS Trust

Claire Hart
Diagnostic radiographer, Swansea Bay University Health Board

David Lowe
Consultant in emergency medicine, Queen Elizabeth University Hospital, and honorary professor and clinical director of innovation, University of Glasgow

Garry Swann
Senior locum clinician, University Hospitals Birmingham NHS Foundation Trust

© NICE 2025. All rights reserved. Subject to Notice of rights (https://www.nice.org.uk/terms-and-conditions#notice-of-rights).

Kate Hawley
Lay specialist committee member

Nick Woznitza
Consultant radiographer, University College London Hospitals NHS Foundation Trust

Sarah Heaslip
Advanced physiotherapy practitioner, Salford Royal Foundation Trust

Sarah Markham
Lay specialist committee member

Susan Shelmerdine
Consultant paediatric radiologist, Great Ormond Street Hospital for Children NHS Foundation Trust

NICE project team

Each diagnostics evaluation is assigned to a team consisting of a technical analyst (who acts as the topic lead), a technical adviser and a project manager.

Simon Webster, Michael Kertanegara
Health technology assessment analysts

Jacob Grant
Health technology assessment adviser

Toni Gasse
Project manager

ISBN: 978-1-4731-6734-6

© NICE 2025. All rights reserved. Subject to Notice of rights (https://www.nice.org.uk/terms-and-conditions#notice-of-rights).
