# Supplementary material for: Advances in Artificial Intelligence for Wrist Joint Injury Diagnosis
Source: Int J Med Sci. 2026 Jul 1;23(8):2548–67. doi: 10.7150/ijms.134301 (PMC13411213; doi:10.7150/ijms.134301)
Supplement: Supplementary file 2 [file ijmsv23p2548s2.pdf]

Marketing Submission Recommendations for a Predetermined Change Control Plan for Artificial Intelligence-Enabled Device Software Functions

---

Guidance for Industry and Food and Drug Administration Staff

Document issued on August 18, 2025.

Document originally issued on December 4, 2024.

For questions about this document regarding CDRH-regulated devices, contact the Digital Health Center of Excellence by email at digitalhealth@fda.hhs.gov. For questions about this document regarding CBER-regulated devices, contact the Office of Communication, Outreach, and Development (OCOD) at 800-835-4709 or 240-402-8010, or by email at industry.biologics@fda.hhs.gov. For questions about this document regarding CDER-regulated products, contact the Center for Drug Evaluation and Research at 301-796-8936 or by email at druginfo@fda.hhs.gov. For questions about this document regarding combination products, contact the Office of Combination Products by email at combination@fda.gov.

FDA U.S. FOOD & DRUG ADMINISTRATION

U.S. Department of Health and Human Services
Food and Drug Administration
Center for Devices and Radiological Health
Center for Biologics Evaluation and Research
Center for Drug Evaluation and Research
Office of Combination Products in the Office of the Commissioner

Preface

Public Comment

You may submit electronic comments and suggestions at any time for Agency consideration to https://www.regulations.gov. Submit written comments to the Dockets Management Staff, Food and Drug Administration, 5630 Fishers Lane, Room 1061, (HFA-305), Rockville, MD 20852-1740. Identify all comments with the docket number FDA-2022-D-2628. Comments may not be acted upon by the Agency until the document is next revised or updated.

Additional Copies

CDRH

Additional copies are available from the Internet. You may also send an email request to CDRH-Guidance@fda.hhs.gov to receive a copy of the guidance. Please include the document number GUI00020049 and complete title of the guidance in the request.

CBER

Additional copies are available from the Office of Communication, Outreach, and Development (OCOD), Center for Biologics Evaluation and Research (CBER), by calling 800-835-4709 or 240-402-8010, by email, industry.biologics@fda.hhs.gov, or from the Internet at https://www.fda.gov/vaccines-blood-biologics/guidance-compliance-regulatory-information-biologics/biologics-guidances.

CDER

Additional copies are available from the Office of Communications, Division of Drug Information, Center for Drug Evaluation and Research (CDER), by calling 855-543-3784 or 301-796-3400, or by email, druginfo@fda.hhs.gov, or from the Internet at https://www.fda.gov/drugs/guidance-compliance-regulatory-information/guidances-drugs.

Table of Contents

I. Introduction ... 1
II. Background ... 2
III. Scope ... 5
IV. Definitions ... 8
A. Software Functions ... 8
B. Data Sets ... 8
C. Predetermined Change Control Plan ... 9
V. Policy for Predetermined Change Control Plans ... 10
A. Components of a PCCP ... 11
B. Establishing a PCCP ... 11
C. Identifying a PCCP in a Marketing Submission ... 13
D. Utilizing an Authorized PCCP to Implement Device Modifications ... 15
E. Modifying a Previously Authorized PCCP ... 18
F. Version Control and Maintenance of a PCCP for a Device ... 19
VI. Description of Modifications ... 20
A. Goals of the Description of Modifications Section ... 20
B. Content of the Description of Modifications Section ... 21
C. Types of Modifications ... 22
VII. Modification Protocol ... 23
A. Goals of the Modification Protocol Section ... 23
B. Content of the Modification Protocol Section ... 25
(1) Data management practices ... 25
(2) Re-training practices ... 26
(3) Performance evaluation ... 27
(4) Update procedures ... 28
C. Traceability Between the Description of Modifications Section and the Modification Protocol Section ... 29
VIII. Impact Assessment ... 30
Appendix A: Example Elements of Modification Protocol Components for AI-DSFs ... 31
(1) Data Management ... 31
(2) Re-Training ... 34
(3) Performance Evaluation ... 34

(4) Update Procedures 35
Appendix B: Example AI-DSF Scenarios Employing PCCPs 38
(1) Patient Monitoring Software 38
(2) Skin Lesion Software 40
(3) Ventilator Settings Software 41
(4) Image Acquisition Assistance Device 42
(5) Feeding Tube Placement Radiograph Analysis Software 43
(6) Optical Imaging System Co-packaged with Imaging Drug 44

Marketing Submission Recommendations for a Predetermined Change Control Plan for Artificial Intelligence-Enabled Device Software Functions

Guidance for Industry and Food and Drug Administration Staff

This guidance represents the current thinking of the Food and Drug Administration (FDA or Agency) on this topic. It does not establish any rights for any person and is not binding on FDA or the public. You can use an alternative approach if it satisfies the requirements of the applicable statutes and regulations. To discuss an alternative approach, contact the FDA staff or Office responsible for this guidance as listed on the title page.

I. Introduction

FDA has a longstanding commitment to develop and apply innovative approaches to the regulation of medical device software and other digital health technologies to ensure their safety and effectiveness.[1] As technology continues to advance all facets of healthcare, medical software incorporating artificial intelligence (AI), including the subset of AI known as machine learning (ML), has become an important part of many medical devices. This guidance is intended to provide a forward-thinking approach to promote the development of safe and effective AI-enabled devices.

[1] FDA regulates software that meets the definition of device, which is defined in section 201(h)(1) of the Federal Food, Drug, and Cosmetic Act (FD&C Act) as "an instrument, apparatus, implement, machine, contrivance, implant, in vitro reagent, or other similar or related article, including any component, part, or accessory, which is – recognized in the official National Formulary, or the United States Pharmacopoeia, or any supplement to them, intended for use in the diagnosis of disease or other conditions, or in the cure, mitigation, treatment, or prevention of disease, in man or other animals, or intended to affect the structure or any function of the body of man or other animals, and which does not achieve its primary intended purposes through chemical action within or on the body of man or other animals and which is not dependent upon being metabolized for the achievement of its primary intended purposes. The term 'device' does not include software functions excluded pursuant to section 520(o)" of the FD&C Act.

AI technologies have the potential to transform healthcare by deriving new and important insights from the vast amount of data generated during the delivery of healthcare every day. Medical device manufacturers[2] are using AI technologies to innovate their products to better assist healthcare providers and improve patient care. Examples of AI applications in medicine include earlier disease detection and diagnosis, development of personalized diagnostics and therapeutics, and development of assistive functions to improve the use of devices with the goal of improving user and patient experience.

FDA recognizes that the development of AI-enabled devices (also referred to as AI-enabled device software functions or AI-DSFs)[3] is an iterative process. This guidance describes an approach that would often be least burdensome[4] for manufacturers to support iterative improvement through modifications to an AI-DSF while continuing to provide a reasonable assurance of device safety and effectiveness. As such, this guidance demonstrates FDA's broader commitment to developing innovative approaches to the regulation of device software functions as a whole.

Specifically, this guidance provides recommendations on the information to include in a Predetermined Change Control Plan (PCCP) in a marketing submission for a device that includes one or more AI-DSFs. This guidance recommends that a PCCP describe the planned AI-DSF modifications, the associated methodology to develop, validate, and implement those modifications, and an assessment of the impact of those modifications. FDA reviews the PCCP as part of a marketing submission for a device to ensure the continued safety and effectiveness of the device without necessitating additional marketing submissions for implementing each modification described in the PCCP.

In general, FDA's guidance documents do not establish legally enforceable responsibilities. Instead, guidances describe the Agency's current thinking on a topic and should be viewed only as recommendations, unless specific regulatory or statutory requirements are cited. The use of the word *should* in Agency guidances means that something is suggested or recommended, but not required.

II. Background

In April 2019, FDA published the "Proposed Regulatory Framework for Modifications to Artificial Intelligence/Machine Learning (AI/ML)-Based Software as a Medical Device (SaMD) - Discussion Paper and Request for Feedback" ("2019 discussion paper").[5] The 2019 discussion paper describes FDA's foundation for a potential approach to premarket review for AI/ML-

---

[2] For purposes of this guidance, "manufacturer" is used in accordance with the definitions of manufacturer in 21 CFR Parts 803, 806, 807, and 820 and as described in FDA's guidance "Policy for Device Software Functions and Mobile Medical Applications."

[3] For purposes of this guidance, the terms "AI-enabled device" and "AI-DSF" are used interchangeably, and "device" refers to an AI-DSF unless otherwise stated. Additionally, reference to an "AI-DSF" is referring to a software function that meets the definition of device, as defined in section 201(h) of the FD&C Act. See Section IV. for details on definitions.

[4] See FDA's guidance "The Least Burdensome Provisions: Concept and Principles."

[5] Also available at FDA's website on "Artificial Intelligence and Machine Learning in Software as a Medical Device."

driven software modifications. The ideas delineated in the 2019 discussion paper and further described in this guidance leveraged practices from our premarket programs and relied on the International Medical Device Regulators Forum's risk categorization principles,[6] the FDA's benefit-risk framework,[7] risk management principles described in the Device Modifications guidances,[8] and a total product lifecycle approach.[9]

AI is a machine-based system that can, for a given set of human-defined objectives, make predictions, recommendations, or decisions influencing real or virtual environments. ML is an application of AI that is characterized by providing systems the ability to automatically learn and improve on the basis of data or experience, without being explicitly programmed. One of the greatest potential benefits of AI and ML resides in the ability to improve model performance through iterative modifications, including by learning from real-world data. To support the iterative development of AI-DSFs, and as part of the proposed framework presented in the 2019 discussion paper, FDA described a "Predetermined Change Control Plan" that could be included in a marketing submission for an AI/ML-based device. In this guidance, we provide recommendations on the marketing submission content for a PCCP for an AI-DSF, which generally includes: 1) a detailed description of the specific, planned device modifications, which is referred to as the "Description of Modifications"; 2) the associated methodology to develop, validate, and implement those modifications in a manner that ensures the continued safety and effectiveness of the device across the intended use populations, which is referred to as the "Modification Protocol"; and 3) the assessment of the benefits and risks of the planned modifications and risk mitigations, which is referred to as the "Impact Assessment."

The 2019 discussion paper received a substantial amount of feedback from a wide array of interested parties. General comments were received, as well as specific responses to 18 questions posed in the 2019 discussion paper.[10] Additionally, numerous articles in peer-reviewed journals discuss or reference the framework proposed in the 2019 discussion paper.[11]

FDA has also held a number of public meetings and workshops on AI/ML topics. On February 25-26, 2020, FDA held a Public Workshop on the "Evolving Role of Artificial Intelligence in Radiological Imaging" to discuss emerging applications of AI in radiological imaging, including

---

[6] See FDA's website on "Global Approach to Software as a Medical Device" and the International Medical Device Regulators Forum (IMDRF) document "Software as a Medical Device: Possible Framework for Risk Categorization and Corresponding Considerations."
[7] See FDA's guidance "Factors to Consider When Making Benefit-Risk Determinations in Medical Device Premarket Approval and De Novo Classifications" and FDA's guidance "Benefit-Risk Factors to Consider When Determining Substantial Equivalence in Premarket Notifications (510(k)) with Different Technological Characteristics."
[8] See FDA's guidances "Modifications to Devices Subject to Premarket Approval (PMA) - The PMA Supplement Decision-Making Process," "Deciding When to Submit a 510(k) for a Change to an Existing Device," or "Deciding When to Submit a 510(k) for a Software Change to an Existing Device," referred to as the "Device Modifications guidances" hereafter.
[9] See FDA's website on "Total Product Life Cycle for Medical Devices."
[10] For more information, see the 2019 discussion paper's public docket, FDA-2019-N-1185.
[11] For example, Gerke S et al., "The need for a system view to regulate artificial intelligence/machine learning-based software as medical device," NPJ Digital Medicine 3, 53 (2020); Harvey et al., "How the FDA Regulates AI," Academic Radiology 27, 58-61 (2020); and Subbaswamy et al., "From development to deployment: dataset shift, causality, and shift-stable models in health AI," Biostatistics 21, 345-352 (2020).

AI devices intended to automate the diagnostic radiology workflow as well as guide image acquisition. At this workshop, the Agency worked with interested parties, including patients, to identify both benefits and risks associated with the use of AI in radiological imaging, and discussed best practices for the validation of fully automated radiological imaging software and image acquisition devices.

On October 22, 2020, FDA held a Patient Engagement Advisory Committee meeting on "Artificial Intelligence and Machine Learning in Medical Devices" to further elicit input from a diverse group of patients. The Committee provided recommendations on AI/ML-enabled medical devices and how to foster patient trust in them, considering the diverse populations in which they are and will be used.

On October 14, 2021, FDA held a Public Workshop on "Transparency of Artificial Intelligence/Machine Learning-enabled Medical Devices" for patients, caregivers, and providers. The purpose of the workshop was to 1) identify unique considerations in achieving transparency for users of AI/ML-enabled medical devices and ways in which transparency might enhance the safety and effectiveness of these devices; and 2) gather input from various interested parties on the types of information that would be helpful for manufacturers to include in the labeling and public facing information of AI/ML-enabled medical devices, as well as other potential mechanisms for information sharing.

FDA continues to receive an increasing number of premarket submissions for devices leveraging AI and ML technologies, and the Agency expects this to increase over time. Moreover, since the 2019 discussion paper's publication, there has been strong interest in utilizing PCCPs for AI-enabled devices.

In light of the public health need to facilitate innovation for AI/ML-based devices while providing appropriate oversight for them, on January 12, 2021, the CDRH Digital Health Center of Excellence issued FDA's Artificial Intelligence/Machine Learning (AI/ML)-Based Software as a Medical Device Action Plan ("the Action Plan"). The Action Plan describes FDA's strategy for addressing AI/ML-based devices in a holistic, collaborative, and multidisciplinary manner. An important pillar of the Action Plan is the further advancement of the tailored regulatory framework for AI/ML-based devices that was proposed in the 2019 discussion paper.

Additionally, section 3308 of the Food and Drug Omnibus Reform Act of 2022, Title III of Division FF of the Consolidated Appropriations Act, 2023, Pub. L. No. 117-328 ("FDORA") enacted on December 29, 2022, added section 515C "Predetermined Change Control Plans for Devices" to the FD&C Act. Section 515C of the FD&C Act (21 U.S.C. 360e-4) has provisions regarding PCCPs for devices requiring premarket approval (PMA) or premarket notification (510(k)). For example, section 515C provides that a supplemental application (section 515C(a)) or new 510(k) (section 515C(b)) is not required for a change to a device that would otherwise require a PMA supplement or a new 510(k) if the change is consistent with a PCCP approved or cleared by FDA. Section 515C also provides that FDA may require that a PCCP include labeling for safe and effective use of a device as such device changes pursuant to such plan, notification

requirements if the device does not function as intended pursuant to such plan, and performance requirements for changes made under the plan.[12]

While under the FD&C Act FDA may approve or clear a PCCP for a variety of devices, this guidance provides recommendations specifically for PCCPs for AI-DSFs. These recommendations are based on the statutory authorities provided in the FD&C Act, including the provisions added by FDORA, as well as feedback obtained through our various interactions with interested parties.

III. Scope

This guidance is applicable to AI-DSFs that the manufacturer intends to modify over time. This includes AI-DSFs for which modifications to the AI model are implemented automatically (i.e., for which the modifications are implemented automatically by software, also known as "continuous learning"), AI-DSFs for which modifications to the AI model are implemented manually (i.e., involving steps that require human input, action, review, and/or decision-making, and therefore are not implemented automatically), or a combination of both.

While the recommendations and content in this guidance for PCCPs are intended to be broadly applicable to all AI-enabled devices, the majority of marketing submissions containing PCCPs that FDA has reviewed are submissions for devices that incorporate the subset of AI known as ML. As such, many of the recommendations in this guidance are tailored to devices that incorporate ML.

This guidance describes an approach that would often be least burdensome for manufacturers to support the ability to modify an AI-DSF while maintaining the safety and effectiveness of the device across the intended use populations. Specifically, this guidance includes recommendations on the information that should be included in the PCCP in a marketing submission[13] for an AI-DSF.

For purposes of this guidance, a PCCP includes those device modifications that generally would otherwise require a new marketing submission.[14,15] These modifications include those that could significantly affect,[16] or that otherwise affect,[17] the safety or effectiveness of the device,[18] unless

---

[12] Sections 515C(a)(3) and 515C(b)(3) of the FD&C Act.

[13] For purposes of this guidance, the term "marketing submission" includes a PMA application, 510(k) submission, or De Novo Classification request.

[14] For purposes of this guidance, unless otherwise stated, references to "device modifications" or "modifications" are those that generally would otherwise require a new marketing submission pursuant to 21 CFR 807.81(a)(3) and 21 CFR 814.39(a).

[15] For more information on whether a modification would require a new marketing submission, see the Device Modifications guidances.

[16] 21 CFR 807.81(a)(3).

[17] 21 CFR 814.39(a).

[18] In accordance with 21 CFR 807.81(a)(3), a 510(k) is required for significant changes or modifications to a device and include 1) those that "could significantly affect the safety or effectiveness of the device, e.g., a significant change or modification in design, material, chemical composition, energy source, or manufacturing process" or

those modifications are covered by an authorized PCCP. By including a PCCP in a marketing submission for an AI-DSF, manufacturers can prospectively specify and seek premarket authorization[19] for intended modifications to an AI-DSF without needing to submit additional marketing submissions or obtain further FDA authorization before implementing such modifications – provided the changes are implemented consistent with the PCCP that has been reviewed and established through a device marketing authorization (referred to hereafter as the "authorized PCCP").[20] In other words, obtaining FDA authorization of a PCCP as part of a marketing submission for an AI-DSF allows a manufacturer to modify its AI-DSF over time in accordance with the PCCP instead of obtaining separate FDA authorization for each significant change prior to each implementation.[21]

Because a PCCP is appropriate for device modifications that generally would otherwise require a new marketing submission,[22] this guidance does not address a plan that contains a proposal for modifications that would not require a new marketing submission. For such modifications, the Quality System regulation (QSR) (21 CFR Part 820)[23] requires manufacturers of finished medical devices to, among other things, document the change in the device master record.[24] For devices subject to PMA requirements, such modifications need to be reported to FDA in post-approval periodic reports required as a condition to approval of the device.[25]

Premarket authorization for an AI-DSF with a PCCP may be established through the PMA pathway (see section 515C(a) of the FD&C Act), the 510(k) pathway (see section 515C(b) of the FD&C Act), or the De Novo pathway (see section 513(f)(2) of the FD&C Act).[26] For devices

include 2) "a major change or modification in the intended use of the device." In accordance with 21 CFR 814.39(a), a PMA supplement is required for "change[s] affecting the safety or effectiveness of the device" unless an exception applies (see 21 CFR 814.39). For simplicity, in this guidance, the phrase "significant changes" or "significant modifications" refers to 21 CFR 807.81(a)(3). However, for devices subject to PMA requirements, the broader requirement pursuant to 21 CFR 814.39(a) of a "change affecting the safety or effectiveness" applies.

[19] For purposes of this guidance, the term "authorization" includes approval of a PMA application, clearance of a 510(k) submission, or grant of a De Novo Classification request.

[20] For purposes of this guidance, the term "authorized PCCP" refers to a PCCP that has been reviewed and established through a device marketing authorization. See Section IV. for more information on definitions.

[21] Sections 515C(a)(1) and 515C(b)(1) of the FD&C Act, 21 CFR 807.81(a)(3), and 21 CFR 814.39(a).

[22] 21 CFR 807.81(a)(3) and 21 CFR 814.39(a).

[23] On February 2, 2024, FDA issued a final rule amending the device QSR, 21 CFR Part 820, to align more closely with international consensus standards for devices (89 FR 7496). This final rule will take effect on February 2, 2026. Once in effect, this rule will withdraw the majority of the current requirements in Part 820 and instead incorporate by reference the 2016 edition of the International Organization for Standardization (ISO) 13485, Medical devices – Quality management systems – Requirements for regulatory purposes, in Part 820. As stated in the final rule, the requirements in ISO 13485 are, when taken in totality, substantially similar to the requirements of the current Part 820, providing a similar level of assurance in a firm's quality management system and ability to consistently manufacture devices that are safe and effective and otherwise in compliance with the FD&C Act. When the final rule takes effect, FDA will also update the references to provisions in 21 CFR Part 820 in this guidance to be consistent with that rule.

[24] See 21 CFR 820.181.

[25] See 21 CFR 814.39(b) and 814.82(a)(7) and FDA's guidance "Annual Reports for Approved Premarket Approval Applications (PMA)."

[26] The De Novo classification process allows FDA to classify a device into class I or II when general controls or general controls and special controls provide a reasonable assurance of safety and effectiveness, but for which there is no legally marketed predicate. The De Novo pathway, therefore, allows FDA to develop special controls that

subject to 510(k) requirements, in making a determination of substantial equivalence where the predicate device was authorized with a PCCP, the subject device must be compared to the version of the predicate device cleared or approved prior to changes made under the PCCP.[27]

Generally, the recommendations in this guidance apply to the device constituent[28] part of device-led[29] combination products,[30] when the device constituent part includes an AI-DSF. The recommendations in this guidance do not apply to the drug or biologic constituent part of device-led combination products. If a modification to the AI-DSF in a PCCP impacts the drug or biologic constituent part, we highly encourage early engagement with FDA. For such modifications and device-led combination products with a PCCP for an AI-DSF, the FDA review division will consult CBER or CDER, as appropriate.

FDA highly encourages early engagement regarding a proposed PCCP with the FDA review division that will review the AI-DSF; in particular, early engagement could be especially helpful for certain AI-DSFs, including combination products and high-risk, life-sustaining, life-supporting, or implantable devices. FDA encourages manufacturers to leverage the Q-Submission Program[31] for obtaining FDA feedback on a proposed PCCP for an AI-DSF prior to submitting a marketing submission. The FDA review division with purview over the AI-DSF will provide feedback on a proposed PCCP, including whether the scope of the modifications is appropriate for inclusion in a PCCP and, based on the statutory and regulatory requirements applicable to that AI-DSF, what evidence and information would ensure that the AI-DSF under that PCCP remains safe and effective.

This guidance is intended to provide recommendations on the information to include in a PCCP in a marketing submission for an AI-DSF. This guidance is not intended to provide a complete description of what may be necessary to include in a marketing submission for an AI-DSF.[32] The recommendations in this guidance do not change applicable statutory and regulatory standards, such as device clearance or approval standards, nor the applicable requirements, including marketing submission content requirements and the requirements for valid scientific evidence.[33] FDA recommends that you refer to other guidances, as applicable to a specific device, for recommendations on aspects of the submission beyond the PCCP.

This guidance is intended to provide recommendations on certain types of modifications that, at this time, FDA believes generally may be appropriate for inclusion in a PCCP for an AI-DSF. This guidance is not intended to delineate a comprehensive list of modifications FDA would consider appropriate for inclusion in a PCCP for an AI-DSF.

---

provide a reasonable assurance of the safety and effectiveness of the subject device. At this time, FDA expects that if it authorizes an AI-DSF with a PCCP via the De Novo pathway, the Agency would develop appropriate special controls, which may include specific requirements for a PCCP.

[27] See section 515C(c) of the FD&C Act.

[28] See 21 CFR 4.2.

[29] See 21 CFR 3.4 for information on lead center assignment.

[30] See 21 CFR 3.2(e).

[31] See FDA's guidance "Requests for Feedback and Meetings for Medical Device Submissions: The Q-Submission Program" hereafter referred to as the "Q-Submission Program."

[32] See, e.g., 21 CFR 807.87, 21 CFR 860.220, or 21 CFR 814.20.

[33] See, e.g., 21 CFR 807.87, 21 CFR 860.220, or 21 CFR 814.20, and 21 CFR 860.7(c).

Section IV. of this guidance defines certain terms as they are used for purposes of this guidance. Section V. of this guidance discusses the policy for PCCPs for AI-DSFs, including the components of a PCCP and where information about the PCCP should be included in the marketing submission for a device, and how to establish, implement, or modify a PCCP. Sections VI.-VIII. of this guidance provide an overview of the recommended content for each component of a PCCP for an AI-DSF. The Appendices provide key information to help manufacturers implement the recommendations in this guidance, including detailed questions and considerations for the recommended content of a Modification Protocol in a PCCP for an AI-DSF (Appendix A), as well as example scenarios for employing a PCCP for an AI-DSF (Appendix B).

IV. Definitions

This section defines certain terms as they are used for purposes of this guidance.

A. Software Functions

Artificial Intelligence (AI): A machine-based system that can, for a given set of human-defined objectives, make predictions, recommendations, or decisions influencing real or virtual environments. Artificial intelligence systems use machine- and human-based inputs to perceive real and virtual environments; abstract such perceptions into models through analysis in an automated manner; and use model inference to formulate options for information or action.[34]

Machine Learning (ML): An application of artificial intelligence that is characterized by providing systems the ability to automatically learn and improve on the basis of data or experience, without being explicitly programmed.[35]

Device Software Function (DSF): A software function that meets the device definition in section 201(h) of the FD&C Act.[36,37] As discussed in other FDA guidance, the term "function" is a distinct purpose of the product, which could be the intended use or a subset of the intended use of the product.[38]

Artificial Intelligence-Enabled Device Software Function (AI-DSF): A device software function (as defined above) that implements an AI model. AI-DSFs are the focus of this guidance.

B. Data Sets

Training Data: These data are used by the manufacturer of an AI-DSF in procedures and training algorithms to build an AI model, including to define model weights, connections, and

---

[34] 15 U.S.C. 9401(3).
[35] 15 U.S.C. 9401(11).
[36] See footnote 1.
[37] Device software functions may include Software as a Medical Device (SaMD) and Software in a Medical Device (SiMD). See FDA's website on "Software as a Medical Device (SaMD)."
[38] See FDA's guidance "Multiple Function Device Products: Policy and Considerations."

components.[39] These data typically should be representative of the proposed intended use populations (e.g., with respect to race, ethnicity, disease severity, sex, age, or others, as appropriate) and intended environments.

Tuning Data: These data are typically used by the manufacturer of an AI-DSF to evaluate a small number of trained AI models. This process involves exploring various aspects, including different architectures or hyperparameters.[40] The tuning phase happens before the testing phase of the AI-DSF and is part of the training process. While the AI and ML communities sometimes use the term "validation" to refer to the tuning data and phase, FDA does not use the word "validation" in this context.

Test Data: These data are used to characterize the performance of an AI-DSF. These data are never shown to the algorithm during training and are used to estimate the AI model's performance after training.[41] Testing is conducted to generate evidence to establish the performance of an AI-DSF before it is deployed or marketed. The testing phase is also expected to provide evidence to demonstrate a reasonable assurance of safety and effectiveness of an AI-DSF before it is deployed or marketed. These data typically should be representative of the proposed intended use populations (e.g., with respect to race, ethnicity, disease severity, sex, age, or others, as appropriate) and intended environments. Test data should be independent of data used for training and tuning and should generally be from multiple sites different from those that were used to generate training and tuning data.

C. Predetermined Change Control Plan

Predetermined Change Control Plan (PCCP): The documentation describing what modifications will be made to a device and how the modifications will be assessed. The modifications described in the PCCP include device modifications that would otherwise require a PMA supplement or new premarket notification.[42] The PCCP includes a Description of Modifications, Modification Protocol, and Impact Assessment. PCCPs for AI-DSFs are the focus of this guidance.

Authorized Predetermined Change Control Plan (Authorized PCCP): A PCCP that has been reviewed and established through a device marketing authorization. An authorized PCCP is a technological characteristic of the authorized device with which it was established.

Modification Protocol: The documentation describing the methods that will be followed when developing, validating, and implementing modifications delineated in the Description of Modifications section of the PCCP. The Modification Protocol includes the verification and validation activities (including pre-defined acceptance criteria) for those modifications and is intended to provide a step-by-step delineation of how the modifications included in the PCCP will be implemented while ensuring the device remains safe and effective.

---

[39] Adapted from the IMDRF document "Machine Learning-enabled Medical Devices: Key Terms and Definitions."
[40] Adapted from IEEE 2802 *Standard for Performance and Safety Evaluation of Artificial Intelligence Based Medical Devices: Terminology*.
[41] Adapted from the IMDRF document "Machine Learning-enabled Medical Devices: Key Terms and Definitions."
[42] Section 515C(a)(2) and 515C(b)(2) of the FD&C Act.

Impact Assessment: The documentation of the assessment of the benefits and risks of implementing a PCCP, as well as the plan for risk mitigation.

V. Policy for Predetermined Change Control Plans

Software development is an iterative process, and FDA appreciates that manufacturers of device software functions strive to continually improve and update their devices. Manufacturers should evaluate the impact of modifications to their devices and must generally submit a marketing submission when device modifications affect the intended use of the device or could significantly affect the safety or effectiveness of the device.[43]

An authorized PCCP specifies planned modifications that, if not included in a PCCP, could otherwise require a new marketing submission pursuant to 21 CFR 807.81(a)(3) and 21 CFR 814.39(a), and consistent with the Device Modifications guidances. An authorized PCCP should include the following sections, which will be further described in Sections VI. through VIII. of this guidance:

- Description of Modifications (Section VI.) – The specifications for the characteristics and performance of the planned modifications to the device;
- Modification Protocol (Section VII.) – The associated verification and validation testing activities that will support the planned modifications and acceptance criteria to assure the device remains safe and effective across the intended use populations; and
- Impact Assessment (Section VIII.) – The assessment of the benefits and risks of implementing a PCCP, as well as the plan for risk mitigation.

Because modifications that are specified and implemented in accordance with an authorized PCCP were reviewed and authorized through the marketing submission containing the PCCP, the modifications can be implemented to the AI-DSF without triggering the need for a new marketing submission.[44]

FDA would consider it to be a deviation from the authorized PCCP in circumstances where the PCCP is not followed or cannot be followed.[45] Deviations from the authorized PCCP could significantly affect the safety or effectiveness of the device. This could include, for example, issues related to the Modification Protocol, such as data management or re-training failure, or failure to meet pre-specified performance criteria. Device modifications that would not require a marketing submission would not be considered a deviation from an authorized PCCP.[46] However, significant modifications made to an AI-DSF that are not specified in, or implemented in accordance with, the authorized PCCP likely require a new marketing submission prior to

[43] 21 CFR 807.81(a)(3) and 21 CFR 814.39(a).

[44] Sections 515C(a)(1) and 515C(b)(1) of the FD&C Act, 21 CFR 807.81(a)(3), and 21 CFR 814.39(a).

[45] FDA would not consider it to be a deviation from the authorized PCCP in situations where a manufacturer chooses not to implement modifications in their authorized PCCP or where a manufacturer chooses to submit a new marketing submission for a device modification in lieu of using their authorized PCCP.

[46] See Section V.D. below for further details on implementing device modifications that may or may not require a new marketing submission in accordance with 21 CFR 807.81(a)(3) and 21 CFR 814.39(a).

implementation of the modification.[47] Deviations from the authorized PCCP reviewed in the marketing submission would generally cause the device to be adulterated and misbranded under sections 501(f)(1) and 502(o) of the FD&C Act, respectively. The introduction or delivery for introduction into interstate commerce of any food, drug, device, tobacco product, or cosmetic that is adulterated or misbranded is prohibited under section 301(a) of the FD&C Act, and where appropriate, FDA may take legal or regulatory action against violations of prohibited acts, including, without limitation, seizure or injunction.

A. Components of a PCCP

A PCCP should consist of a detailed Description of Modifications, a Modification Protocol, and an Impact Assessment (see Sections VI. – VIII. of this guidance) because these components are intended to provide FDA with information that will enable our review of the proposed modifications. When developing a PCCP, manufacturers should consider the intended use populations (with respect to race, ethnicity, disease severity, sex, age, or others, as appropriate) and intended environments of use, so that the device continues to reflect these populations and environments as the device is modified. The detailed Description of Modifications should outline the specific, planned modifications that may be made to the AI-DSF. This includes defining the specifications for the characteristics and performance of the planned modifications to the device. The Modification Protocol should describe the verification and validation activities, including pre-defined acceptance criteria, that will support each modification to assure the device remains safe and effective across the intended use populations. The Impact Assessment helps to tie the Description of Modifications to the Modification Protocol in that the Impact Assessment identifies the benefits and risks introduced by the specified, planned modifications and addresses how the verification and validation activities of the Modification Protocol will continue to assure the safety and effectiveness of the device. The detailed Description of Modifications, Modification Protocol, and Impact Assessment are all interrelated components of a PCCP. The Appendices provide key information to help manufacturers implement the recommendations in this guidance, including detailed questions and considerations for the recommended content of a Modification Protocol in a PCCP (Appendix A), as well as example scenarios for employing a PCCP for an AI-DSF (Appendix B).

B. Establishing a PCCP

Premarket authorization for an AI-DSF with a PCCP must be established through the PMA pathway, 510(k) pathway, or De Novo pathway, as appropriate, as a PCCP must be reviewed and established as part of a marketing authorization[48,49] for a device prior to a manufacturer implementing any modifications under that PCCP. Correspondingly, submission types for which FDA does not make an affirmative decision (i.e., authorization) would not be appropriate to establish a PCCP. In general, FDA considers the following submission types to be appropriate to establish a PCCP:

---

[47] 21 CFR 807.81(a)(3) and 21 CFR 814.39(a).
[48] See sections 513(f)(2) and 515C of the FD&C Act.
[49] This includes marketing authorization for a device and PCCP where the device or a derivative thereof has yet to be introduced into interstate commerce, or marketing authorization for a device or a derivative thereof has been introduced into interstate commerce, and for which is being modified to add a PCCP.

- For AI-DSFs subject to PMA requirements:
  - Original PMA application
  - Modular PMA application, where a PCCP comprises a module of review
  - 180-Day PMA supplement
  - Panel Track PMA supplement
  - Real-Time PMA supplement, where a PCCP comprises minor changes and the manufacturer and FDA agree that the review can be achieved in a real-time setting[50,51]
- For AI-DSFs subject to 510(k) requirements:
  - Traditional 510(k) submission
  - Abbreviated 510(k) submission[52]
- For AI-DSFs subject to De Novo requirements:
  - Original De Novo request

A PCCP is authorized as part of the device marketing authorization. FDA must reach a determination[53] of reasonable assurance of safety and effectiveness or substantial equivalence in review of the device, including each modification specified in the PCCP, for the PCCP to be authorized with the device.

As manufacturers implement modifications included in an authorized PCCP, FDA expects manufacturers to implement the modifications consistent with their authorized PCCP, i.e., in accordance with their Modification Protocol. A manufacturer must implement any changes in accordance with their quality system.[54] A manufacturer's quality system is critical for change management processes for a device, especially for devices that include a PCCP, because a PCCP includes modifications that generally would otherwise require a new marketing submission.[55] Compliance with the QSR is essential in order for a manufacturer to implement modifications consistent with their authorized PCCP and failure to do so could potentially present a serious risk to human health.

Under section 515C(a)(2) of the FD&C Act, FDA may approve a PCCP submitted in a PMA. Under section 515(d)(2)(C) of the FD&C Act, FDA must deny approval of a PMA if FDA finds that the methods used in, or the facilities or controls used for, the manufacture, processing, packing, or installation of such device do not conform to the QSR requirements. Thus, consistent with sections 515C(a)(2) and 515(d)(2)(C) of the FD&C Act, FDA must deny approval of a PCCP submitted in a PMA if FDA finds that the methods used in, or the facilities or controls

---

[50] Section 737(4)(D) of the FD&C Act defines a real-time supplement as "a supplement to an approved premarket application or premarket report under section 515 that requests a minor change to the device, such as a minor change to the design of the device, software, sterilization, or labeling, and for which the applicant has requested and the agency has granted a meeting or similar forum to jointly review and determine the status of the supplement."

[51] See FDA's guidance "Real-Time Premarket Approval Application (PMA) Supplements."

[52] See FDA's guidance "The Abbreviated 510(k) Program."

[53] See, e.g., sections 513(i)(1) and 515C of the FD&C Act and 21 CFR 860.7.

[54] 21 CFR Part 820.

[55] Sections 515C(a)(2) and 515C(b)(2) of the FD&C Act, 21 CFR 807.81(a)(3), and 21 CFR 814.39(a).

used for, the manufacture, processing, packing, or installation of the subject device do not conform to the QSR requirements.

Under section 515C(b)(2) of the FD&C Act, FDA may clear a PCCP submitted in a 510(k). Generally, under section 513(f)(5) of the FD&C Act, FDA may not withhold a determination of the initial classification of a device under section 513(f)(1) of the FD&C Act because of, among other things, a finding that the facility in which the device is manufactured is not in compliance with the QSR. However, also under section 513(f)(5), for devices subject to 510(k), FDA may withhold a substantial equivalence determination if FDA finds that there is a substantial likelihood that the failure to comply with QSR will potentially present a serious risk to human health. Thus, consistent with sections 515C(b)(2) and 513(f)(5) of the FD&C Act, FDA may under certain case-by-case circumstances withhold clearance of a PCCP submitted in a 510(k) based on findings in the regulatory history of the manufacturer that demonstrate failure to comply with QSR.

For devices subject to 510(k) requirements, the determination of substantial equivalence includes, among other requirements, a comparison between the technological characteristics of the predicate device and the subject device.[56] In FDA's determination of substantial equivalence, FDA considers the PCCP to be part of the technological characteristics of the device. For 510(k) submissions, in making a determination of substantial equivalence where the predicate device was authorized with a PCCP, the subject device must be compared to the version of the predicate device cleared or approved prior to changes made under the PCCP.[57] Once a 510(k) for a device that includes modifications that have been implemented consistent with the authorized PCCP has been cleared in a subsequent marketing submission, such device can now serve as an eligible predicate device. The PCCP can be considered during the 510(k) review process at multiple points in the decision tree to address the critical questions in the 510(k) Decision-Making Flowchart.[58] In general, FDA anticipates that the PCCP will primarily be reviewed after FDA finds that the intended use of the subject device and the predicate device are the same, to help determine whether the devices have different technological characteristics that do not raise different questions of safety and effectiveness.[59]

FDA encourages manufacturers to leverage the Q-Submission Program to obtain FDA feedback on a proposed PCCP for a device and submission type prior to submitting a marketing submission. While manufacturers are encouraged to discuss their plans through a Pre-Submission, FDA does not authorize a PCCP in a Pre-Submission.

C. Identifying a PCCP in a Marketing Submission

The PCCP should be included as a standalone section within the marketing submission, with a title and version number. Additionally, it should be prominently included and discussed in the cover letter and included in the marketing submission's table of contents as "Predetermined

---

[56] See section 513(i) of the FD&C Act.
[57] See section 515C(c) of the FD&C Act.
[58] See FDA's guidance "The 510(k) Program: Evaluating Substantial Equivalence in Premarket Notifications [510(k)]" Appendix A, Decision Points 1 through 4.
[59] See id. at Decision Points 5a and 5b.

Change Control Plan." The PCCP should be discussed in the marketing submission as part of the device description, labeling, and relevant sections used for the assessment of reasonable assurance of safety and effectiveness or the substantial equivalence comparison. Any information pertaining to the PCCP content included outside of the PCCP section should include appropriate references to the PCCP section.

Device labeling must comply with applicable statutes and regulations.[60] FDA may require that a device with an authorized PCCP include labeling required for safe and effective use of the device as such device changes pursuant to such plan,[61] excluding, as appropriate, trade secret and confidential commercial information. For AI-DSFs with an authorized PCCP, the labeling should explain that the device incorporates machine learning and has an authorized PCCP so that users are aware that the device may require the user to perform software updates, and that such software updates may modify the device's performance, inputs, or use. Information on the AI-DSF and its authorized PCCP in the labeling is important in order for a user to use the device safely and effectively for the purposes for which it is intended. In particular, information on the device's authorized PCCP may be necessary for a user to understand changes in the device and to continue to use the device safely and effectively across the intended use populations and intended environments as the device changes pursuant to the authorized PCCP.

FDA recommends that the labeling include a statement that the device has an authorized PCCP. When appropriate, including as modifications are implemented consistent with an authorized PCCP, FDA recommends that the labeling related to the PCCP be updated to include the relevant information listed below for the device and the PCCP so that users may be aware of modifications that have been implemented that impact use of the device:

- A description of the implemented modifications, including a summary of current device performance, a description of the relevant data (training, tuning, and test data) as applicable used to implement a modification, associated inputs/outputs, validation requirements, and related evidence;
- A description of how the modifications were implemented; and
- A description of how users will be informed of implemented modifications, including, for example, updated instructions for use or a version history.

When utilizing an authorized PCCP to implement device modifications, the manufacturer should update the labeling for the device as specified in the authorized PCCP.[62]

---

[60] 21 CFR Part 801 (Labeling) and 21 CFR Part 809 (In Vitro Diagnostic Products for Human Use). See, e.g., 21 CFR 801.5 (requiring that labeling include adequate directions for use); 21 CFR 801.109(c) (for prescription devices, requiring that labeling include any relevant hazards, contraindications, side effects, and precautions under which practitioners licensed by law to administer the device can use the device safely and for the purpose for which it is intended); and 21 CFR 809.10(b)(6) (for in vitro diagnostic products, requiring labeling accompanying any instruments use or function, installation procedures, performance characteristics and specifications, service and maintenance information, etc.).

[61] See sections 515C(a)(3), 515C(b)(3), and 513(f)(2) of the FD&C Act.

[62] See Section VII.B.(4) for recommendations on update procedures in a Modification Protocol, which should address how labeling will be updated when modifications are implemented, as appropriate.

The PCCP should be described in publicly available device summaries including, for example, the PMA summary of safety and effectiveness document (SSED) and approval order,[63] 510(k) summary,[64,65] or De Novo decision summary.[66] Details of the PCCP should be included in sufficient detail in the public-facing documents to support transparency to users of the assessment of reasonable assurance of safety and effectiveness or the substantial equivalence comparison for the device and the device's specifications, excluding, as appropriate, trade secret and confidential commercial information. In addition, FDA recommends public-facing documents include a summary of the following information regarding the PCCP, as appropriate:

- Planned modifications;
- Testing methods;
- Validation activities and performance requirements to be met in order for modifications to be implemented; and
- Means by which users will be informed of device modifications implemented in accordance with the authorized PCCP.

D. Utilizing an Authorized PCCP to Implement Device Modifications

FDA expects manufacturers to follow their authorized PCCP, and manufacturers are required to follow applicable legal requirements regarding the device and its authorized PCCP. In general, a PCCP should be evaluated within the existing risk management framework of the device and must be implemented in accordance with the manufacturer's quality system.[67]

Figure 1 depicts the process for implementing a modification to a device with an authorized PCCP. Manufacturers should first consider whether the particular modification is or is not consistent with the authorized PCCP; FDA considers a modification to be consistent with the authorized PCCP when the modification has been specified in the Description of Modifications included in the PCCP *and* has been implemented in accordance with the Modification Protocol.

---

[63] In accordance with 21 CFR 814.9(e), "FDA will make available to the public … a detailed summary of information submitted to FDA respecting the safety and effectiveness of the device that is the subject of the PMA and that is the basis for the order."

[64] In accordance with 21 CFR 807.92, "a 510(k) summary shall be in sufficient detail to provide an understanding of the basis for a determination of substantial equivalence." This includes, but is not limited to, a description of the device, and for those 510(k) submissions in which a determination of substantial equivalence is also based on an assessment of performance data, non-clinical tests, and clinical tests.

[65] If a sponsor chooses to submit a 510(k) Statement rather than a 510(k) Summary, the sponsor should provide to requestors all PCCP information in the 510(k) that supports transparency to users of FDA's determination of substantial equivalence for the device and its specifications, as such information constitutes safety and effectiveness information. See 21 CFR 807.93 for requirements on the content and format of a 510(k) Statement.

[66] The De Novo decision summary is intended to present an objective and balanced summary of the scientific evidence that served as the basis for the FDA's decision to grant a De Novo request; see FDA's website on De Novo Classification Request.

[67] Manufacturers are required to comply with the QSR (21 CFR Part 820). The device and PCCP must be implemented consistent with 21 CFR Part 820, including, but not limited to design controls (21 CFR 820.30), nonconforming products (21 CFR 820.90), and corrective and preventative action (21 CFR 820.100). The QSR also includes requirements to review and approve modifications to device design and production (21 CFR 820.30 and 820.70), and requirements to document changes and approvals in the device master record (21 CFR 820.181).

If the particular modification is consistent with the authorized PCCP, a new marketing submission is not necessary; the modification can be implemented in accordance with the Modification Protocol, and the manufacturer should document that modification and the analysis in accordance with the manufacturer's quality system.

If the particular modification is not consistent with the authorized PCCP – including if the modification is not included in the authorized PCCP or if the modification is included in the authorized PCCP but is not implemented in accordance with the methods and specifications described in the Modification Protocol – the manufacturer should then proceed to evaluate the particular modification as described below. As part of the review of the particular modification, manufacturers should consider how implementation of the particular modification may affect the PCCP for the device by reviewing the Impact Assessment of the PCCP to determine if the particular modification introduces or significantly modifies risk mitigations for the PCCP.

- If the modification is not included in the authorized PCCP, the manufacturer should proceed based on their evaluation of the particular modification in accordance with applicable FDA statutory and regulatory requirements and after consulting the Device Modifications guidances.
- If the modification is included in the authorized PCCP but is not implemented in accordance with the methods and specifications described in the Modification Protocol, in most cases, because modifications included in a PCCP are those that would generally otherwise require a new marketing submission, it is likely that a new marketing submission will be required before the manufacturer can implement the change.[68]

As described in the introduction of Section V. of this guidance, FDA would consider it to be a deviation from the authorized PCCP in circumstances where the PCCP is not followed or cannot be followed.[69] Deviations from the authorized PCCP could significantly affect the safety or effectiveness of the device. Significant modifications made to a device that are not specified in, or implemented in accordance with, the authorized PCCP likely require a new marketing submission.[70] If a manufacturer believes that the deviation from their authorized PCCP is not significant, we strongly encourage discussion with the appropriate FDA review division. In general, manufacturers may contact the appropriate FDA review division for a discussion about a proposed modification and whether it may be considered consistent with the authorized PCCP.

If, after review of applicable FDA statutory and regulatory requirements a new marketing submission is required,[71] the appropriate marketing submission could request authorization for:

- A modification to the authorized PCCP[72] (see Section V.E. of this guidance); and/or

---

[68] 21 CFR 807.81(a)(3) and 21 CFR 814.39(a).
[69] FDA would not consider it to be a deviation from the authorized PCCP in situations where a manufacturer chooses not to implement modifications in their authorized PCCP or where a manufacturer chooses to submit a new marketing submission for a device modification in lieu of using their authorized PCCP.
[70] 21 CFR 807.81(a)(3) and 21 CFR 814.39(a).
[71] 21 CFR 807.81(a)(3) and 21 CFR 814.39(a)
[72] A change to the authorized PCCP could include a change in the Description of Modifications, the Modification Protocol, and/or the Impact Assessment, as appropriate.

- The modified device (i.e., a device modification not implemented through a PCCP).

In each of these cases, the marketing submission for the modification must include the appropriate marketing submission requirements[73] for the device. If the manufacturer requests authorization for a modification to the authorized PCCP, the manufacturer must also submit the proposed, modified PCCP for the device.[74] If the manufacturer requests authorization for the modified device, the manufacturer must also submit the proposed PCCP for the modified device.[75] In both scenarios, the manufacturer must obtain FDA authorization for the device and proposed PCCP before implementing the PCCP.[76]

See Appendix B for example scenarios for implementing modifications to an AI-DSF with an authorized PCCP.

[73] See, e.g., 21 CFR 807.87, 21 CFR 860.220, or 21 CFR 814.20. In general, manufacturers may provide references in the marketing submission to prior marketing submissions for content that remains unchanged, as appropriate.
[74] Sections 515C(a)(2) and 515C(b)(2) of the FD&C Act.
[75] Sections 515C(a)(2) and 515C(b)(2) of the FD&C Act.
[76] Section 515C of the FD&C Act, 21 CFR 807.81(a)(3), and 21 CFR 814.39(a).

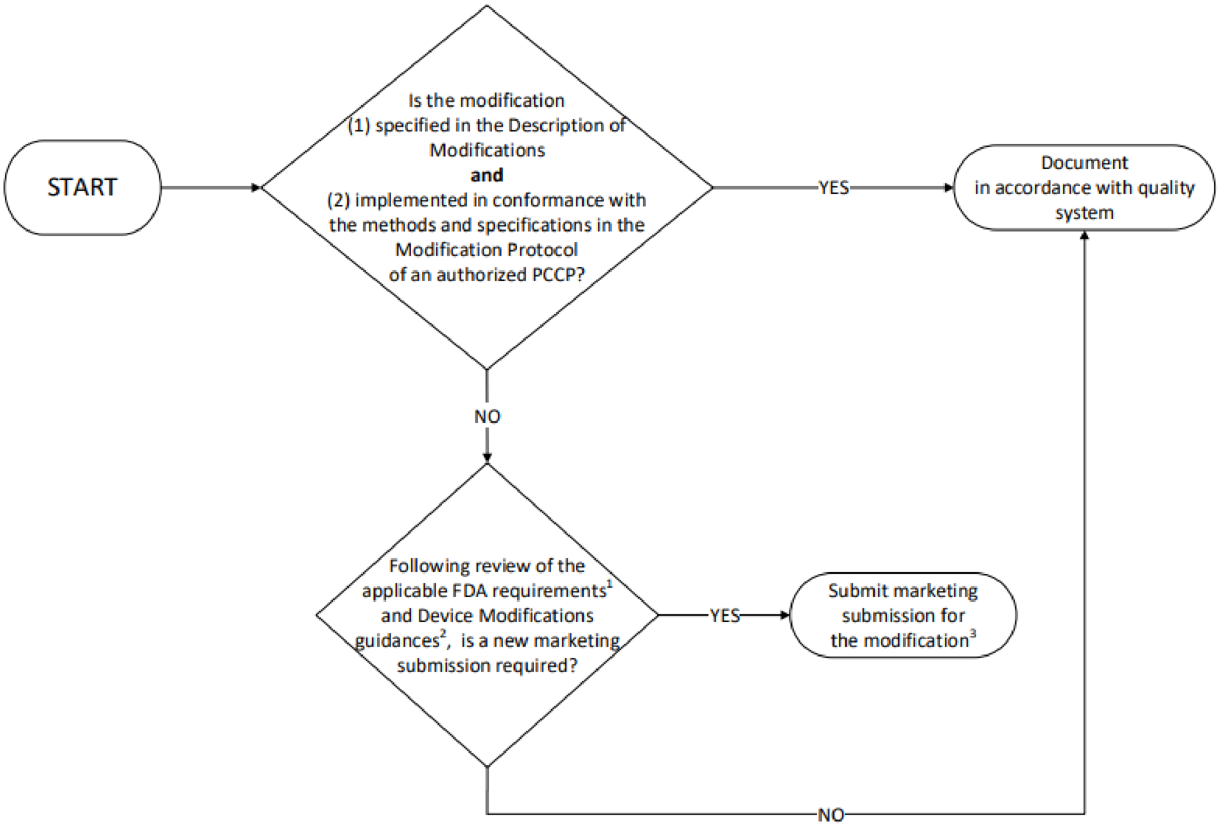

[1] 21 CFR 807.81(a)(3) and 21 CFR 814.39(a).

[2] See the FDA guidances "Modifications to Devices Subject to Premarket Approval (PMA) – The PMA Supplement Decision-Making Process," "Deciding When to Submit a 510(k) for a Change to an Existing Device," or "Deciding When to Submit a 510(k) for a Software Change to an Existing Device."

[3] For the modified device to have a PCCP, a PCCP should be submitted with the marketing submission so that the device and PCCP can be authorized together.

This flowchart is not intended to be used as a 'stand-alone' document and should only be considered in conjunction with the accompanying text in this guidance.

Figure 1: Implementing a Modification to a Device with an Authorized PCCP

E. Modifying a Previously Authorized PCCP

FDA believes that modifications to an authorized PCCP will generally constitute changes to the AI-DSF that would otherwise require a new marketing submission.[77] In other words, FDA anticipates that modifications to a PCCP will need to be reviewed and established as part of the marketing submission for the modified device because a modification to the PCCP will generally significantly affect the safety or effectiveness of the device.[78]

[77] Section 515C(a)(2) and 515C(b)(2) of the FD&C Act, 21 CFR 807.81(a)(3), and 21 CFR 814.39(a).
[78] Section 515C(a)(2) and 515C(b)(2) of the FD&C Act, 21 CFR 807.81(a)(3), and 21 CFR 814.39(a).

For a manufacturer who would like to modify their PCCP for a previously authorized device with a PCCP,[79] the marketing submission must include the appropriate marketing submission requirements[80] and the proposed, modified PCCP for the device.[81] We recommend that manufacturers provide a summary of the changes to the authorized PCCP, and where practicable, a tracked changes version compared to the authorized PCCP. In general, FDA considers the PMA supplement and 510(k) submission types included in Section V.B. of this guidance to be appropriate to modify a PCCP. In addition to those submission types, for devices subject to 510(k) requirements, a special 510(k) submission may be appropriate to modify a PCCP where the modifications to a PCCP comprise changes to the manufacturer's own device and PCCP and where well-established methods are available to evaluate the change to the PCCP.[82]

FDA intends to focus its review on the aspects of the device that are most significantly modified.[83] For example, if the device has been relatively unchanged since FDA's prior authorization and a modified PCCP is proposed, FDA would focus its review on the modified PCCP. Manufacturers may discuss proposed changes to the PCCP with the appropriate FDA review division through the Q-Submission Program.

F. Version Control and Maintenance of a PCCP for a Device

At this time, as manufacturers gain experience developing and implementing PCCPs, FDA anticipates that review of a marketing submission that includes a PCCP will be interactive. It is possible that a PCCP submitted as part of a marketing submission may need revisions before FDA can make a determination[84] of reasonable assurance of safety and effectiveness or substantial equivalence in review of the device, including each modification in the PCCP, for the PCCP to be authorized with the device. FDA should work with the manufacturer to revise the PCCP, and will do so using FDA's existing processes to request additional information through interactive review or through a deficiency letter.[85] If deficiencies with the PCCP remain unresolved, FDA may authorize the device upon withdrawal of the PCCP.

As described in Section V.C. of this guidance, a copy of the PCCP with a title and version number should be included in the marketing submission for the device. If the PCCP is revised during review, such as in response to deficiencies, a final, revised version of the PCCP should be submitted as a clean copy to FDA and should include a title and current version number for the PCCP. FDA authorizes the PCCP as part of the marketing authorization of a device. To that end, the PCCP will be referenced in the device's letter of authorization, including the PCCP title and version number.

---

[79] E.g., through a PMA supplement or a traditional 510(k) for a device that has already been authorized.
[80] See, e.g., 21 CFR 807.87, 21 CFR 860.220, or 21 CFR 814.20. In cases where the modified PCCP is the reason for the marketing submission, and in general, manufacturers may provide references in the marketing submission to prior marketing submissions for content that remains unchanged, as appropriate.
[81] Sections 515C(a)(2) and 515C(b)(2) of the FD&C Act.
[82] See FDA's guidance "The Special 510(k) Program."
[83] Note that "focus of the review" is not intended to imply a review of the PCCP only; rather, the focus on the PCCP is as a significant change to the device that could affect the safety or effectiveness of the device.
[84] See, e.g., sections 513(i)(1) and 515C of the FD&C Act and 21 CFR 860.7.
[85] See FDA's guidance, "Developing and Responding to Deficiencies in Accordance with the Least Burdensome Provisions."

Over time, as a manufacturer implements their authorized PCCP for their device, they may wish to make modifications to the device that are not included in their authorized PCCP and that may require a new marketing submission.[86] If prior modifications have been implemented consistent with the authorized PCCP for the device, the new marketing submission for the device should include a summary of those modifications that have been implemented. This may include information in the device description, labeling, and other relevant sections of the marketing submission so that FDA can understand the current device characteristics and performance. In the context of premarket authorization, FDA does not intend to re-review the adequacy of modifications implemented consistent with an authorized PCCP; however, FDA needs to have an understanding of the current device characteristics and performance in order to make a determination[87] of reasonable assurance of safety and effectiveness or substantial equivalence. Additionally, if the manufacturer would also like to modify their previously authorized PCCP, the marketing submission must include the appropriate marketing submission requirements[88] and the proposed, modified PCCP for the device (see Section V.E. of this guidance).[89]

For devices subject to 510(k) requirements, a manufacturer must compare their subject device to the version of the predicate device cleared or approved prior to changes made under the PCCP.[90] However, once a 510(k) for a device that includes modifications that have been implemented consistent with the authorized PCCP has been cleared in a subsequent marketing submission, such device can now serve as an eligible predicate device.

VI. Description of Modifications

A description of each planned modification to an AI-DSF should be included in the Description of Modifications section of a PCCP. The detailed description should describe specific changes to the device characteristics and performance resulting from implementation of the modifications. To ensure an efficient review, FDA recommends that a PCCP include only a limited number of modifications that are specific, and that can be verified and validated.

The Appendices provide key information to help manufacturers implement the recommendations in this guidance, including example modifications, as well as scenarios for employing an authorized PCCP for an AI-DSF (Appendix B).

A. Goals of the Description of Modifications Section

A dedicated Description of Modifications section in a PCCP should identify the specific, planned modifications to the AI-DSF that the manufacturer intends to implement. The Description of Modifications should include the specifications for the characteristics and performance of the

[86] 21 CFR 807.81(a)(3) and 21 CFR 814.39(a).
[87] See, e.g., sections 513(i)(1) and 515C of the FD&C Act and 21 CFR 860.7.
[88] See, e.g., 21 CFR 807.87, 21 CFR 860.220, or 21 CFR 814.20. In general, manufacturers may provide references in the marketing submission to prior marketing submissions for content that remains unchanged, as appropriate.
[89] Sections 515C(a)(2) and 515C(b)(2) of the FD&C Act.
[90] Section 515C(c) of the FD&C Act.

device that, following the agreed upon verification and validation described in the Modification Protocol, can be implemented without a new marketing submission.

B. Content of the Description of Modifications Section

To achieve these goals, FDA recommends that the Description of Modifications enumerate the list of individual proposed device modifications discussed in the PCCP, as well as the specific rationale for the change to each part of the AI-DSF that is planned to be modified. In some situations, a Description of Modifications may consist of multiple modifications. It may be helpful to reference the labeling sections that are anticipated to be impacted for each modification in the Description of Modifications section (such labeling changes for each modification should be included in the Modification Protocol, as described in Section VII.B.(4) of this guidance).

FDA recommends that a PCCP include modifications that are specific, and that can be verified and validated. Modifications should also be presented at a level of detail that permits understanding of the specific modifications that will be made to the AI-DSF. Each modification should be linked to a specific performance evaluation activity within the Modification Protocol (for an example, see Table 1 in Section VII.C. of this guidance).

The Description of Modifications should clearly state if the planned modifications are proposed to be implemented automatically (i.e., whether the modifications are implemented automatically by software), whether modifications are implemented manually (i.e., involving steps that require human input, action, review, and/or decision-making, and therefore are not implemented automatically), or a combination of both. Understanding that this is an evolving area, FDA will consider PCCPs for AI-DSFs for modifications that are implemented automatically. The Agency recognizes that this subset of AI-DSFs has an additional degree of complexity; as with all AI-DSFs, FDA will consider the benefit-risk profile of the specific device that is the subject of the PCCP, the specific modifications being proposed, and FDA's experience applying this policy when reviewing PCCPs containing automatically implemented modifications. To help facilitate FDA's review in this regard, it may be helpful for manufacturers to clearly establish boundaries or guardrails that define the range of automatic updates. Because this guidance includes recommendations for PCCPs for AI-enabled devices broadly, FDA recommends manufacturers discuss considerations for automatic updates for AI-DSFs through the Q-Submission Program.

The Description of Modifications should also clearly specify if the proposed modifications will be implemented in a uniform manner across all devices on the market (sometimes referred to as homogenous or global changes, or global adaptations), and/or implemented differently on different devices on the market based on, for example, the unique characteristics of a specific clinical site or individual patients (sometimes referred to as heterogenous or local changes, or local adaptations). For local adaptations, the Description of Modifications should include a description of what local factors or conditions warrant a local change. In addition, the Description of Modifications should also include information regarding the expected frequency of updates. This could range from modifications being implemented, for example, annually, or less frequently, resulting in a device that is primarily 'locked', to modifications being implemented continuously as the device adapts to new data during its use. Finally, it may be

helpful to reference the labeling sections that are anticipated to be impacted for each local adaptation in the Description of Modifications section (such labeling changes for each local adaptation should be included in the Modification Protocol, as described in Section VII.B.(4) of this guidance).

C. Types of Modifications

Modifications that are appropriate for inclusion in a PCCP include those that are intended to maintain or improve the safety or effectiveness of the device. Modifications should also be specific, and should be able to be verified and validated. Types of modifications that may be appropriate for inclusion in a PCCP include:

(i) modifications related to quantitative measures of AI-DSF performance specifications;
(ii) modifications related to device inputs to, and compatibility with, the AI-DSF; and
(iii) certain modifications related to the device's use and performance (e.g., for use within a specific subpopulation).

Modifications related to quantitative measures of AI-DSF performance specifications include improvements to analytical and clinical performance resulting from re-training the AI model based on new data within the intended use population from the same type and range of input signal.

Modifications related to device inputs for the AI-DSF may include changes to data type specifications to include new sources of the same input signal type (e.g., different makes, models, or versions of a data/imaging acquisition system), or limited modifications related to new types of inputs (e.g., adding new data inputs, such as age, or transforming data inputs, such as through data normalizations). Modifications related to input sources and compatibility with the AI-DSF may include updates related to available, compatible software or hardware and device interoperability (e.g., different compatible hardware, updated operating systems, or updated cloud infrastructure).

Modifications related to the device's use and performance could include authorization of a device for a specific subpopulation within the originally indicated population based on re-training on a larger data set for that subpopulation that was not previously available. See Appendix B for examples of modifications included in an authorized PCCP for various AI-DSFs.

Modifications included in a PCCP must maintain the device within the device's intended use,[91] and as applicable, must allow the device to remain substantially equivalent to the predicate device.[92] In general, FDA believes that modifications included in a PCCP should also maintain the device within the device's indications for use.[93] As with modifications to the intended use, FDA believes that most modifications to the indications for use included in a PCCP would be

[91] See sections 515C(a)(2) and 515C(b)(2) of the FD&C Act.
[92] Section 515C(b)(2)(B) of the FD&C Act.
[93] FDA has a long-standing policy of applying the definition of indications for use in the PMA regulation at 21 CFR 814.20(b)(3)(i) in the same way in the 510(k) context. See the FDA guidance "The 510(k) Program: Evaluating Substantial Equivalence in Premarket Notifications [510(k)]."

difficult for FDA to assess prospectively to determine whether the device would remain safe and effective.[94] However, there may be certain modifications to the indications for use (e.g., certain changes in the indications for use to specify use of the device with an additional device or component) that may be appropriate for inclusion in a PCCP. FDA highly encourages manufacturers to discuss modifications to the indications for use that may be included in a proposed PCCP with the appropriate FDA review division through the Q-Submission Program.

Recognizing there is a spectrum of risk for devices, for the purposes of reviewing a PCCP, FDA intends to, among other considerations, take into account the benefit-risk profile of the specific device that is the subject of the PCCP, the specific modifications being proposed, and FDA's experience applying this policy across different device types. As such, certain modifications that may be appropriate for inclusion in a PCCP for one device may not be appropriate for inclusion in a PCCP for another device. Some modifications may not be appropriate for inclusion within a PCCP for any device. FDA encourages manufacturers to leverage the Q-Submission Program to obtain FDA feedback on a proposed PCCP prior to submitting a marketing submission.

VII. Modification Protocol

The Modification Protocol should include the documentation describing the methods that will be followed when developing, validating, and implementing modifications delineated in the Description of Modifications section of the PCCP. The Modification Protocol should also include the verification and validation activities, including pre-defined acceptance criteria, for those modifications, and a step-by-step delineation of how those modifications will be implemented while assuring the device remains safe and effective. The Appendices provide key information to help manufacturers implement the recommendations in this guidance, including detailed questions and considerations for the recommended content of a Modification Protocol in a PCCP (Appendix A).

Documentation of modifications verified and validated per the Modification Protocol must be compliant with the QSR, including that the manufacturer must document the change in accordance with the manufacturer's quality system.[95] The QSR requires manufacturers of finished medical devices to review and approve modifications to device design and production (21 CFR 820.30(i) and 820.70(b)) and document changes and approvals in the device master record (21 CFR 820.181).

A. Goals of the Modification Protocol Section

Whereas the Description of Modifications outlines the planned modifications to an AI-DSF, the Modification Protocol should describe the methods that will be followed when developing, validating, and implementing those modifications, to ensure the device remains safe and effective. The methods described in the Modification Protocol should be consistent with and support the modifications outlined in the Description of Modifications.

[94] Sections 515C(a)(2) and 515C(b)(2)(A) of the FD&C Act.
[95] 21 CFR Part 820.

The goals of the Modification Protocol are to:

- Identify the appropriate and applicable data, test methods, analysis methods, and specified acceptance criteria used to develop, validate, and implement all proposed modifications;
- Identify the update process, and as appropriate, the plans for communication and/or training for users for implemented modifications;
- Ensure that the information that would otherwise be generated and submitted to FDA (i.e., if the modifications were implemented on a device that did not have an authorized PCCP) will be generated by the manufacturer for each modification and maintained consistent with recordkeeping requirements and in accordance with the manufacturer's quality system;[96]
- Ensure that anticipated risks and risk mitigation strategies have been identified and included in the Impact Assessment; and
- Be least burdensome[97] for the manufacturer to develop and for FDA to review. This includes being traceable (so that modifications in the Description of Modifications can be traced to verification and validation activities in the Modification Protocol) and specific (so that the PCCP is sufficiently comprehensive to support the proposed modifications).

This guidance identifies four primary components of a Modification Protocol that outline a manufacturer's 1) data management practices, 2) re-training practices, 3) performance evaluation protocols, and 4) update procedures, including communication and transparency to users and real-world monitoring plans as applicable, for each modification in a PCCP. In FDA's experience, these four components should generally provide FDA with the information needed to evaluate the PCCP. For a particular marketing submission, additional components of a Modification Protocol may need to be included to assist in FDA's determination of substantial equivalence or reasonable assurance of safety and effectiveness of the device and PCCP.

Manufacturers should follow their risk management processes to develop a Modification Protocol that considers each modification.[98] In some cases, the same methods in each component of the Modification Protocol may support all modifications in a PCCP for a device. In other cases, the same methods in each component of the Modification Protocol may not be adequate for every modification in a PCCP. For each planned modification provided in the Description of Modifications, FDA recommends that each of the four primary components of a Modification Protocol be addressed. The Modification Protocol should include a description of how its proposed methods are similar to or different from methods used elsewhere in the marketing submission. For example, if the validation methods in the Modification Protocol represent a subset of the original testing for the device, or if the acceptance criteria for the validation are different, manufacturers should describe these differences and provide a justification. The justification for a different methodology may include references to other marketing submissions where the methodology was used for similar modifications.

---

[96] 21 CFR Part 820.
[97] See FDA's guidance "The Least Burdensome Provisions: Concept and Principles."
[98] See AAMI CR 34971:2022, AAMI Consensus Report – Guidance on the Application of ISO 14971 To Artificial Intelligence And Machine Learning.

B. Content of the Modification Protocol Section

To achieve these goals, FDA recommends that a Modification Protocol outline the methods for each component described below. Example elements of each of the four Modification Protocol components are provided in Appendix A.

(1) Data management practices

What they are: AI-DSF training, tuning, and testing typically utilize data that include the inputs (e.g., medical images) that will be used by the device and often utilize a label or 'reference standard' that is determined through a reference standard determination and/or an annotation process. Training data, tuning data, and test data are sequestered (i.e., the test data set is a unique data set compared to the data set used for training and tuning) to prevent overfitting and misquotes of test performance. The training, tuning, and testing methods aim to identify and mitigate unwanted bias in the data (which may be inherent from historical datasets) and to improve the robustness and resilience of these algorithms to withstand changing clinical inputs and conditions. Additional methods to mitigate bias may be helpful, such as cross-validation, bootstrapping, bagging, ensembling, and the use of synthetic or augmented data. To support modifications to an AI-DSF that may need training, tuning, and/or testing, it is anticipated that new data (i.e., data that were not used to develop the initial AI-DSF) will be collected. The data management practices in a Modification Protocol should outline how those new data will be collected, annotated, curated, stored, retained,[99] controlled, and used by the manufacturer for each modification. The data management practices in a Modification Protocol should also clarify the relationship between all of the data used to train, tune, and test the initial and any subsequent versions of the AI-DSF. It should also describe the control methods employed to ensure that the data used to test the AI-DSF is separate and independent from the development process used to train and tune the AI-DSF.

Why they are recommended: This information allows FDA to understand the manufacturer's data management practices that will be used to support each modification to an AI-DSF, including 1) how the manufacturer plans to obtain and use training, tuning, and test data that are complete and representative of the proposed intended use populations (e.g., with respect to race, ethnicity, disease severity, sex, age, etc.[100]) and intended environments; 2) whether identifiable subpopulations will be adequately represented, including intersectional groups, and separated into training, tuning, and test sets to minimize AI model bias; 3) how training, tuning, and test data will be sequestered to prevent overfitting and misquotes of test performance; 4) how older data will be complemented or replaced by newer data so that the performance is representative of the current patient population and standard of care; 5) whether the reference standard represents the best available process for determining the ground truth; and 6) how the data management practices may reduce the potential to produce discriminatory outcomes. A clear explanation of data management practices also provides assurance to FDA that modifications to the AI-DSF are

[99] The QSR requires manufacturers to retain all records for a period of time equivalent to the design and expected life of the device, but in no case less than 2 years from the date of release for commercial distribution by the manufacturer (21 CFR 820.180(b)).

[100] We recommend that manufacturers consider additional characteristics, such as race, color, ethnicity, sex (including pregnancy, childbirth, and related medical conditions), religion, age, national origin, disability, veteran status, and genetic information.

based on data that are representative of the device's indications for use. This includes information regarding products that will be used to generate data as inputs for the AI-DSF, patient populations in which the device will be used, and clinical scenarios where the device will be used.

What manufacturers should include in a submission: Examples of the types of information manufacturers should provide in a Modification Protocol describing their data management practices are provided in Appendix A. In general, this information should describe: how data will be collected, including clinical study protocols with inclusion/exclusion criteria; information on how data will be processed, stored, and retained;[101] the process that will be followed to determine the reference standard; when clinician interpretation is used for determining the reference standard (representing the ground truth), a protocol describing how the reference standard is determined; the quality assurance process related to the data; the data sequestration strategies that will be followed during data collection to separate the data into training, tuning, and test sets; and the protocols in place to prevent access during the training, tuning, and testing process to data intended for performance testing.

(2) Re-training practices

What they are: AI software generally involves multiple processing steps from the point the AI-DSF receives the input data to the point it provides an output. The re-training practices component of a Modification Protocol should identify the processing steps that are subject to change for each modification and the methods that will be used by the manufacturer to implement modifications to the AI-DSF. In addition, if re-training involves architecture modifications (e.g., in a neural network, modifications to training hyperparameters or the number of nodes, layers, etc.), the re-training practices component of a Modification Protocol should also describe the rationale or the justification for each specific architecture modification.

Why they are recommended: Information on the manufacturer's re-training practices allows FDA to understand how the proposed modifications will be achieved through re-training, to determine if modifications are implemented following appropriate, well-defined practices,[102] and to determine if the performance evaluation and update procedures (discussed below) support the modifications. Information on the manufacturer's re-training practices is typically provided in the "device description" of a marketing submission for the majority of AI-DSFs that FDA reviews. The specifics of what should be included in this component of the Modification Protocol will depend on the type of modification and specific device.

What manufacturers should include in a submission: Examples of the types of information manufacturers should provide in a Modification Protocol describing their re-training practices are provided in Appendix A. In general, this information should identify the objective of the re-training process, provide a description of the AI model, identify the device components that may be modified, outline the practices that will be followed (e.g., data sequestration strategies during

---

[101] See 21 CFR 820.180(b).
[102] For example, FDA has published a document on "Good Machine Learning Practice for Medical Device Development: Guiding Principles."

re-training), and identify any triggers for re-training (e.g., when the quantity of new data reaches a certain size or when a drift in data is observed over time).

(3) Performance evaluation

What they are: FDA may require that performance requirements for changes made under the plan be provided in a PCCP.[103] Performance evaluation methods should describe the processes that will be followed to verify and validate that the modified AI-DSF will meet the specifications identified as part of a specific modification, in addition to maintaining the specifications that are not part of the modification, but may be impacted by the modification. Performance evaluation should include, as applicable, the plans for verification and validation of the entire device following the implementation of each individual modification and in aggregate for the planned modifications. This includes, but is not limited to, AI model testing protocols comparing the newly modified device to both the original device (the version of the device without any modifications implemented) and the last modified version of the device. For example, for device software functions that drive hardware functionality, performance evaluation should include not only the device software functions, but also the effect of the modifications on hardware functionality. The content of this section in a Modification Protocol should provide details on the study design, performance metrics, pre-defined acceptance criteria, and statistical tests for each planned modification. More comprehensive testing can potentially support a broader set of proposed modifications.

Why they are recommended: Information regarding the manufacturer's performance evaluation methods allow FDA to confirm that appropriate study designs, including performance metrics and statistical tests, will be used to evaluate the effect of modifications on overall device performance. Performance evaluation of the device is important to ensure that specified acceptance criteria for all proposed modifications will continue to be met for the device's specifications.

What manufacturers should include in a submission: Examples of the types of information manufacturers should provide in a Modification Protocol describing their performance evaluation are provided in Appendix A. In general, this information should describe how performance evaluation will be triggered; how sequestered test data representative of the clinical population and intended use will be applied for testing; what performance metrics will be computed; and what statistical analysis plans will be employed to test hypotheses relevant to performance objectives for each modification. The Modification Protocol should also affirmatively state that if there is an unresolvable failure in performance evaluation for a specific modification (e.g., the predefined acceptance criteria for a specific modification are not met), the failure(s) will be recorded, and the specific modification(s) will not be implemented. A failure would not be considered to be unresolvable if a root cause analysis of the failure reveals it is not related to specific aspects of the PCCP, and in such cases, performance testing may be conducted again.

---

[103] See sections 515C(a)(3), 515C(b)(3), and 513(f)(2) of the FD&C Act.

(4) Update procedures

What they are: Data management practices, re-training practices, and performance evaluation described above largely relate to making and testing modifications to the AI-DSF. Once these meet the performance objectives, manufacturers will need to update the AI-DSF to implement the modifications and communicate information to users about the modifications that is needed to safely use the device. The update procedures should clearly describe which planned modifications will be implemented automatically, implemented manually, or a combination of both, and how the manufacturer plans to communicate this information to users. The update procedures in a Modification Protocol should describe how manufacturers will update their devices to implement the modifications consistent with QSR, and, if appropriate for such modifications, provide appropriate transparency to users and updated user training.[104] The manufacturer should also describe their post-market surveillance plans and procedures, which may include real-world monitoring and notification requirements if the device does not function as intended pursuant to the authorized PCCP.[105] As part of a manufacturer's responsibility to ensure that devices remain safe and effective, FDA anticipates that manufacturers will monitor their device's safety (e.g., adverse events) and effectiveness (e.g., performance) over time as modifications are implemented consistent with their authorized PCCP. Monitoring activities may differ for AI-DSFs for which updates are deployed manually compared to automatically, or for which updates are deployed globally or locally.

The update procedures in a Modification Protocol should also address how labeling will be updated when modifications are implemented, as appropriate.[106] It should also include a description of the labeling sections that are anticipated to be impacted by the implementation of the modifications. The available labeling must include adequate directions for use.[107] The available labeling should also reflect information about the current version(s) of the AI-DSF available to the user, including information regarding site-specific modifications. New unique device identifiers (UDIs) are required for devices that are required to bear a UDI on its label when there is a new version and/or model, and for new device packages.[108] FDA recommends that the labeling not include information on modifications to the AI-DSF that have not been implemented in the available version because it could cause confusion and would be misleading. Additionally, if the labeling states that a modification to the AI-DSF has been implemented when it has not, the device might be deemed misbranded.[109]

Why they are recommended: Information on the manufacturer's update procedures allows FDA to understand 1) how risks from implementing modifications may be mitigated by the update process; 2) how communication regarding the device updates will be provided to users (e.g., so that updates in device output results will be correctly interpreted by users); 3) how the device

---

[104] See sections 515C(a)(3), 515C(b)(3), and 513(f)(2) of the FD&C Act.
[105] See sections 515C(a)(3), 515C(b)(3), and 513(f)(2) of the FD&C Act.
[106] See Section V.C. of this guidance for the recommendations regarding information about the PCCP that should be included in the device labeling.
[107] See 21 CFR 801.5(a), requiring that labeling include adequate directions for use including statements of all conditions, purposes, or uses for which the device is intended.
[108] See 21 CFR 830.50.
[109] See section 502(a)(1) of the FD&C Act, stating that a medical device is deemed misbranded if its labeling is false or misleading in any particular.

operation will remain safe and effective after the update; and 4) how all users will be kept up-to-date about device functionality and performance. In addition, it is important for FDA to understand how potential risks associated with the update process, itself, may be mitigated.

What manufacturers should include in a submission: Examples of the types of information manufacturers should provide in a Modification Protocol describing their update procedures are provided in Appendix A. In general, this information should include 1) confirmation that the verification and validation plans for the modified version of the device are the same as those that have been performed for the version of the device prior to the implementation of the modifications, or identification of any differences between the two plans; 2) a description of how software updates will be implemented; 3) a description of how legacy users will be affected by the software update (if applicable); and 4) a description of how modifications will be communicated to the users, including transparency on any differences in performance or device inputs, and/or known issues that were addressed in the update. Communication of performance changes should be consistent with performance evaluation described in the Modification Protocol.

C. Traceability Between the Description of Modifications Section and the Modification Protocol Section

The PCCP should clearly delineate which parts of the Modification Protocol are applicable to each modification within the Description of Modifications. For a PCCP with multiple modifications, this may be accomplished through a traceability table; a sample traceability table is provided below in Table 1. This sample traceability table provides an example of how a manufacturer can depict the traceability between the Description of Modifications and Modification Protocol, as well as how to provide clear references to where within the PCCP this information is located in a marketing submission. In other words, a traceability table can help to identify where each method supporting each modification may be found in the marketing submission.

Table 1. Example of Description of Modifications to Modification Protocol Traceability Table

	Modification Protocol Component			
Modification	Data management practices	Re-training practices	Performance evaluation	Update procedures
Modification #1	*Method A (see Section X.A)*	*Method D (see Section X.D)*	*Method G (see Section X.G)*	*Method J (see Section X.J)*
Modification #2	*Method A (see Section X.A)*	*Method E (see Section X.E)*	*Method H (see Section X.H)*	*Method J (see Section X.J)*
Modification #3	*Method B (see Section X.B)*	*Method F (see Section X.F)*	*Method I (see Section X.I)*	*Method J (see Section X.J)*

VIII. Impact Assessment

An Impact Assessment in a PCCP is the documentation of the assessment of the benefits and risks of implementing a PCCP for an AI-DSF, as well as the mitigations of those risks. The manufacturer's existing quality system should be used as the framework in which to conduct an Impact Assessment for the modifications set forth in the PCCP.

Documentation for an Impact Assessment provided to FDA in a marketing submission containing a PCCP should:

1) Compare the version of the device with each modification implemented individually to the version of the device without any modifications implemented;
2) Discuss the benefits and risks, including risks of harm[110] and unintended bias, of each individual modification;
3) Discuss how the verification and validation activities proposed within the Modification Protocol continue to reasonably ensure the safety and effectiveness of the device;
4) Discuss how the implementation of one modification impacts the implementation of another; and
5) Describe the cumulative impact of implementing all modifications.

FDA believes it is important to address these elements in an Impact Assessment in order to demonstrate that the combination of the proposed modifications is unlikely to introduce additional, unmitigated risks, and that the safety and effectiveness of the device is maintained as modifications are implemented.

Impact Assessment documentation for a PCCP in a marketing submission should discuss how the individual modifications included in the PCCP impact not only the AI-DSF, but also how they impact the overall functionality of the device, including how they impact other device software functions and/or device hardware. For combination products, such documentation should also discuss how the individual modifications included in the PCCP for the device constituent part impact the biologic and/or drug constituent part, and the combination product as a whole. Additionally, if the modifications in a PCCP are intended to affect any device function(s) of a multiple function device product, we recommend considering FDA's guidance "Multiple Function Device Products: Policy and Considerations." In particular, as it pertains to the AI-DSF and the PCCP, it is important to determine if any information should be included in the Impact Assessment in a PCCP in a marketing submission so that FDA may assess the impact of the "other function(s)" on the safety or effectiveness of the AI-DSF as it is modified consistent with the PCCP.

Some information related to the Impact Assessment may be included elsewhere in the marketing submission, for example in sections for the risk assessment for the device or the Modification Protocol in the PCCP. As such, FDA recommends providing clear references in the Impact Assessment section of the PCCP to the relevant sections in the marketing submission that support the Impact Assessment.

---

[110] See, e.g., harm, as defined in ISO 14971 *Medical devices – Application of risk management to medical devices*, is the physical injury or damage to the health of people.

Appendix A: Example Elements of Modification Protocol Components for AI-DSFs

In general, a Modification Protocol that is included as part of a PCCP in a marketing submission should include four components that outline a manufacturer's 1) data management practices, 2) re-training practices, 3) performance evaluation protocols, and 4) update procedures, for each modification in the Description of Modifications for the AI-DSF. However, manufacturers may include other or additional components if they believe that their proposed protocols do not fit into any of these four components. To help illustrate the level of detail and additional information that may be helpful to include in a marketing submission, this appendix includes examples of questions for consideration and the types of information manufacturers should provide in the components of a Modification Protocol. In thinking about these questions, manufacturers should consider the intended use populations (e.g., with respect to race, ethnicity, disease severity, sex, age, or others, as appropriate) and intended environments.

Note that this is a developing area, and as FDA gains experience, these example questions may change. The items below are not an exhaustive list of topics that a manufacturer is expected to cover, and all questions may not apply to all marketing submissions. The topics and questions are provided to assist in identifying the types of information that may be helpful to provide to FDA to appropriately describe the components of a Modification Protocol. The appropriate information to provide will vary by the AI-DSF and Modification Protocol.

In certain circumstances, FDA may request additional Modification Protocol components or information to be included in a PCCP for some device types so that FDA can make a determination of reasonable assurance of safety and effectiveness or substantial equivalence when reviewing the device and PCCP.[111] Additionally, some sections of a Modification Protocol may be more or less detailed depending on the complexity and risks of each modification in the PCCP. Overall, FDA recommends that manufacturers consider the topics and questions in this appendix for their particular AI-DSF and PCCP as a means of encouraging a detailed and methodically prepared PCCP.

(1) Data Management

Different data can be collected and used for training, tuning, and testing AI model updates. In cases where manufacturers are collecting new training, tuning, and test data, the Modification Protocol should include how the data will be used (e.g., for AI model development or testing), and how the data management supports these uses.

a. Collection protocols

1.a.1. For each modification, what are the inclusion/exclusion criteria for data collection, and how are they linked to the intended use population?

1.a.2. What is the intended distribution of the data set along covariates describing the patient population (e.g., sex, age, race, height, weight, disease conditions) and

[111] Such information would be required in the marketing submission pursuant to e.g., sections 513(i)(1) and 515C of the FD&C Act, and 21 CFR 807.81(a)(3), 21 CFR 814.39(a), 21 CFR 860.7.

data acquisition conditions (e.g., sites, data acquisition devices/methods, imaging and reconstruction protocols)? Is this distribution representative of the intended use population, including intersectional groups?

1.a.3. Will the data be collected prospectively or retrospectively? Will the data set include consecutive cases within a given date range? Otherwise, if random sampling is planned, what method or technique will be used and how will it account for bias and randomness?

1.a.4. Are there any plans for enrichment or stratified sampling, such as specific patient subgroups (e.g., sex, age, race), relevant disease subtypes (e.g., genotypic or phenotypic variants, disease burden, severity, or presentation, etc.), and/or variations in care delivery (e.g., inpatient/outpatient care settings)?

1.a.5. What is the number and geographical distribution of data collection sites?

1.a.6. What are the measures to mitigate potential unwanted bias in learning or performance estimation, for example, due to issues related to new training, tuning, or test data, respectively?

1.a.7. What are the strategies and measures to understand and mitigate potential biases in the data, such as those due to historical inequalities to medical treatment access by different populations?

1.a.8. What are the strategies to ensure data sets remain relevant over time with respect to changes in, for example, data acquisition technologies or protocols, clinical practice, patient populations, and disease conditions?

1.a.9. Are data collection, storage, retention, and use protocols in compliance with regulations for human subject protections and requirements for clinical investigations (e.g., pursuant to 21 CFR Part 812, 45 CFR Part 46, 21 CFR Part 50, and 21 CFR Part 56, as applicable)?

1.a.10. For AI-DSFs that use input data from dedicated acquisition systems (e.g., software device functions in a patient monitor that uses connected sensors), are the data acquired with the systems and settings with which the AI-DSF will be used? For device software functions that use input data from different acquisition systems (e.g., interoperable medical devices), do the data acquired meet the input specifications of the AI-DSF?

b. Assurance of data quality

1.b.1. What processes are in place to ensure data consistency and completeness are maintained during data collection for training, tuning, and test data?

1.b.2. What are the strategies used to promote data authenticity, transparency, and integrity?

1.b.3. How will potentially missing data elements within a case/record be handled for training, tuning, and test data?

1.b.4. Are there criteria for including/excluding cases/records based on data quality (in addition to inclusion/exclusion criteria listed in 1.a.1. above), and if so, what are the criteria and rationale?

1.b.5. If data might be excluded as a result of the quality assurance process, what methods are planned to minimize the impact on the generalizability of training and accuracy of testing?

1.b.6. What are the traceability methods or strategies that will be used to identify and investigate data issues?
1.b.7. Will data that are obsolete or no longer needed be removed? If so, what are the criteria for data removal, and what strategies will be used to appropriately manage data removal in a consistent manner?
1.b.8. What controls are in place to prevent and identify unauthorized access or manipulation of the training, tuning, and test data sets? For example, what controls are in place to prevent malicious addition or deletion of data for the purpose of impacting model performance, introducing bias, or overtraining on repeated data, among others?
1.b.9. Is there an automated process to ensure data quality, and if so, what is the process?

c. Reference standard determination

For purposes of this guidance, the reference standard is the most suitable standard to define the true condition for each patient/case/record. The PCCP should include a rationale for the determination of the "most suitable standard," which may include consideration of device risk. The reference standard may be used during training, tuning, testing, or all three.

1.c.1. What is the justification for the method for the determination of the reference standard?
1.c.2. If the reference standard is based on evaluations from clinicians, what was the grading protocol used (e.g., what is the total number of clinicians who participated and their qualifications; what data are these clinicians provided with; and how are the clinicians' evaluations collected/adjudicated for determining the reference standard)?
1.c.3. What is the strategy for addressing cases where results obtained using a reference standard may be equivocal or missing?
1.c.4. What is the uncertainty inherent in the reference standard?
1.c.5. Will any of the methods for determining the reference standard be automated? Are there differences between the reference standards used for training/ tuning/testing?
1.c.6. Are there differences between the reference standard used to support the initial AI-DSF marketing authorization and the reference standard being applied to update the AI model?

d. Sequestration of test data sets

For purposes of this guidance, sequestration of test data sets means that manufacturers do not have access to the test data set for the purpose of AI-DSF development.

1.d.1. What strategies will be employed at the outset of data collection to shield the test data set from the AI-DSF development?
1.d.2. What are the specific procedures to be followed so that the test data set remains sequestered during re-training?
1.d.3. If test data are planned to be used multiple times for performance evaluation, what measures are in place to prevent unwanted bias from being introduced through AI

model manufacturers learning substantial information about the test data set and results?

1.d.4. What are the descriptive statistics (e.g., covariate range, mean, median) for each data set and how similar are they to those for the intended use population?

(2) Re-Training

a. Re-training objectives and focus

2.a.1. How are the modifications presented in the Description of Modifications in the PCCP related to the planned re-training methods?

2.a.2. Which parts of the AI-DSF are planned to be modified (e.g., transfer learning, data pre-processing, data augmentation, only a certain set of coefficients, architecture and hyperparameters, loss functions, optimization methods and criteria, types of AI model inputs and outputs), and what are the details of the planned modifications to the AI-DSF design? What is the specific rationale for the change to each part that is planned to be modified?

2.a.3. For each part of the AI-DSF that will be modified, is AI model re-training needed to achieve the modifications specified in the PCCP?

2.a.4. If re-training applies to only certain parts of the AI-DSF, what are the plans to ensure that other functions or software components are not affected?

b. Re-training implementation:

2.b.1. What are the triggers for re-training (e.g., when new data reaches a certain size, when a drift is observed, periodically in time)?

2.b.2. What strategies will be employed to identify and limit overfitting?

2.b.3. Are there risks related to AI model bias introduced by re-training a modified AI model, and if so, what are planned mitigations?

(3) Performance Evaluation

a. Triggers to initiate performance evaluation

3.a.1. What are the triggers for initiating performance evaluation of a re-trained AI model or modified AI-DSF (e.g., re-training shows a certain performance level on the training or tuning data, test data reaches a certain size, periodically in time)?

3.a.2. How frequently is this expected to occur?

b. Assessment metrics and elements

3.b.1. How are the plans for data management in (1) above applied to produce the test data for performance evaluation that are different from any training or tuning data?

3.b.2. What metrics will be computed to understand device performance?

3.b.3. How do these metrics demonstrate that the modified device can be safely used?

3.b.4. How will the metrics provide a comprehensive assessment of device performance and patient safety?

3.b.5. What challenging cases (i.e., cases outside the norm) or known challenging scenarios will be evaluated?

c. Statistical analysis plans

3.c.1. What is the plan for evaluating equivalent or improved performance with respect to previously validated versions, including the original version, of the AI-DSF?
3.c.2. What are the high-risk subpopulations and subgroups (e.g., acquisition protocols) that need to be evaluated?
3.c.3. How will this evaluation be used to support labeling specifications?
3.c.4. How will you test that performance in one area (e.g., sensitivity) does not result in unacceptable degrading performance in another (e.g., specificity)?
3.c.5. How will the sample size be determined?
3.c.6. Is the primary analysis based on the intention-to-diagnose population (no study subjects will be excluded) or the per-protocol population (subjects with protocol violations will be excluded)?
3.c.7. How is variability in the reference standard accounted for (e.g., in the case of reader variability when clinical interpretation is used)? When the reference standard may be imperfect (e.g., sometimes includes a diagnostic error), are errors made by the imperfect reference standard conditionally independent of errors made by the AI-DSF, or are they positively correlated?
3.c.8. How will missing data and outliers be addressed in analysis?

d. Performance targets

3.d.1. What are the acceptance criteria? If applicable, how do the acceptance criteria compare to the acceptance criteria for the authorized version of the device?
3.d.2. What clinical considerations were used to develop the acceptance criteria?
3.d.3. How will the acceptance criteria support that the modification will be successfully implemented?

e. Additional testing needs

3.e.1. Is database testing sufficient to address the risks associated with the proposed modification (e.g., does the user need to interact with the device to evaluate the performance or address a clinical risk or, for software that is part of a hardware device,[112] how is the effect of the modification on hardware functionality evaluated)?
3.e.2. How may clinical usability need to be addressed for a modification?

(4) Update Procedures

a. Software verification and validation

4.a.1. Does the proposed modification necessitate a different software verification and validation plan from that used for the version of the device without any modifications implemented?

---

[112] For purposes of this guidance, "part of a hardware device" means the software is used to control a device, or the software is necessary for a hardware device to achieve its intended use.

4.a.2. What type of testing will be performed? Does the AI-DSF need to be validated to function in an integrated environment, where interoperable, heterogeneous medical devices and other equipment are combined (e.g., when the input to the AI-DSF originates from other devices or when the output of the AI-DSF affects other devices or equipment for patient care)?
4.a.3. If the device includes other device functions in addition to the AI-DSF, how will the impact of modifications to the AI-DSF on the other device functions be evaluated?
4.a.4. If the device includes "other functions" in addition to the AI-DSF, how will the "other functions'" impact on the safety or effectiveness of the modified AI-DSF be evaluated?

b. When and how updates will be implemented

4.b.1. How will the decision be made on when to perform an update? What is the expected timeline for implementing the modifications? Is there a set frequency of updates?
4.b.2. When and how will an update be implemented (e.g., automatically when the device is not being used, manually by users or hospital technicians, or both manually and automatically)? What is the basis on which the mechanism of implementation is dependent?
4.b.3. For AI-DSF updates to reusable medical equipment, how will the device operation, including function of critical safety features (e.g., medical device alarms), be verified following the update?
4.b.4. Will updates be made globally (i.e., the same update applied to all devices in the field) or locally (e.g., the devices may be modified for a patient/provider/care unit/hospital)?
4.b.5. What cybersecurity risk management and validation processes[113] are used in accordance with the documentation and processes provided for Section VI.B and Appendix 1.H of the FDA guidance "Cybersecurity in Medical Devices: Quality System Considerations and Content of Premarket Submissions"?

c. Communication and transparency to users

4.c.1. How will the PCCP be described in the public summary document and/or labeling?
4.c.2. How will updates be communicated to users, including, but not limited to, in updated labeling (e.g., release notes)?
4.c.3. What information about modifications to the device (e.g., performance) will be communicated to the user?
4.c.4. How will version information be presented to the user when reviewing device outputs?
4.c.5. Will users have the option to review labeling before implementing an update or be provided the option to opt out of the update?

[113] For recommendations related to cybersecurity, please consult FDA guidance documents on this topic, including "Cybersecurity in Medical Devices: Quality System Considerations and Content of Premarket Submissions."

4.c.6. How will any known biases or other performance issues with the potential to result in individual or unintended bias be disclosed, including, but not limited to, in labeling?
4.c.7. How will any changes in performance related to known issues or sources of bias be communicated to the user, including, but not limited to, in labeling?
4.c.8. What information about the population and methods for validation will be provided?
4.c.9. If patient data from previous device use is available and can be rerun on an updated AI model, will this activity be performed for the available data and will those updated results be available to patients and users? Is there a plan to communicate if patient results before and after an update would provide clinically meaningful differences?

d. Device monitoring plan

4.d.1. How will adverse events be tracked for different updates?
4.d.2. Is there a risk-based plan to monitor real-world device performance (beyond general controls) and, if not, why is it not necessary? In an effort to be transparent, will a monitoring plan be provided to the users on the performance of the AI-DSF post implementation?
4.d.3. To be transparent for users, will information about monitoring real-world device performance be provided (e.g., to inform users about the safety performance of the device following implementation of modifications), and if not, why is it not necessary?
4.d.4. How will changes in safety (e.g., adverse events, identification of new or previously unknown risks) and effectiveness (e.g., performance, identification of previously unknown biases) for manually or automatically deployed modifications be monitored, and with what frequency?
4.d.5. How will changes in performance in patient subpopulations be identified?
4.d.6. What will be the response to the identification of previously unknown risks or previously unrecognized high-risk patient subpopulations?
4.d.7. What is the strategy to respond to unexpected performance deficiencies or other hazards, or to higher levels of adverse events, as compared with previous iterations of the device?
4.d.8. How will errors in diagnosis (i.e., misdiagnosis), attributable or partially attributable to the device that do not meet the criteria for an adverse event, be tracked?
4.d.9. Will there be criteria and/or a plan to roll-back an update to reset devices to a previous version, if applicable?

Appendix B: Example AI-DSF Scenarios Employing PCCPs

The examples in this appendix illustrate different AI-DSF scenarios where a PCCP could be employed. Due to the complexity of AI-DSFs, all examples are hypothetical and do not reflect any specific authorized device.

Each example begins with a brief description of an authorized device, its intended use, and one summary of a modification from the Description of Modifications in its authorized PCCP (in the examples, denoted as "Brief Overview of Pre-Specified Modification"). Please note that the provided summaries of the devices and modifications in this appendix are *not* intended to reflect the complete content or detail expected in a Description of Modifications section in a PCCP. Rather, proposed modifications should be described in much greater detail in a PCCP, consistent with the recommendations provided throughout this guidance. The post-authorization modification scenarios are described to illustrate how the PCCP would be implemented. As described in Section V.D. of this guidance, FDA considers a modification to be consistent with the authorized PCCP when the modification has been specified in the Description of Modifications included in the PCCP *and* has been implemented in accordance with the Modification Protocol. A distinction is drawn between post-authorization modifications that 1) are consistent with the authorized PCCP and can be implemented without a new marketing submission or 2) are not consistent with the authorized PCCP and may require a new marketing submission[114] before the device could be introduced into interstate commerce.

Due to the complexity of AI-DSFs, it is not practical to describe all relevant considerations for a complete PCCP for the limited examples presented below. Therefore, while these examples highlight important concepts that could inform the development and utility of a PCCP, the PCCP will be specific to the circumstances of a particular AI-DSF, based on factors including a scientifically valid assessment of benefits and risks.

FDA recommends that the PCCP strategy be discussed with the appropriate FDA review division through the Q-Submission Program prior to submitting a marketing submission containing a PCCP. As part of a marketing submission, the manufacturer should provide a PCCP, consisting of a Description of Modifications (Section VI.), a Modification Protocol (Section VII.), and an Impact Assessment (Section VIII.).

(1) Patient Monitoring Software

Background:

The device is an AI-DSF intended for use in high-acuity healthcare environments (e.g., an intensive care unit). The software obtains physiological signals (e.g., electrocardiogram, blood pressure, pulse oximetry) from a primary patient monitor. The physiological signals are processed and analyzed by an AI model to detect patterns that occur at the onset of physiologic instability. When physiologic instability is detected, an audible alarm signal is generated to

[114] 21 CFR 807.81(a)(3) or 21 CFR 814.39(a).

indicate that prompt clinical action is needed to prevent potential harm to the patient. The AI-enabled medical device was authorized with a PCCP.

Brief Overview of Pre-Specified Modification:

The manufacturer would like to re-train the AI model with more data to reduce the false alarm rate while maintaining or increasing sensitivity to the onset of physiologic instability. The baseline sensitivity is y%. The manufacturer would like to demonstrate a significant improvement in the false-alarm rate while the sensitivity remains within a pre-specified non-inferiority margin of z% when compared with the original device, i.e., the version of the device without any modifications.[115]

Post-Authorization Modification Scenarios:

Modification Scenario 1: Modification related to quantitative measures of device performance, as specified in the PCCP and implemented in accordance with the PCCP

In accordance with the Modification Protocol, data were collected and used to re-train the AI model. The modified AI model was tested per the methods specified in the Modification Protocol. The results demonstrated that the false alarm rate was significantly reduced while the mean sensitivity estimate was statistically within the proposed non-inferiority margin of the baseline sensitivity y%. Labeling was updated in accordance with the modified AI-DSF performance, and communication was provided to the device users. Because the device modification was specified in the PCCP, and it was implemented in conformance with the PCCP, the device modification would not require a new marketing submission. The manufacturer should document the modification that was specified in the PCCP in accordance with their quality system.

Modification Scenario 2: Modification beyond quantitative measures of device performance, which was not specified in the PCCP

In accordance with the Modification Protocol, the manufacturer re-trained their AI model using additional data to improve the sensitivity. Analytical validation demonstrated that the revised AI model has the same false alarm rate and sensitivity as the previous version. However, the manufacturer also noticed that the modified AI model maintained the same sensitivity and can now also predict physiologic instability in advance of its onset, which the previous version of the AI model could not do. The manufacturer would like to update the device's indications for use to reflect this additional performance claim to predict physiologic instability in advance of its onset, which was not previously included in the PCCP. The methods used for analysis, performance, and statistics were not specified in the PCCP for predicting a future state. Because this modification that was not included in the PCCP could significantly affect the safety or effectiveness of the device, a new marketing submission would be required.

---

[115] The values in this example are shown as variable terms. A completed PCCP should include specific criteria whenever possible.

(2) Skin Lesion Software

Background:

The device is an AI-DSF that analyzes images of skin lesions by identifying and characterizing its features (e.g., color, quantification of area change over time) to aid in diagnosis. It was validated with a specific camera and is intended to be used by a primary healthcare provider. The AI-enabled medical device was authorized with a PCCP.

Brief Overview of Pre-Specified Modification:

The manufacturer would like to extend the AI-DSF for use on additional general-purpose computing platforms, including smartphones and tablets. The general-purpose computing platform must include a two-dimensional camera that meets the minimum specifications defined in the PCCP. The updated device must achieve a minimum performance defined in the Modification Protocol using a specified methodology.

Post-Authorization Modification Scenarios:

Modification Scenario 1: Modification in input, as specified in the PCCP and implemented in accordance with the PCCP

The manufacturer's analytical validation demonstrated that the AI-DSF can be deployed on two additional smartphones that have image acquisition specifications that meet the minimum specifications provided in the PCCP. The analytical performance using the new image acquisition systems was found to be statistically equivalent to the baseline performance, as specified in the Modification Protocol. Labeling was updated to reflect the new AI-DSF compatibility with the additional smartphones, which may increase access of the AI-DSF in the healthcare community. Communication updates on device compatibility were also provided. Because the device modification was specified in the PCCP, and it was implemented in conformance with the PCCP, the device modification would not require a new marketing submission. The manufacturer should document the modification that was specified in the PCCP in accordance with their quality system.

Modification Scenario 2: Modification in input, which was not specified in the PCCP

The manufacturer would like to deploy a modified AI model that uses images captured by a thermographic camera; however, the new camera technology was not specified in the PCCP. Because this modification that was not included in the PCCP could significantly affect the safety or effectiveness of the device, a new marketing submission would be required.

Modification Scenario 3: Modification related to the device's use and performance, which was not specified in the PCCP

The manufacturer would like to distribute a new version of the AI-DSF that is patient-facing. The AI-DSF would provide an analysis of the physiological characteristics of skin lesions, as it

does currently, and direct patients to follow-up with a dermatologist based on the preliminary analysis of the malignancy of the skin lesion. The modification introduces many new, unconsidered risks that were not yet mitigated in the current PCCP, given that the modified AI-DSF will be patient-facing. Because this modification that was not included in the PCCP could significantly affect the safety or effectiveness of the device, a new marketing submission would be required.

(3) Ventilator Settings Software

Background:

The device is an AI-DSF intended for use in the healthcare or home-use setting. The AI-DSF recommends the ideal ventilation parameters based on input data interpretation, which can then be programmed into the ventilator by a healthcare provider. The manufacturer proposes modifications to the AI-DSF to improve performance within the original indications. The AI-enabled medical device was authorized with a PCCP.

Brief Overview of Pre-Specified Modification:

The manufacturer would like to re-train the AI model to optimize site-specific performance for a specific subset of patients with a particular condition, for whom sufficient data were not previously available. Specifically, the manufacturer would like to modify the AI model to improve its ability to optimize ventilator settings for minute volume and tidal volume to reduce the variability to ±x% within the specified range to improve treatment outcomes for that subset of patients at different sites.

Post-Authorization Modification Scenarios:

Modification Scenario 1: Modification related to the device's use and performance in a subset of the patient population, which was specified in the PCCP and implemented in accordance with the PCCP

The manufacturer re-trained and re-validated the AI model on newly acquired data in a subpopulation of patients with a particular disorder. As evidenced by additional clinical performance data collected and analyzed per the Modification Protocol, the re-training on new data improved the reliability and precision of ventilator setting recommendations, showing improvements and specializations to improve site-specific ventilator operation. The updated recommendations were validated against patient outcomes and adverse events that may occur due to ventilator setting inaccuracies following the methods in the Modification Protocol. The adverse event rates and outcomes acceptance criteria were established in the Modification Protocol, and as such, were used to validate the updated AI model. The AI-DSF was updated to implement the re-trained AI model and the labeling was updated for clarity to inform users how the updated AI model accounts for local experience and prevalence. The implementation of this modification was done only at applicable sites. Because the device modification was specified in the PCCP, and it was implemented in conformance with the PCCP, the device modification

would not require a new marketing submission. The manufacturer should document the modification that was specified in the PCCP in accordance with their quality system.

Modification Scenario 2: Modification related to device's use and performance in a subset of the patient population, which was specified in the PCCP, but was not implemented in conformance with the PCCP

The manufacturer re-trained and re-validated the AI model on newly acquired data, but was unable to fulfill the protocol because the manufacturer had to implement a reference standard that was different from the one described in the Modification Protocol. Even though the modification was specified in the PCCP, it was not implemented in conformance with the PCCP. Because this modification that was not implemented in conformance with the PCCP could significantly affect the safety or effectiveness of the device, a new marketing submission would be required.

(4) Image Acquisition Assistance Device

Background:

The AI-DSF is integrated into an imaging system and is intended to assist healthcare providers during acquisition of ultrasound images of the shoulder region in adult and pediatric populations by highlighting portions of the image where it detects a potential abnormality in real time. The AI-DSF interfaces with the device acquisition system, analyzes its output using an AI model, provides real-time alerts to the operator if an abnormality is detected, and automatically adjusts parameters in the device acquisition system during image acquisition to optimize the imaging. The device does not provide a diagnosis. The AI-enabled medical device was authorized with a PCCP.

Brief Overview of Pre-Specified Modification:

The manufacturer would like to re-train their AI model to further optimize the accuracy of the abnormality detection. The PCCP pre-specifies that both the sensitivity and specificity will be shown to be significantly superior for abnormality identification during the shoulder exam.

Post-Authorization Modification Scenario:

Modification Scenario 1: Modification related to quantitative measures of device performance, as specified in the PCCP and implemented in accordance with the PCCP

In accordance with the Modification Protocol, imaging data were collected and used to re-train the AI model. The modified AI model was tested according to a specified test protocol in the Modification Protocol. The results demonstrated that the sensitivity and specificity for abnormality identification met statistical superiority pre-specifications. Labeling was updated in accordance with the modified device performance, and communication was provided to the device users. Because the device modification was specified in the PCCP, and because it was implemented in conformance with the PCCP, the device modification would not require a new

marketing submission. The manufacturer should document the modification that was specified in the PCCP in accordance with their quality system.

Modification Scenario 2: Modification related to the device's use and performance, which was not specified in the PCCP

The manufacturer used new images to re-train the AI model and would like to update their labeling to reflect improved performance in the same shoulder region in a subset of the pediatric patient population identified in the device's indications for use. However, the modification was not specified in the PCCP. Because this modification that was not included in the PCCP could significantly affect the safety or effectiveness of the device, a new marketing submission would be required.

(5) Feeding Tube Placement Radiograph Analysis Software

Background:

The device is an AI-DSF that analyzes chest radiographs from hospitalized patients to evaluate feeding tube placement. The AI-DSF flags images within the radiologist's review queue so that radiographs identified as having a higher likelihood of feeding tube misplacement may be prioritized for reading. The device is designed and validated for specific X-ray machines. The AI-enabled medical device was authorized with a PCCP.

Brief Overview of Pre-Specified Modifications:

The manufacturer would like to improve AI model performance by increasing sensitivity from a baseline of x% (for the original device) to z% (with appropriate confidence intervals) to detect misplaced feeding tubes by re-training on new data. Additionally, the manufacturer would like to expand the compatibility of the device to additional X-ray machines.

Post-Authorization Modification Scenarios:

Modification Scenario 1: Modification related to device's use and performance, as specified in the PCCP and implemented in accordance with the PCCP

The manufacturer re-trained and re-validated the AI model on newly acquired data as described in the Modification Protocol, which significantly improved the AI-DSF sensitivity from x% to z% to detect incorrect feeding tube placements. The analytical performance on new X-ray machines was found to be statistically equivalent to the performance on previously compatible X-ray machines, as specified in the Modification Protocol. Labeling of the device was changed in accordance with the PCCP. Because the device modification was specified in the PCCP, and it was implemented in conformance with the PCCP, the device modification would not require a new marketing submission. The manufacturer should document the modification that was specified in the PCCP in accordance with their quality system.

Modification Scenario 2: Modification related to device's use and performance, which was not specified in the PCCP

The manufacturer used the same database of images to re-train the AI model to identify pneumothorax on chest radiographs. The pneumothorax identification function was found to have the same sensitivity and specificity as the feeding tube AI model. The manufacturer would like to employ the new pneumothorax identification function feature alongside the feeding tube placement AI model in radiograph triage. The modification was not specified in the PCCP. Because this modification that was not included in the PCCP could significantly affect the safety or effectiveness of the device, a new marketing submission would be required.

(6) Optical Imaging System Co-packaged with Imaging Drug

Background:

The product is a device-led combination product including an AI-DSF integrated into an imaging system co-packaged with an approved optical imaging drug. The AI-DSF analyzes images in real-time and highlights potential cancerous lesions for further evaluation. The product was authorized with a PCCP.

Brief Overview of Pre-Specified Modification:

The manufacturer would like to train the AI-DSF to improve speed of the lesion detection. The PCCP specifies that the speed of lesion detection can be improved provided that the sensitivity and specificity do not fall below a pre-specified level.

Post-Authorization Modification Scenarios:

Modification Scenario 1: Modification related to device performance, as specified in the PCCP and implemented in accordance with the PCCP

The manufacturer retrained the AI-DSF using imaging data collected and analyzed in accordance with the Modification Protocol. Analytical validation demonstrated that the modified AI-DSF resulted in image processing speed improvements of 20%. The analytical performance of the imaging system with the increased image processing speed was found to be statistically equivalent to the baseline performance of the imaging system, as specified in the Modification Protocol. Because the device modification was specified in the PCCP, and it was implemented in conformance with the PCCP, the device modification would not require a new marketing submission. The manufacturer should document the modification that was specified in the PCCP in accordance with their quality system.

Modification Scenario 2: Modification related to device's use and performance, which was not specified in the PCCP

The manufacturer would like to deploy a modified AI model that uses data from a patient population that was not included in the intended use population. The modification was not specified in the PCCP. Because this modification that was not included in the PCCP could significantly affect the safety or effectiveness of the device, a new marketing submission would be required.

Modification Scenario 3: Modification related to device's use, which was not specified in the PCCP

The manufacturer would like to distribute a new version of the AI-DSF that is used with a modified dosing regimen of the drug. This modification was not specified in the PCCP. Because this modification that was not included in the PCCP could significantly affect the safety or effectiveness of the device, a new marketing submission would be required.

Also note that, consistent with the scope of this guidance (see Section III.), the recommendations in this guidance do not apply to modifications to the drug or biologic constituent part of device-led combination products.
